# Design of intrinsically disordered region binding proteins

**DOI:** 10.1126/science.adr8063

**Published:** 2025-07-17

**Authors:** Kejia Wu, Hanlun Jiang, Derrick R. Hicks, Caixuan Liu, Edin Muratspahić, Theresa A. Ramelot, Yuexuan Liu, Kerrie McNally, Sebastian Kenny, Andrei Mihut, Amit Gaur, Brian Coventry, Wei Chen, Asim K. Bera, Alex Kang, Stacey Gerben, Mila Ya-Lan Lamb, Analisa Murray, Xinting Li, Madison A. Kennedy, Wei Yang, Zihao Song, Gudrun Schober, Stuart M. Brierley, John O’Neill, Michael H. Gelb, Gaetano T. Montelione, Emmanuel Derivery, David Baker

**Affiliations:** 1 Department of Biochemistry, University of Washington, Seattle, WA, USA.; 2 Institute for Protein Design, University of Washington, Seattle, WA, USA.; 3 Biological Physics, Structure and Design Graduate Program, University of Washington, Seattle, WA, USA.; 4 Department of Electrical Engineering and Computer Science, University of California, Berkeley, CA, USA.; 5 Department of Chemistry and Chemical Biology, Center for Biotechnology and Interdisciplinary Studies, Rensselaer Polytechnic Institute, Troy, NY, USA.; 6 Department of Chemistry, University of Washington, Seattle, WA, USA.; 7 MRC Laboratory of Molecular Biology, Cambridge, CB2 0QH, UK.; 8 Howard Hughes Medical Institute, University of Washington, Seattle, WA, USA.; 9 Visceral Pain Research Group, Hopwood Centre for Neurobiology, Lifelong Health Theme, South Australian Health and Medical Research Institute (SAHMRI), North Terrace, Adelaide, South Australia, Australia.; 10 Faculty of Health and Medical Sciences, University of Adelaide, North Terrace, Adelaide, South Australia, Australia.

## Abstract

**INTRODUCTION:**

Intrinsically disordered proteins and peptides play key roles in biology, but the lack of defined structures and high variability in sequence and conformational preferences has made targeting such systems challenging. Peptide-specific antibodies have been obtained by immunization or library selection, but these methods require considerable effort and disordered antigens are susceptible to degradation following injection. In silico design of proteins that can recognize unfolded peptides based on their sequence is thus an important challenge.

**RATIONALE:**

We sought to develop a method for achieving specific recognition of an intrinsically disordered region sequence of interest that would be broadly useful for applications in proteomics, targeting, sensing, and sequencing. We reasoned that a general solution to the intrinsically disordered region binding problem could employ an induced fit–based binding strategy, taking advantage of the fact that disordered protein regions should be flexible and lacking in preferred secondary structure. We set out to generate a set of designed binding proteins in complex with peptide backbones in a variety of conformations, with sufficient diversity to accommodate any target disordered amino acid sequence. We aimed to do this by combining physical-based and deep learning–based design methods. Using classical design methods, we first generated extended repeating protein scaffolds with pockets specialized for repeating peptide sequences. We then recombined the pockets and generalized them to a wide range of sequences using the deep learning RFdiffusion approach. Given such a set of designed binder-target peptide templates, we reasoned we could achieve general recognition of disordered protein regions by threading them through each of the templates to search of the optimal binding modes. For the most favorable matches, we used machine learning–based sequence design and backbone refinement methods to optimize binder affinity.

**RESULTS:**

We tested our approach by designing binders for 18 synthetic peptide sequences and 21 broadly diverse, therapeutically relevant, intrinsically disordered regions. The designs were expressed and purified, and binding to the targeted disordered regions was measured by biolayer interferometry, with most designs showing picomolar to nanomolar dissociation constants. We obtained binders for 39 of 43 targets from this one-shot design process, testing 22 designs per target, on average. All-by-all binding experiments showed that each design only binds tightly to the target it was designed to bind. We showed that the binders can enrich low abundance proteins from cellular lysates for proteomics analyses, target disordered regions of extracellular receptors implicated in cancer, antagonize G protein coupled receptor signaling, and drive protein localization inside cells.

**CONCLUSION:**

Our computational design pipeline enables the design of binding proteins to arbitrary disordered peptides and proteins. Although targeting disordered proteins has been a considerable challenge for traditional methods, we show that the disorder is an advantage: The designed binding protein drives the target sequence into a privileged binding-competent conformation, with, for example, the hydrophobic residues primarily on one face and the polar residues in customized binding modes. For each target, we sampled a wide variety of conformations and identified those compatible with high affinity binding. This approach contrasts with folded targets, whose fixed structures may admit few optimal binding solutions. Our approach should be broadly useful for designing binders for arbitrary disordered protein regions of interest.

Intrinsically disordered proteins and peptides play key roles in biology, but a lack of defined structures and high variability in sequence and conformational preferences have made targeting such systems challenging. We describe a general approach for designing proteins that bind intrinsically disordered protein regions in diverse extended conformations with side chains fitting into complementary binding pockets. We used the approach to design binders for 39 highly diverse unstructured targets, including polar targets, and obtained designs with 100-picomolar to 100-nanomolar affinities in 34 cases, testing ~22 designs per target. The designs function in cells and as detection reagents and are specific for their intended targets in all-by-all binding experiments. Our approach is a major step toward a general solution to the intrinsically disordered protein and peptide recognition problem.

## Graphical Abstract

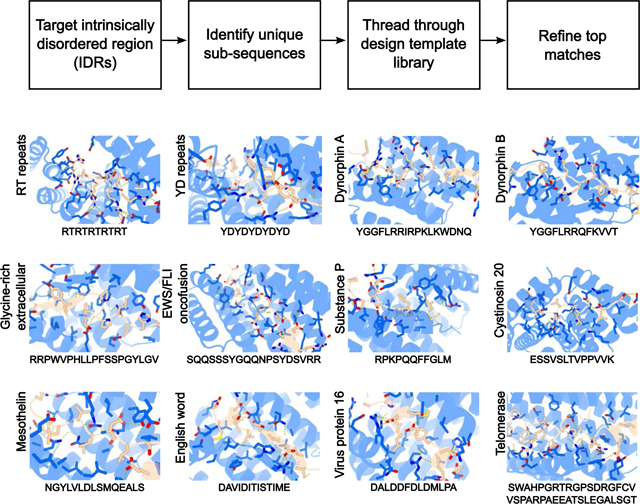

**Design of IDR binding proteins**. Design pipeline and 12 representative examples of active designs (for each panel, the target name is at left and the target sequence is at the bottom; targets are tan and binders are blue in the structure models. Interface residues are shown as sticks).

Natural evolution has generated a variety of solutions to the challenge of binding unstructured regions of peptides and intrinsically disordered proteins (IDPs) (1–6), including natural antibodies (1–3), major histocompatibility complexes (4), tetratricopeptide repeats (5), Armadillo repeat proteins (6), and lipocalins (Anticalins). Despite this diversity (1–6), engineering general peptide recognition remains challenging; peptide-specific antibodies have been obtained by immunization or by library selection but this requires considerable effort and disordered antigens are susceptible to degradation following injection. There has been progress in generalizing the binding modes of armadillo repeat and other natural peptide-binding proteins (3–5), but achieving completely new specificities has been challenging. De novo protein design methods have been used to design proteins that bind peptides in polyproline II, alpha-helical, and beta-strand conformations (7–9), but more general recognition of disordered proteins and peptide regions requires the ability to bind more varied conformations as an arbitrary disordered sequence may not have the propensity for the same secondary structure throughout, or present suitable interfaces for binding in regular secondary structures. For example, amphipathic helices or strands can be recognized using designs with grooves that bind primarily to the nonpolar side of the helix or strand, but if charged residues are distributed around the helix or strand axis, this binding mode would require energetically unfavorable charge burial. A method for achieving specific recognition of any target intrinsically disordered region (IDR) sequence of interest would be broadly useful for applications in proteomics, targeting, sensing, and sequencing.

We reasoned that a general solution to the IDR binding problem might be achieved by combining the strengths of physical and deep learning design approaches. Rosetta design methods have been used to design binding proteins consisting of four to six tandemly repeated structural units that bind repeating proline-rich sequences in the polyproline II conformation, with each repeat unit in the designed binder interacting with a repeat unit in the peptide (Fig. 1A). Generative deep-learning free-diffusion methods (8, 10) have been used to generate binding proteins with no such preimposed structural constraints on the binder structure (Fig. 1B). However, because the model is trained on the Protein Data Bank (PDB), this procedure generally folds the target sequence into the alpha-helical or beta-sheet conformations that dominate the PDB, which as noted above can have poor compatibility with binding. The repeat protein-repeat peptide approach can in principle be generalized beyond polyproline II conformations, but is limited by the requirement that each peptide unit have the same conformation, which again may be incompatible with heterogeneous target sequences. We reasoned that starting from different repeat protein architectures and recombining and specializing amino acid binding pockets in different repeat units for different amino acids and different conformations using diffusion could yield a family of templates enabling more general recognition of sequences with widely varying conformational preferences and sequences (Fig. 1C). In the following sections, we first describe the creation of such a scaffold library for general peptide recognition (Fig. 1, D to F), and then the use of the library to design binders for a wide variety of nonrepeating, both synthetic and native, IDR targets (Fig. 1, G to J).

## Generation of template library

**Fig. 1.**
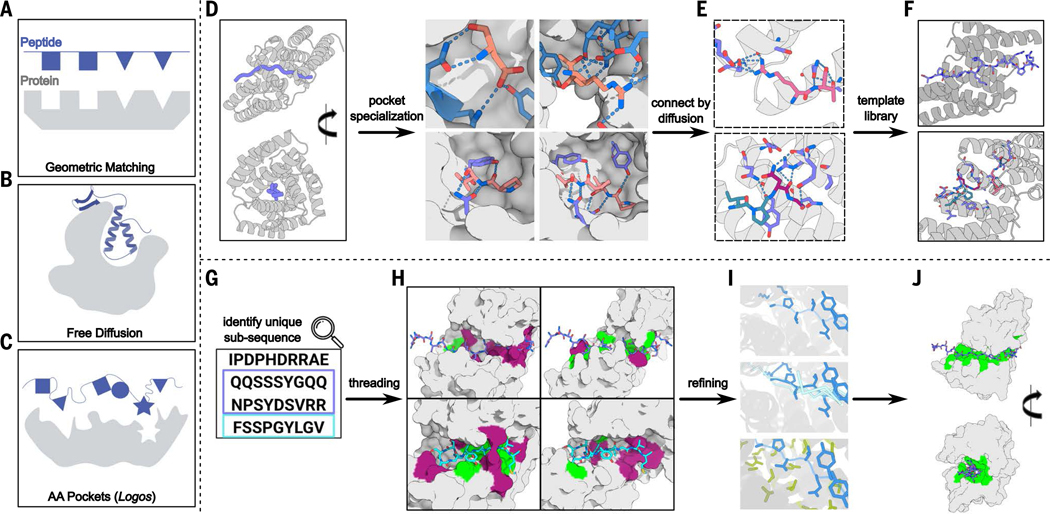
Overview of IDR binder design protocol. (**A** to **C**) Design methods. (A) Repeat protein–based geometric matching approach requiring one-to-one matching between identically spaced repeat units on designed binder and target peptide. (B) Unconstrained free diffusion approach folds targets into structures frequently observed in the PDB training set, primarily helices but also strands. (C) The amino acid (AA) pockets approach explored here combines the designed pockets and extended scaffolds of the geometric matching approach with the ability of RFdiffusion to recombine and diversify the pockets to achieve more general recognition of nonrepeating sequences. (**D** to **F**) Template library construction. (D) (Left) Designed binder scaffolds wrap around extended peptide backbones, enabling contact with each target amino acid. (Right) Example binding pockets. (E) Binding pockets are connected using RFdiffusion (each peptide window is colored differently, as purple, pink, and blue) into templates for general sequence recognition. (F) Examples of two of the 1000 generated templates. (**G** to **J**) IDR binding pipeline. (G) Unique subsequences (purple and cyan) were identified through a protein sequence database search and (H) threaded through the template library to identify optimal matches between amino acid segments and binder pockets. Pocket matches are green and mismatches are dark red on the protein surface. (I) Matches are refined using “one-sided partial diffusion” (top), where only the binder is changed; “two-sided partial diffusion” (middle), where the target and the binder can be changed; “motif diffusion” (bottom), where key interacting motifs (target, blue; binder, green) are unchanged while the rest are noised, reconnected, diversified, and optimized. (J) Examples of resulting designs. [Panels (A) to (C) were created with BioRender.com]

We reasoned that the template library should have two properties: First, each template structure should “wrap” around extended peptide conformations with numerous opportunities for the hydrogen bonding and packing interactions with the target required for high specificity (Fig. 1D). Second, the structural variation in the template family should be sufficiently broad that for any target sequence, at least one of the templates is able to induce it into a defined binding-competent conformation.

We developed a three-step approach to generating such a library of template structures suitable for general recognition. In the first “scaffold generation” step (Fig. 1D and methods I), we design repeat proteins that wrap around peptides in different repeating conformations, such that each repeat unit on the protein forms a binding pocket that interacts with a corresponding repeat unit on the peptide. We require that these pockets have side chains that not only interact with the target side chains but also make hydrogen bonds with the target backbone to provide structural specificity and compensate for the cost of desolvation. In the second “pocket specialization” step (Fig. 1D and methods II), we fine-tune these pockets using diffusion to achieve more precise matching to specific target peptide sequences. We keep the four to nine amino acids surrounding each sidechain bidentate hydrogen bond from the repeat protein to the peptide backbone fixed (fig. S1) while diversifying the hydrophobic interactions between designed binders; this is advantageous because hydrogen bonding interactions have more stringent geometric requirements than nonpolar packing interactions and hence are more efficiently templated than repeatedly sampled from scratch (see methods II4). In the third “pocket assembly” step, we go beyond the limitations of repeating structures, which are optimal for repeating sequences but not more general sequence targets, by recombining pockets from different designs, using RFdiffusion (11) to generate interfaces between them where necessary to yield overall rigid structures (Fig. 1F). This generates a set of templates with diverse pockets arranged in different orders and geometries (Fig. 1F and methods III).

**Fig. 2.**
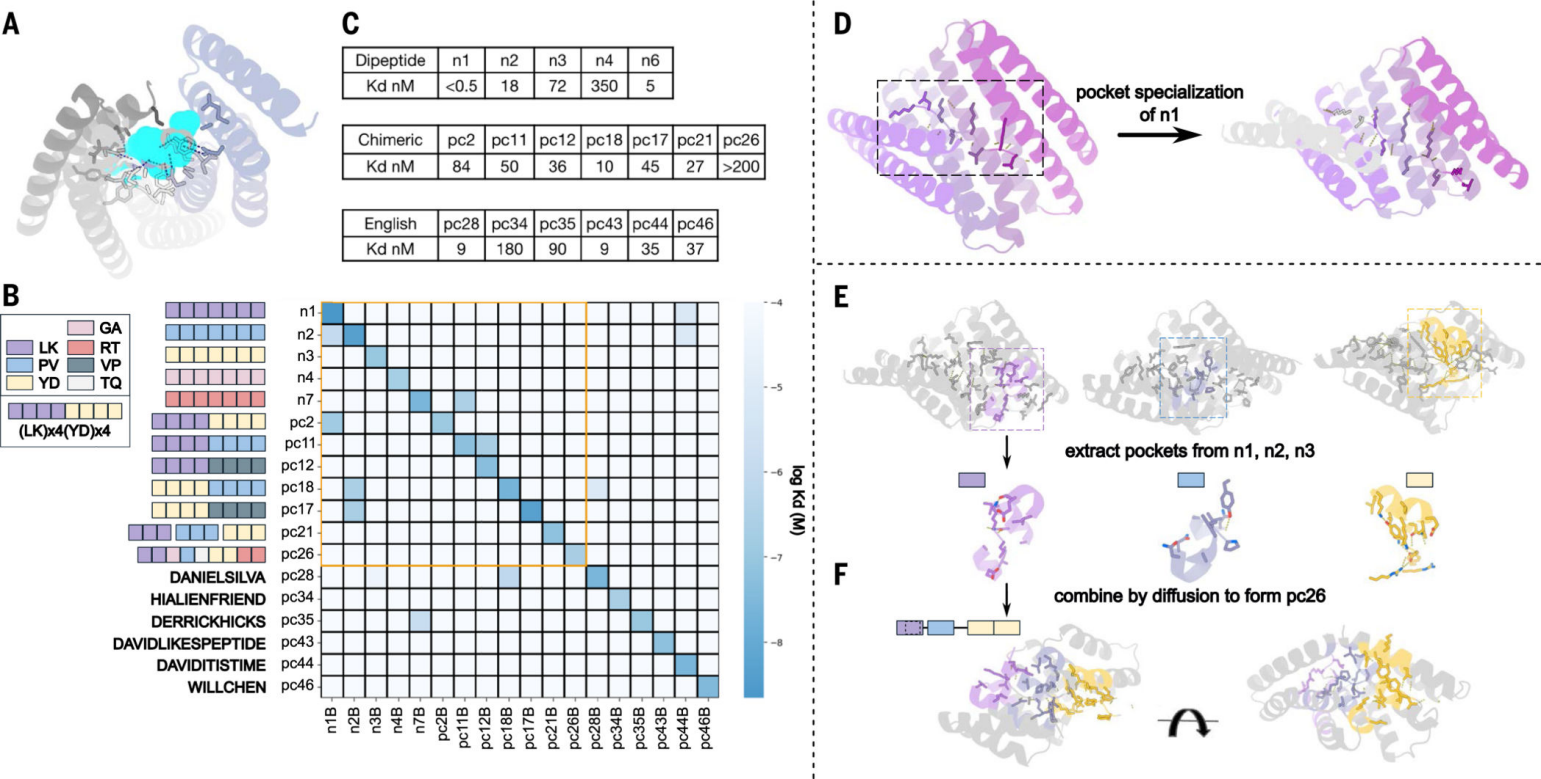
Designs binding to 18 synthetic peptides. (**A**) Representative binder design (illustration) wrapping around a peptide (cyan) in an extended conformation (hydrogen bonds are shown as dotted lines). (**B**) All-by-all binding K_d_s obtained from nanoBiT binding titrations for 18 designed binder-synthetic peptide pairs. Target sequences are on the *y*-axis and binders are on the *x*-axis, with each square representing one dipeptide motif using the color scheme in the legend (left). Heatmap intensities indicate K_d_ averages from two titration experiments. Pairs within the orange square are composed of similar dipeptide repeats and hence have more crosstalk. (**C**) Cognate-designed binder-target K_d_s measured by biolayer interferometry. Peptide label-identity pairings are on the *y*-axis in (B). Two to 35 binder designs were experimentally tested per target. (**D** to **F**) Library construction example using pc26. The three recombined pockets are shown using color codes from (B). (D) “Pocket specialization”. Following sequence threading, the originally identical binding pockets are specialized for the adjacent target amino acid segment using motif diffusion. In this example, optimizing and extending a perfectly repeating four-repeat scaffold (left) generates a new five-repeat scaffold (right). The new extended fifth repeat is shown in light gray. (E) Binding pockets and contacting peptide segments (two-sided interaction motifs) are extracted from sets of optimized scaffolds, in this case n1, n1 and n3. (F) The extracted two-sided binding motifs are connected into coherent binding proteins using RFdiffusion with varied spacers and angles. An example of an assembled design is shown at the bottom.

For the first “scaffold generation” step, we chose to target peptides in a broad range of extended conformations rather than solely the polyproline II conformation as in our earlier study, because this is populated primarily by proline-rich peptides. In extended conformations, alternating side chains face in opposite directions, consistent with a two-residue sequence repeat. We used Rosetta design methods as described previously to generate designs targeting the dipeptide repeats LK, RT, YD, PV, and GA (single-letter amino acid codes) in a variety of extended conformations that wrap around these peptides such that each repeat unit interacts with one dipeptide unit (Fig. 2A and methods I). Experimental characterization by fluorescence polarization of four-repeat versions of the designed binders revealed nanomolar binding for the LK and PV repeat peptides, but little binding for the more polar RT and YD and no hits for the highly flexible GA (fig. S2; to avoid potentially unfavorable interactions with peptide termini, for experimental testing here and below, we pad all repeat target peptides with two additional repeats).

For the second “pocket specialization” step, we refined the designed binding pockets to improve the fit to the target sequences and extended the number of interacting repeat units from four to five to further increase affinity. This yielded designs with picomolar affinities for LK repeats and low nanomolar affinities for RT and GA repeats (Fig. 2, B and C, and fig. S3). The binding pockets in these designs have distinct geometries that are customized to the target being recognized (see example in fig. S4).

For the third “pocket assembly” step, we enable more general recognition of nonrepeating sequences by assembling the binding pockets into new backbones, keeping them positioned to interact with peptide targets in continuous extended conformations (Fig. 2, D and E). We assembled combinations of two to six binding pockets in silico (see below and methods III), yielding models of chimeric proteins interacting with chimeric peptide targets. To do this, we positioned pockets parametrically (see methods III, 1) and connected them by means of RFdiffusion. We used this approach to generate 70 designs against seven chimeric targets. We refer to each binding unit (comprising a single amino acid or dipeptide and corresponding designed protein pocket) with a letter; thus, AAABBB is a chimera of two designs from the previous section whereas ABCDEF combines six different pockets. Experimental characterization using NanoLuc Binary Technology assay (nanoBiT) split luciferase reconstitution (12) and biolayer interferometry (BLI) showed double-digit nanomolar binding for six of the seven targets, out of only 10 designs tested per target on average (Fig. 2B and fig. S4).

To increase the size of the template library to cover a broader range of sequences, we used pocket assembly to build 36 chimeric backbones containing pockets recognizing polar residues, and further diversified both binder and peptide target by two-sided sequence design in silico (see methods III, 3; in the designs described above, the peptide sequence was always held constant). Together, this yielded a library of 1000 templates, each consisting of a designed binding protein and a corresponding peptide backbone positioned such that the amino acids in the peptide fit into designed pockets in the binding protein (representative examples shown in fig. S5).

## Threading intrinsically disordered regions onto the template library

We developed a two-step approach for using the template library to generate binders for nonrepeating synthetic sequences and arbitrary native unstructured targets. In the first “threading” step (Fig. 1G), the target sequence is threaded through the backbone of each template to identify the most compatible sequence segment-template pairs. In the second “refinement” step, the best matches are refined to increase the fit between the designed binder and target peptide (Fig. 1, H and I).

For an IDP or IDR, there are, in general, a large number of possible peptide subsequences that can be targeted. To identify the most targetable peptide subsequences within an IDR, we first discard segments with low sequence complexity and/or those with multiple close matches in the proteome (Fig. 1G and figs. S6 and S7), as binders to such targets would likely have some cross reactivity. We map each of the remaining unique sequence segments of 8 to 40 amino acids onto each of the target backbones in the library, carry out local backbone resampling, optimize the sequence of the binder using ProteinMPNN, and evaluate the designs based on the fit between the designed binder and the target sequence and the agreement between the AF2 prediction and the design model (see methods IV). This approach maps target segments with multiple polar residues into templates compatible with extended hydrogen bonding networks, which is likely important for achieving general recognition. In cases where AF2 metrics were suboptimal, we used RFdiffusion (see methods V) to customize the backbone for the specific target.

We first tested this approach on synthetic sequences corresponding to six arbitrarily selected English words and names. We tested 45 designs against six targets, eight designs per target on average; the best binders for two out of the six targets had single-digit nanomolar affinities (K_d_s = 9 nM); three had double-digit affinities (K_d_ = 35 nM, 37 nM, and 90 nM); and one had K_d_ = 180 nM (Fig. 2B and fig. S3). We investigated the selectivity of the designs for their peptide targets by carrying out all-by-all (18 by 18) nanoBiT interaction measurements, including the repeat sequence binders of the previous section. Although there was some crosstalk between designs and targets with related sequences (for example, designs targeting four PV repeats also bound peptides with eight PV repeats), for the more diverse targets, the designs were largely orthogonal (Fig. 2C and table S1).

**Fig. 3.**
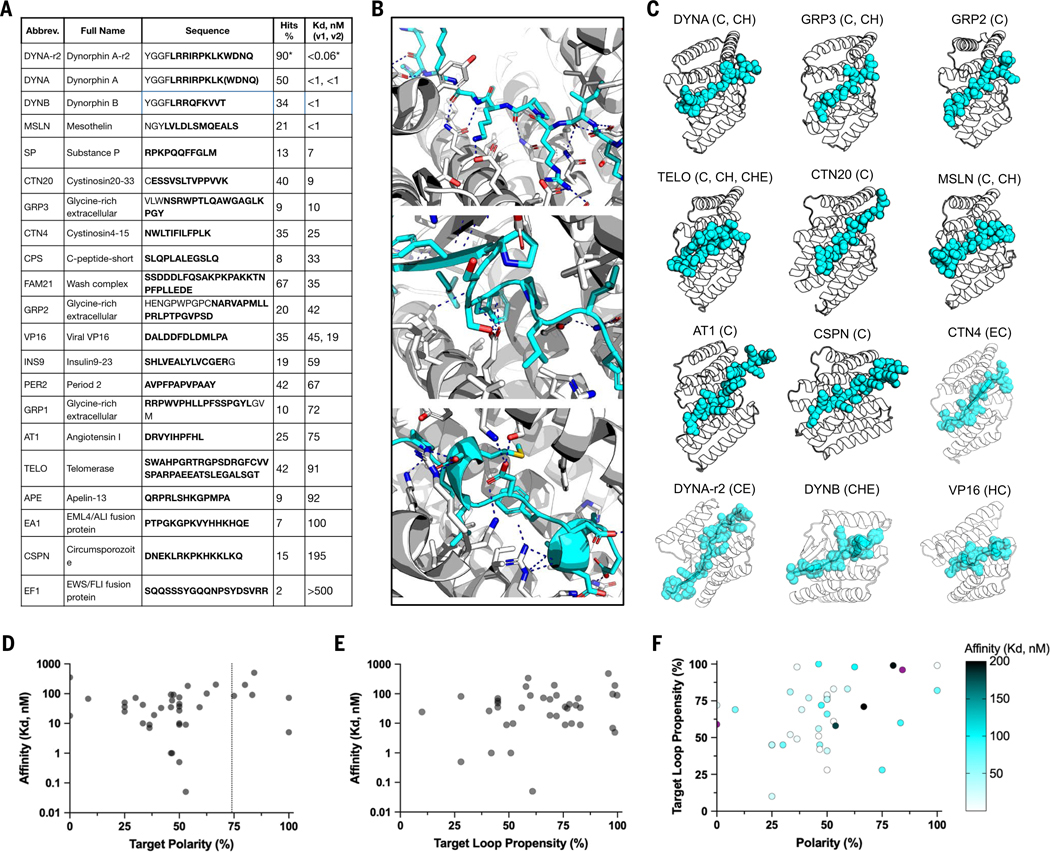
Designs binding to 21 native protein disordered regions and peptides. (**A**) Targeted native bioactive peptides and IDRs; bold indicates the targeted sequence segment. Best-obtained K_d_s are ranked from low to high; for the same target, the best two K_d_s from binders targeting two distinct target conformations are separated by commas. Hit rate is the percentage of tested designs showing binding signals [>0.1 arbitrary units (AU)] on BLI at 500 nM. Asterisks indicate statistics calculated from an optimization campaign instead of a one-shot campaign. Three to 48 designs were experimentally tested per target. (**B**) Interactions of representative IDR binder conformations: random coil conformation (top); strand-containing conformation (middle); helical-containing conformation (bottom). (**C**) Models of representative designed complexes. Abbreviations of the targets are above each model, with the targeted secondary structure content in parentheses. C, random coils; H, (partial) helix; E, (partial) strand. In cases where multiple distinct conformations were targeted, their secondary structures are separated by commas. (**D**) Target polarity versus highest achieved affinities. Polarity is calculated based on the percentage of polar and charged amino acids among the targeted windows. (**E**) Target loop propensity calculated with AIUPred3 algorithms versus the highest achieved affinities. (**F**) Target polarity versus target loop propensity with the affinity represented by a color gradient (dark blue, ≥ 200 nM; black, ≤ 1 nM).

We next used the threading approach to generate binders for 21 diverse therapeutically relevant IDPs, IDRs, and segments of IDPs ranging from eight to 40 amino acids. These include eight GPCR ligands, two insulin-related ligands, four disease detection-related disordered regions, four IDRs from cancer-related receptors, and three human scaffolding complexes for which there are no good monoclonal antibodies (target names and targeted sequences are shown in Fig. 3A and target sequence properties are shown in fig. S8). For each target, three to 48 designs (on average 28) for which the AF2 predicted structures of the complexes were close to the computational design models (fig. S10) were selected for experimental characterization. The designs have overall helical architectures reflecting their helical repeat protein origins (fig. S9), with considerable structural variation in some regions introduced by the diffusion assembly and refinement steps, whereas the target sequences adopt a wide range of random coil and partial helical and strand conformations (Fig. 3, B and C, and fig. S10).

The designed binders were expressed and purified, and binding to the targeted IDRs was measured by BLI with the binder in solution and the peptide attached to the sensor chip. The fraction of binders that showed a binding signal at 500 nM ranged from 2 to 67% (Fig. 3A). Together with the 18 synthetic targets, we obtained binders for 39 of 43 targets, testing 22 designs for each target on average (table S2).

Polar targets have long been considered challenging in protein design. Of the targets, 20 had >50% polar residues and six had >75%; binders were obtained for the fusion fragment EF1 of EWS/FLI oncofusion protein for Ewing sarcoma (13–15) (84% polar residues), and the N-terminal fusion fragment CSP-N of Circumsporozoite protein (CSP) for malaria (16) (80% polar residues) (see Fig. 3D for target polarity distribution). The number of hydrogen bonds made to the side chains of the target (per 10-amino-acid segment) was three times higher for the highly polar targets than that of the other targets (4.3 for target polarity <75%; 12.3 for target polarity ≥75%). 77% of the targets had little predicted secondary structural propensity in isolation and 87% adopted predicted bound conformations lacking extensive secondary structure; all adopted conformations were very different from those in previously solved crystal structures in cases where these were available (Fig. 3E and table S3). Binding affinities were determined by global fitting of BLI association and dissociation phases at a range of binder concentrations. There was little correlation between binding affinity and the polarity or intrinsic secondary structure of the target; K_d_s <10 nM was achieved for targets with a wide range of polarities and secondary structure propensities (Fig. 3, D to F, and table S4), indicating the generality of the method.

**Fig. 4.**
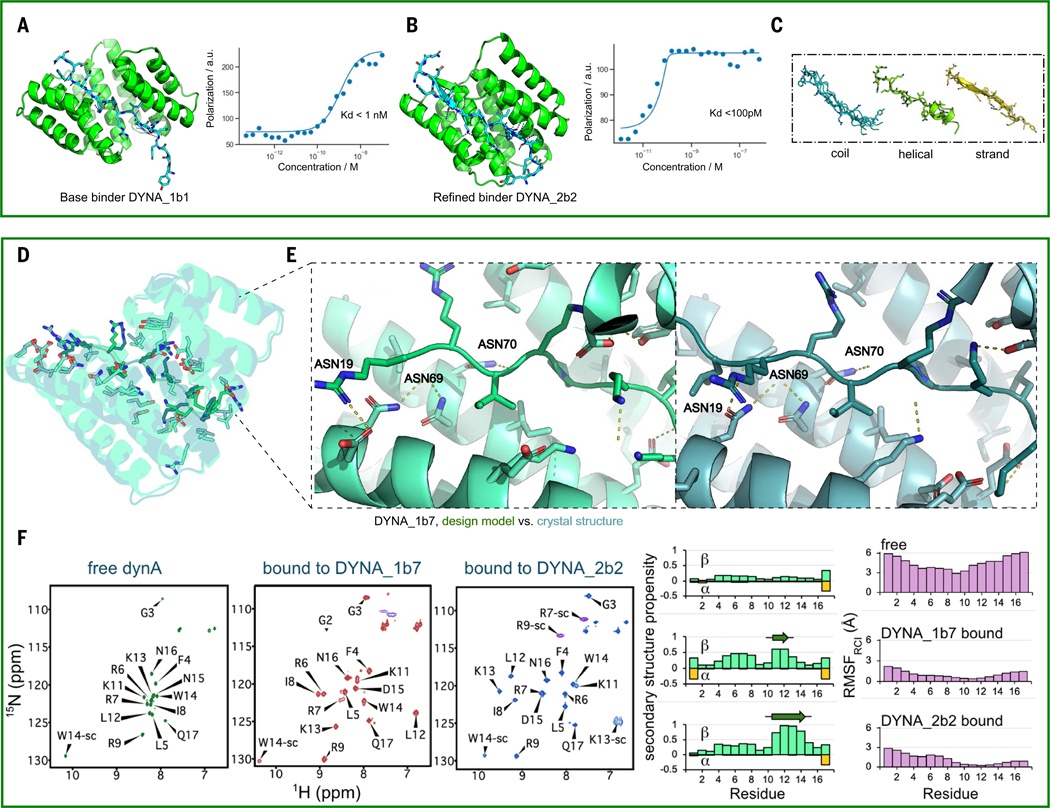
Structural characterization of dynorphin A binder designs. (**A**) Design model of DYNA_1b1 bound to dynorphin A in an extended backbone conformation, with five pairs of peptide backbone-protein sidechain bidentate hydrogen bonds (left). Fluorescence polarization (FP) binding with 1-nM TAMRA-labeled peptide indicates a K_d_ < 1 nM. (**B**) Diffusion-refined binder DYNA_2b2 with estimated K_d_ < 100 pM by fluorescence polarization with a 120-pM peptide (K_d_ cannot be accurately measured below the concentration of peptide used in the FP assay). (**C**) During diffusion-based refinement, the target peptide backbone and binder were resampled around random coil conformation (left), partial helical conformation (middle), and partial strand conformations (right). (**D**) Superposition of the zoomed-out computational design model (green) and the 3.15-Å co-crystal structure (cyan) of dynorphin A bound with design DYNA_1b7, with interface residues shown as sticks. (**E**) Zoom-in of the design model (green, left) and crystal structure (cyan, right) in the center of the designed interface. (**F**) (Left) Assigned NMR ^1^H-^15^N Heteronuclear Single Quantum Coherence (HSQC) spectra of ^15^N^13^C-labeled dynorphin A unbound (free), bound to unlabeled DYNA_1b7 and bound to unlabeled DYNA_2b2 (a variant of DYNA_2b1) in solution, and (right) secondary structure propensity and C RMSF (RMSF_RCI)_) based on backbone chemical shift data. Sidechain Asn and Gln amide resonance peaks in these HSQC spectra are not labeled. When complexed with DYNA_2b2, the dynorphin Lys13 side chain (sc) amino group (K13-sc) in the 15N dimension at 72.8 ppm, is observed because it is stabilized by hydrogen bonding to 2b2 and buried within the complex, is observed because it is stabilized by hydrogen bonding to 2b2 and buried within the complex.

To explore the optimization potential of the designs, we chose a binder, DYNA_1b1, with a K_d_ of ~1nM for dynorphin, a kappa opioid receptor (KOR) peptide ligand implicated in chronic pain (17, 18). We used RFdiffusion refinement on top hits as described above for the synthetic targets (Fig. 4A, 2C, and methods V). Out of 48 designs, 45 showed strong binding in the BLI screening assay at 5 nM, and six had K_d_ ≤ 100 pM by BLI; fluorescence polarization measurements for two of these optimized designs, DYNA_2b1 and DYNA_2b2, indicated K_d_s <60 pM and <100 pM, respectively (Fig. 4B and fig. S11). Over the set of original and optimized designs for dynorphin A, the peptide populated a wide diversity of random coil, partial strand, and partial helix conformations (Fig. 4C). The dynorphin A and B binders were orthogonal, binding only to their intended targets (see below), despite having 62% sequence similarity.

## Structural validation

We succeeded in solving a co-crystal structure of a 7-nM-K_d_ dynorphin A binding design, DYNA_1b7, in complex with dynorphin A (residues 1 to 17) at 3.15 Å resolution (Fig. 4, D and E, and fig. S12). The backbones of both the protein and peptide in the crystal structure match the design model well, with an interface backbone RMSD of 1.2 Å for the complex and interface sidechain RMSD of 2.9 Å (Fig. 4, D and E). The key interactions are in the central region of the peptide (Fig. 4E): During design, we excluded the N-terminal YGGF sequence, which is shared between dynorphin A and B and other neuropeptides (19), aiming to distinguish closely related peptides in the family in which antibodies often fail, and the C-terminal region (-WDNQ) which is missing in some species and hence was not targeted. For this design, the two-sided diffusion refinement eliminated several of the asparagine-peptide backbone bidentate interactions, which could account for the decrease in binding affinity from ~1 nM for the starting design to 7 nM (fig. S12; see fig. S13 for the peptide density maps). In the crystal structure, all of the hydrogen bonds in the design model of DYNA_1b7 made to the peptide backbone (ASN19, ASN69, and ASN70 on binder) were present as designed; the corresponding peptide region (from LEU5 to ARG9) also aligned precisely to the design with Cɑ root mean square deviation (RMSD) = 0.6 Å. There were minor shifts of side chains in hydrophobic grooves and density was missing for the termini that were not included in the design calculations (YGG- and -DNQ).

To investigate changes in dynorphin structure upon binding, we examined the nuclear magnetic resonance (NMR) spectra of isotope-labeled dynorphin A unbound in solution, bound to DYNA_1b7 (K_d_ = 7 nM) and to the higher affinity design DYNA_2b2 (K_d_ <200 pM; Fig. 4F and fig. S11). NMR confirmed that free dynorphin A is intrinsically disordered and becomes ordered upon binding, except for the regions not included in the design (Fig. 4F). For both bound complexes, the NMR data indicated an extended bound-state conformation, consistent with the design models (Fig. 4F and fig. S14). The extent of ordering upon binding to DYNA_2b2 was greater than that for DYNA_1b7 in both the C-terminal region and around the TRP14-ASN137 bidentate interaction, consistent with the more extensive sidechain-backbone hydrogen bonding in the former (Fig. 4F and fig. S14).

The extended conformation of the dynorphin peptide in the designed complexes, confirmed by the x-ray and NMR data, is considerably different from any previously solved cryo–electron microscopy or NMR structures of dynorphin with native KOR (fig. S14), where it is bound in a compact, partial helix conformation (PDB ID 2n2f, 8f7w) (20, 21). These data highlight the power of computational design for inducing disordered proteins and peptides into non-native conformations.

To assess the contributions of each target residue to binding, we carried out alanine scanning experiments on the dynorphin A binder DYNA_1b1-dynorphin interaction. In the nanoBiT binding experiments, each of the nine alanine substitutions on the peptide that disrupt interactions with the binder considerably reduced the extent of binding compared with the wild-type dynorphin peptide (fig. S15).

## Applications of designed binders

**Fig. 5.**
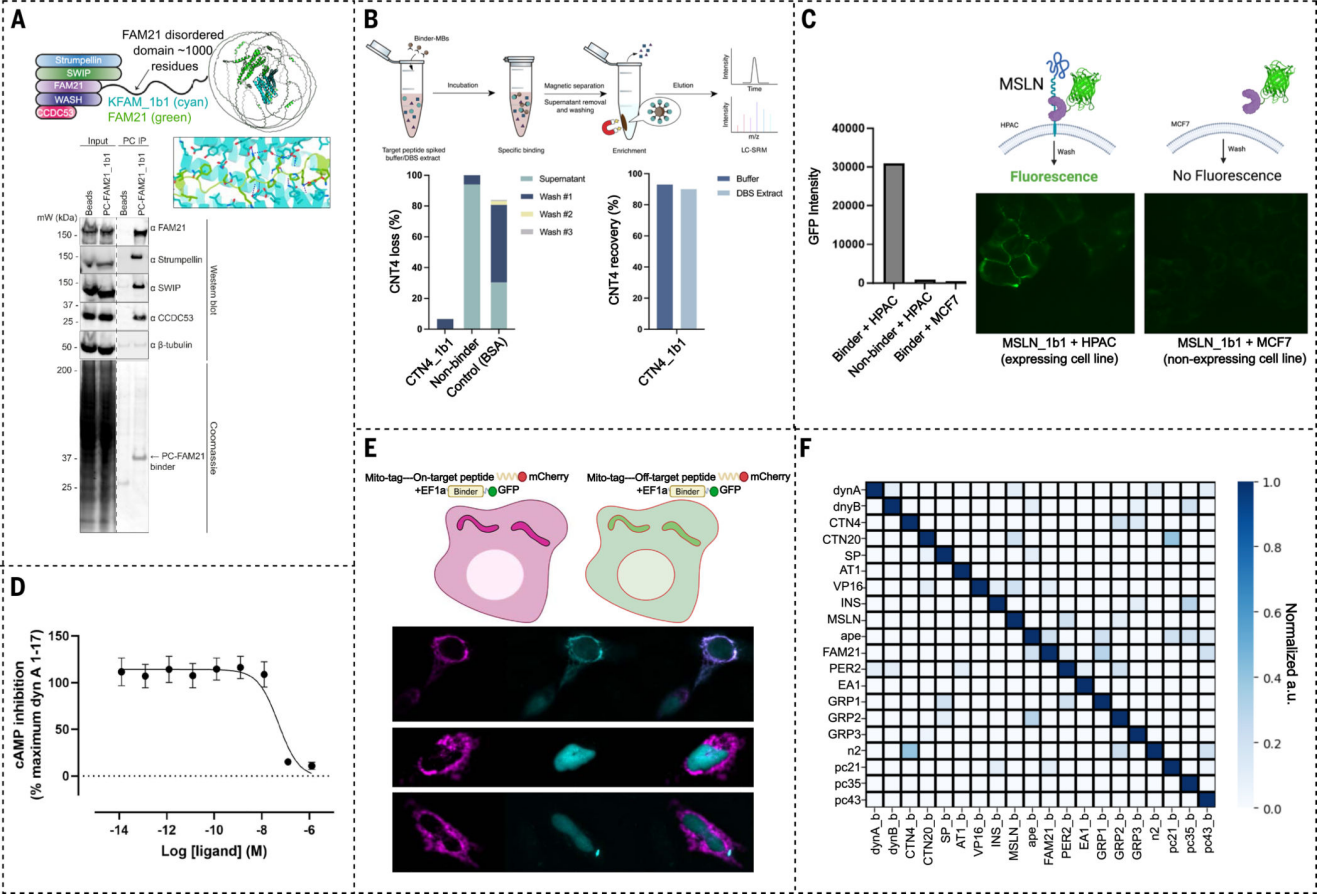
Designed binders are functional and orthogonal. (**A**) (top) The WASH complex contains the FAM21 protein with a long disordered tail (Middle; cyan and green). AF2 predicted FAM21 complex with designed binder KFAM_1b1. (Bottom). Cell lysates were immunoprecipitated with designed binders and the bound proteins were assessed by Coomassie stain and Western blot. Designed binder PC-FAM21_1b1 immunoprecipitated FAM21 and other WASH complex subunits from the cell lysate. β-tubulin was blotted as a loading control. (**B**) Illustration of the use of BSA-blocked designed binder-conjugated magnetic beads (MBs) to capture trypsin-generated target peptides for the case of cystinosin. The amount of peptide recovered from elution was quantified by LC-MS. (Left) Percentage of unbound peptide from each step normalized to the peak area of peptide standards. (Right) Percentage of peptide recovery by LC-MS and normalized to the peak area of peptide standards. Nonbinder MBs and BSA-blocked unfunctionalized MBs were used as negative controls. (**C**) Designed binder–GFP fusions specifically recognize MSLN targets on cells. MSLN_1b1-GFP staining is observed on the cell surface in MSLN-expressing (HPAC) but not in non-MSLN-expressing (MCF7) cell lines following incubation at 1 uM. No signals were observed when incubating HPAC cells with a nonbinder-GFP fusion. Data shown are a single representative experiment. (**D**) Antagonism of dynorphin A–stimulated KOR signaling by DYNA_2b2 binder competition measured in a cAMP assay in CHO cells. Data are shown as mean ± SEM (*n* = 4). The IC_50_ of DYNA_2b2 binder was 50.3 ± 0.7 nM. See figs. S19 and S20 for activation mechanisms. (**E**) Colocalization of designed binders with targets in cells. Target proteins fused to mCherry and a mitochondria-targeting sequence (Mito-Tag), and binders fused to GFP, were expressed in HeLa (CCL-2) cells. The GFP signal is only relocalized to the mitochondria for designs with their cognate targets as shown in row one (target DYNA versus binder DYNA_1b1). Off-targets with point mutations in the target in row two (mutant target DYNA_m1 versus binder DYNA_1b1) and three (mutant target DYNA_m2 versus binder DYNA_1b1) show no localization. See fig. S21 for additional examples and methods for experimental details. (**F**) 20×20 orthogonality binding matrix determined using BLI. Biotinylated target peptide (shown in the *y*-axis) was loaded onto streptavidin biosensors and incubated with designed cognate binder and noncognate binders (labels on the *x*-axis). The heat map shows the maximum response signal for each binder-target pair normalized by the maximum response signal of the cognate at 1 uM. [Panel (E) was created with BioRender.com]

Designed binders with high affinity could be useful as enrichment reagents for a broad range of low-abundance human proteins, particularly those involved in signaling pathways. We tested this using the WASH complex as a model, a pentameric complex responsible for the nucleation of branched actin on endosomes (22, 23), and the PER complex, involved in circadian clock function. The WASH complex includes WASH (WASHC1), FAM21 (WASHC2), CCDC53 (WASHC3), SWIP (WASHC4), and Strumpellin (WASHC5) (22, 23). FAM21 contains a C-terminal disordered region of ~1000 residues in length involved in multiple protein:protein interactions (Fig. 5A). We designed three binders for a 27-amino-acid disordered window in FAM21 (Fig. 3A). Immunoprecipitation studies showed that FAM21_1b1 retrieved the entire WASH complex from cell lysate (Fig. 5A and fig. S16). Similarly, a designed binder (PER2_1b1) applied to a disordered region of PER2 (a phase separating circadian clock proteins) enriched endogenous PER2 from cell lysates in pulldown experiments (fig. S17) (24, 25). Designed binders for less well-characterized complexes involving disordered proteins could considerably enhance our understanding of the roles played by this important class of proteins.

We explored using the designed binders in affinity enrichment coupled with liquid chromatography–mass spectrometry (LC-MS) for detecting low-abundance peptides generated in proteolytic digests of the proteome (Fig. 5B), such as those from poorly folded mutant versions associated with disease, which can be very difficult to detect by mass spectrometry. We chose a 12-amino-acid tryptic peptide of a mutant form of the lysosomal cystine transporter cystinosin protein implicated in cystinosis, a lysosomal storage disease (26, 27). Binder CTN4_1b1 targeting CTN4 was coupled to magnetic beads and incubated with buffer and blood samples to which CTN4 had been added. LC-MS showed that CTN4 was captured by the binder-conjugated magnetic beads (MBs) but not the control unconjugated or bovine serum albumin (BSA)-conjugated beads. CTN4_1b1 enriched and recovered 90% of the CTN4 from both buffer and blood samples (Fig. 5B, fig. S18, and table S5), a higher recovery than that achieved with previously described helical peptide binders (8).

Mesothelin (MSLN) is a cell surface glycoprotein up-regulated in many cancers that is of considerable interest for tumor targeting (28). We investigated whether a designed binder (MSLN_1b1) made to a juxtamembrane region of MSLN could specifically bind to cells expressing the target (proteolytic cleavage in this region makes more distal regions of the extracellular domain less useful for targeting). We incubated green fluorescent protein (GFP)-MSLN_1b1 fusions with cells expressing MSLN (Human Pancreatic Adenocarcinoma, HPAC) and cell lines not expressing MSLN (Michigan Cancer Foundation-7, MCF7), along with a GFP-fusion to a control protein that does not bind MSLN (Fig. 5C). Fluorescence microscopy showed GFP localization of MSLN_1b1 at cell junctions—as expected for MSLN—on HPAC but not MCF7 control cells; no binding to HPAC cells was observed with the control binder (Fig. 5C). Thus, MSLN_1b1 specifically binds MSLN on the cell surface.

To date, no antibodies, peptides, or small molecules have been developed to inhibit dynorphin A; existing ligands instead modulate KOR signaling by engaging the deep binding pocket of the receptor (18, 20). To explore the potential of our binders to block KOR signaling mediated by dynorphin A (fig. S19), we performed an in vitro cyclic adenosine monophosphate (cAMP) assay using mammalian cells stably expressing the human KOR. The binder DYNA_2b2 inhibited dynorphin A–dependent KOR signaling with an IC_50_ of 50 nM (Fig. 5D; the IC50 is higher than the K_d_ as a result of competition with KOR binding). As noted above, to increase specificity we excluded the N-terminal YGGF sequence during design to distinguish between dynorphin A and B; this region is critical for opioid receptor activation (29) (fig. S20), and extension to include the YGGF motif would likely increase potency.

## Binder orthogonality

We investigated the specificity of interactions between the designed binders in cells using a mitochondria colocalization assay (7). We expressed six of the designed binders fused to sfGFP (30), six targeted disordered sequences fused to mCherry, and a mitochondrial outer membrane targeting sequence. Each designed binder was expressed with each target one at a time, and binding was evaluated by localization of GFP fluorescence to the mitochondria. We observed localization of the GFP to the mitochondria for each on-target pair (designed binder with its intended target), but not for any off-target pairs (Fig. 5E and fig. S21), indicating that the designed binders function in cells. As further in-cell off-target controls, two sequence homologs of DYNA were also tested and found not to colocalize with the DYNA binder at all (see illustrations in Fig. 5E and experimental methods).

We investigated the specificity of the designed binders for 16 native targets and four representative synthetic targets with the most distinct target amino acid sequences. The affinities of these designs for their targets are all tighter than 100 nM. We measured the binding affinity for each design against all 20 targets using BLI and observed little cross reactivity at concentrations up to 1 uM. Within the set of disordered targets considered here, each design thus only binds tightly to the target it was designed to bind (Fig. 5F and fig. S22).

## Discussion

We demonstrate that the conformational heterogeneity of IDPs and proteins can be exploited to make the binder design problem easier than that for traditional stable folded targets. For each target, we sampled a wide variety of conformations and identified those compatible with high-affinity binding. This contrasts with folded targets, whose fixed structures may admit few optimal binding solutions. Many of our designs induce the disordered targets to adopt structures different from those populated in solution or present in previously solved native complexes. Induced fit is a general feature of disordered protein binding interactions in nature (31–33), and by taking advantage of induced fit, our approach of threading through a diverse extended scaffold set followed by diffusion-based diversification and refinement enables robust computational design of binders to a wide range of disordered sequences, including highly polar, challenging sequences.

The design method, which we call “logos”, has high computational efficiency and a high experimental success rate (22 designs were tested on average for each case described here ), and should be broadly useful for making binders to arbitrary disordered targets of interest (fig. S23 provides a step-by-step example of a design campaign and fig. S24 shows structure and sequence comparisons of the designed binders exhibiting commonality in overall structures and diversity in binding interfaces). Beyond the examples presented here, the logos method has been used to generate binders the unstructured termini of three polar isoforms of Ras (Hras, Kras-A, and Kras-B), which have high specificity in cells (34). More generally, the combination of physically based design approaches for generating a set of privileged starting scaffolds (such as the extended peptide binding protein structures we started with) and deep learning generative methods for introducing diversity and refinement could be useful for many future challenging design problems.

There are many applications for designed IDR binding proteins. Cancer and disease-related cell receptors such as MSLN (35), oncofusion proteins associated with childhood cancer such as EWS/FLI for ewing sarcoma, EML4-ALK for lung cancer (36), and CSP for malaria (16) can be targeted through their unstructured regions for delivery or degradation, as can the many transcription factors, epigenetic regulation, and viral-host protein-protein interactions involve intrinsically disordered regions (37). Disordered targets ranging from neuropeptides to noncanonical open reading frames (38, 39), such as GREP1 (39) implicated in breast cancer, could become accessible for imaging and sensing. The enhanced detection of the cystinosin tryptic peptide CTN4 suggests the potential for low-cost proteomics platforms based on arrays of peptide binding proteins. For several of these applications, it will be important to extend the characterization of interaction specificity from beyond the sets of targets considered here to the entire proteome; one advantage of targeting long IDRs is that affinity and specificity can in principle be increased with bispecific constructs targeting two distinct epitopes within the same target.

There are several potential directions for extending our design approach: First, it should be possible to design binders sensitive to post translationally modifications such as phosphorylation on tyrosine and serine. Second, as catalytic site design methods improve, it should be possible to incorporate proteolytic or covalent modification sites into the designs; the extended conformation of the peptide bond and the pocket-by-pocket sidechain recognition are advantageous properties for protease substrates. The binding pockets and conformations of peptides in most natural proteases resemble our designs, but completely redesigning natural enzyme specificity has proven challenging—instead, designing binders to the target of interest as described here and then incorporating catalytic sites could provide a more general customizable approach.

## Supplementary Material

SI

MDAR Reproducibility checklist

## Data Availability

All data are available in the main text or as supplementary materials. Design scripts and models are archived at Zenodo (*40*). Crystallographic datasets have been deposited in the Protein Data Bank (PDB) with accession codes 9cce and 9ccf. NMR data and resonance assignments have been deposited in BioMagResDB (BMRB) with accession codes 52752, 52755, and 52756.
