# Supplementary material for: Design of intrinsically disordered region binding proteins
Source: Science. Author manuscript; Available in PMC 2026 Feb 28. (PMC12949689; doi:10.1126/science.adr8063)
Supplement: SI [file NIHMS2131716-supplement-SI.pdf]

Science
AAAS

Supplementary Materials for

Design of intrinsically disordered region binding proteins

Kejia Wu *et al*.

Corresponding authors: Kejia Wu, kejiawu@uw.edu; David Baker, dabaker@uw.edu

Science **389**, eadr8063 (2025)
DOI: 10.1126/science.adr8063

The PDF file includes:

Materials and Methods
Supplementary Text
Figs. S1 to S24
Tables S1 to S5
References

Other Supplementary Material for this manuscript includes the following:

MDAR Reproducibility Checklist

Supplemental Figs. S1-S22

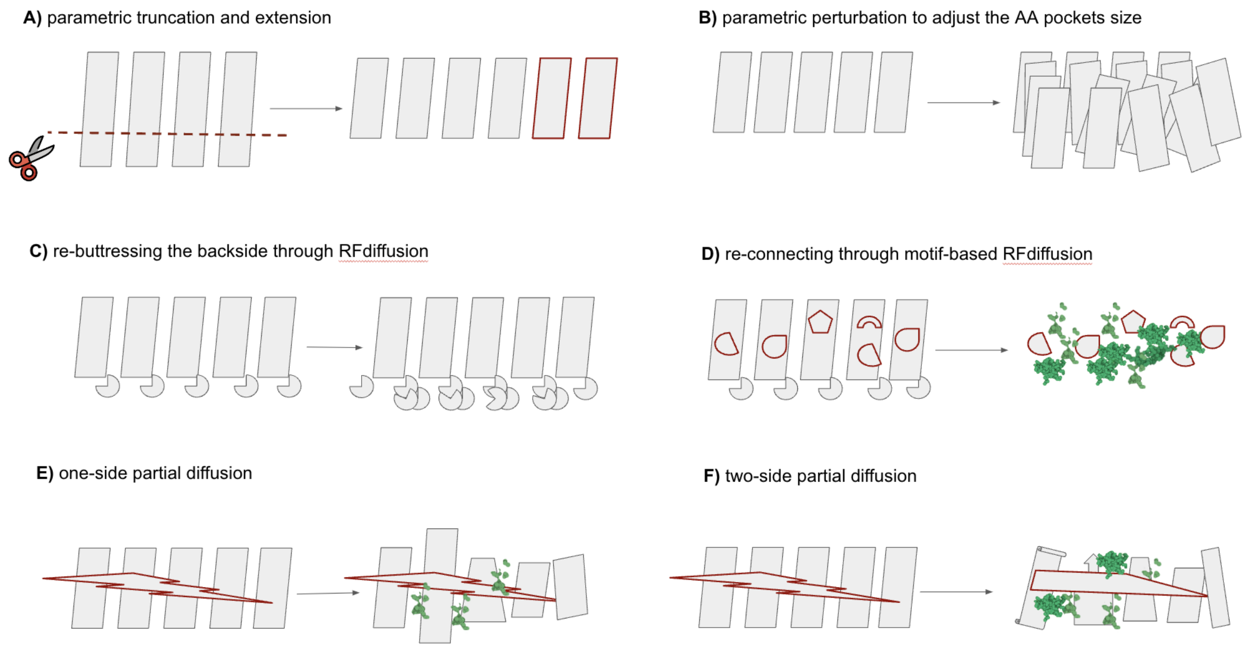

Fig. S1: Template backbone optimization approaches. Multiple approaches were applied to generate the library templates during pocket design. **(A)** Original four-repeat proteins with long helices were truncated by helical length and extended by repeating geometry parametrically to generate five- and six-repeat proteins with short helices for a suitable peptide binding interface. **(B)** Parametric perturbations between the repeat-to-repeat transition were applied both symmetrically and asymmetrically to adjust the pocket size. **(C)** Each of the original repeat proteins was made of two-helix bundles. To re-buttress and re-pack the core of the protein, all the helices interacting with the peptide were fixed, while the back helices were masked, denoise, and regenerated with RFdiffusion with the context of the target peptide. **(D)** Similar to c, per repeat, only the four to nine interacting residues (around the bidentate hydrogen bond donor) to the peptide were fixed. The rest of the protein was masked, denoised, and regenerated with RFdiffusion in the context of the target peptide. **(E)** One-side (chain A, which is the designed protein) partial diffusion was conducted as published with PT ranging from [10, 12, 15, 18]. **(F)** Two-side (chain A and chain B, which are the designed protein and the peptide target) partial diffusion was conducted with PT ranging from [12, 15, 18, 22].

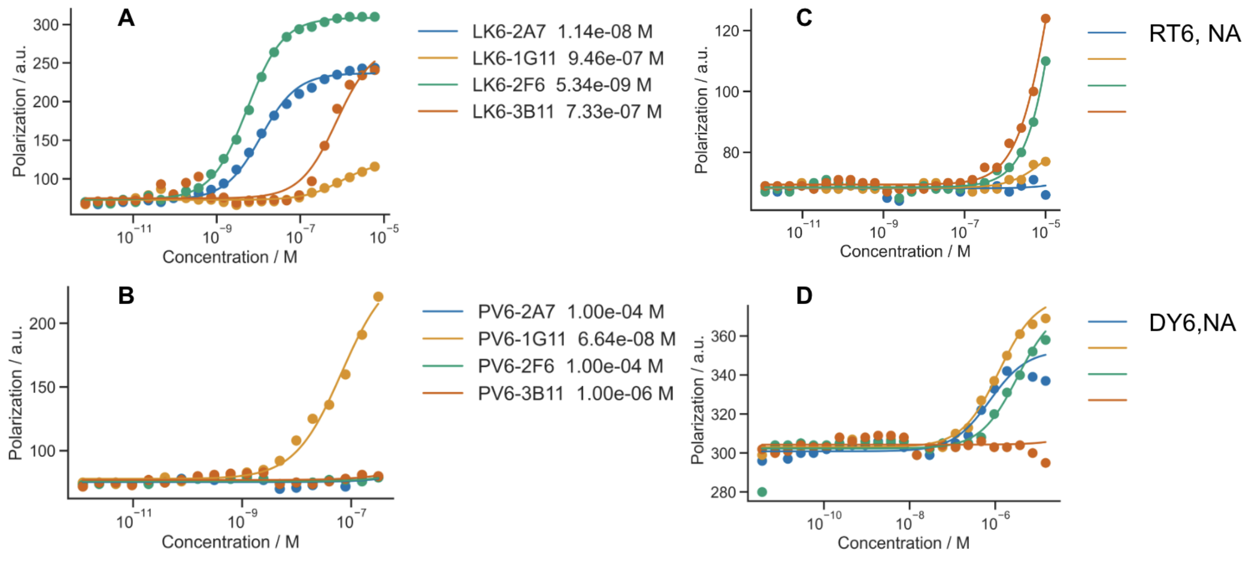

Fig. S2: Initial binding characterizations of the round one di-peptide repeat binders by fluorescence polarization (FP). FP characterization of the first-round four-repeat designed binders titrating against TAMRA-labeled six-repeat di-peptides: **(A)** (LK)x6; **(B)** (PV)x6; **(C)** (RT)x6; **(D)** (DY)x6. TAMRA-peptides were maintained at 1 nM concentration, while designed binders started from 40 uM (DY), 10 uM (LK, RT), and 1 uM (PV) with two-folded titration.

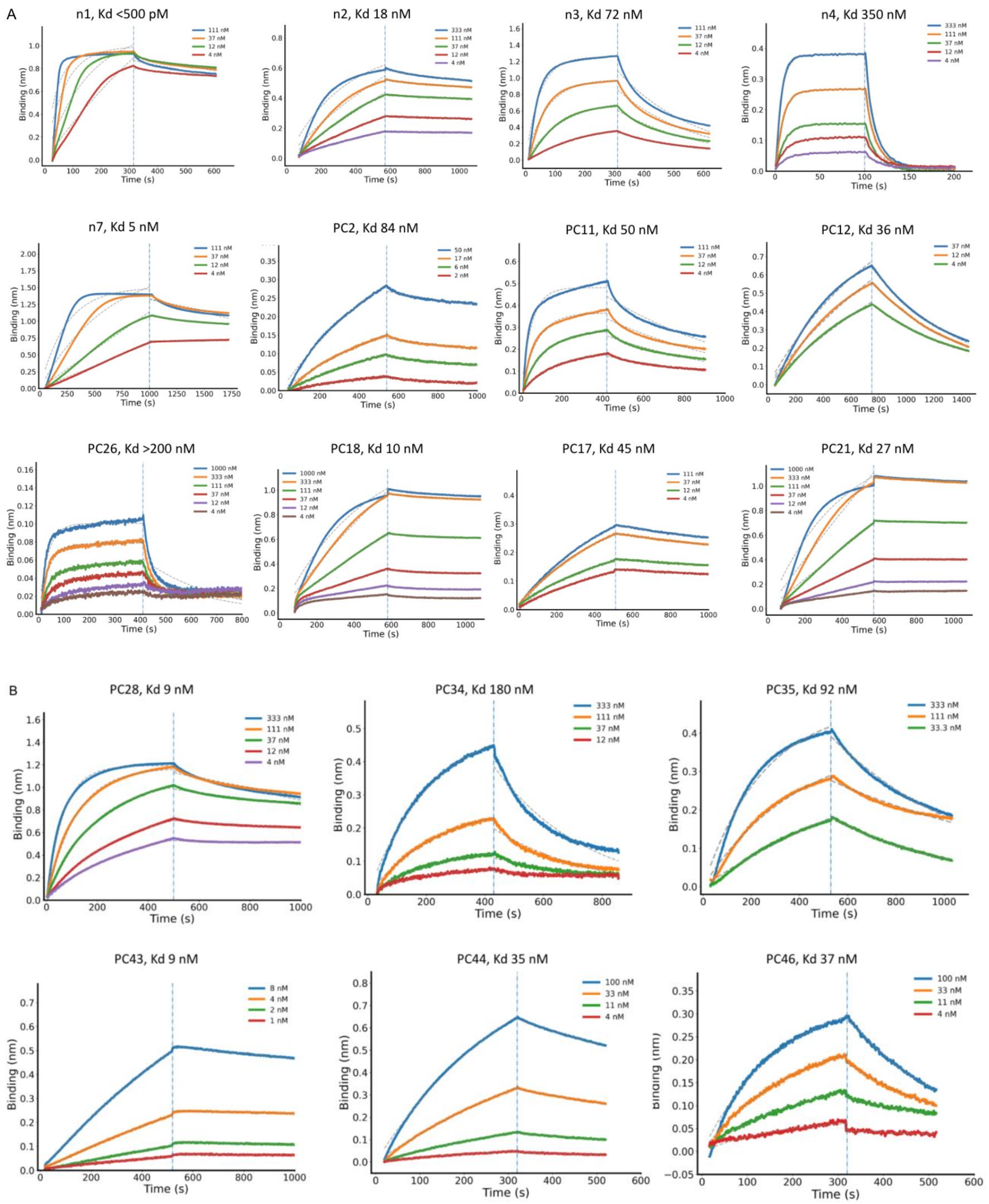

Fig. S3: Octet, BLI measurements toward all 18 synthetic peptide ligands. Three-fold or two-fold serial dilutions were tested for each binder, and the full tested concentrations are labeled in each plot. Curve fits were overlaid on the traces with grey

dotted lines. In panel **(A)**, i.e., binding to di-peptide repeats and chimeric targets, the biotinylated targets were loaded onto the streptavidin (SA) biosensors, and incubated with designed binders in solution to measure association and dissociation. In panel **(B)**, i.e. binding to English words, the biotinylated designed binders were loaded onto the streptavidin (SA) biosensors, and incubated with sfGFP-targets in solution to measure association and dissociation except in the case of PC34.

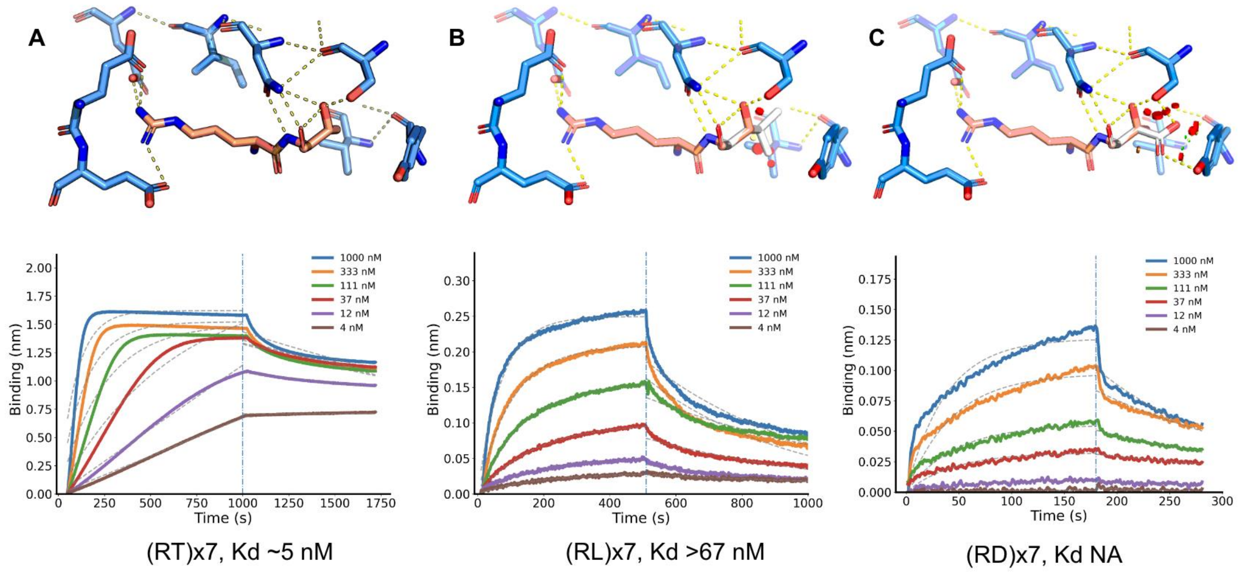

Fig. S4: Repeat di-peptide (RT) binder showed selectivity between threonine, aspartate, and leucine by BLI. BLI characterization for the designed (RT) binder titrating against three closely related peptide targets, **(A)** (RT)x7; **(B)** (RL)x7; **(C)** (RD)x7. Three-fold serial dilutions were tested for each binder, and the full tested concentration is labeled. The biotinylated targets were loaded onto the streptavidin (SA) biosensors, and incubated with designed binders in solution to measure association and dissociation.

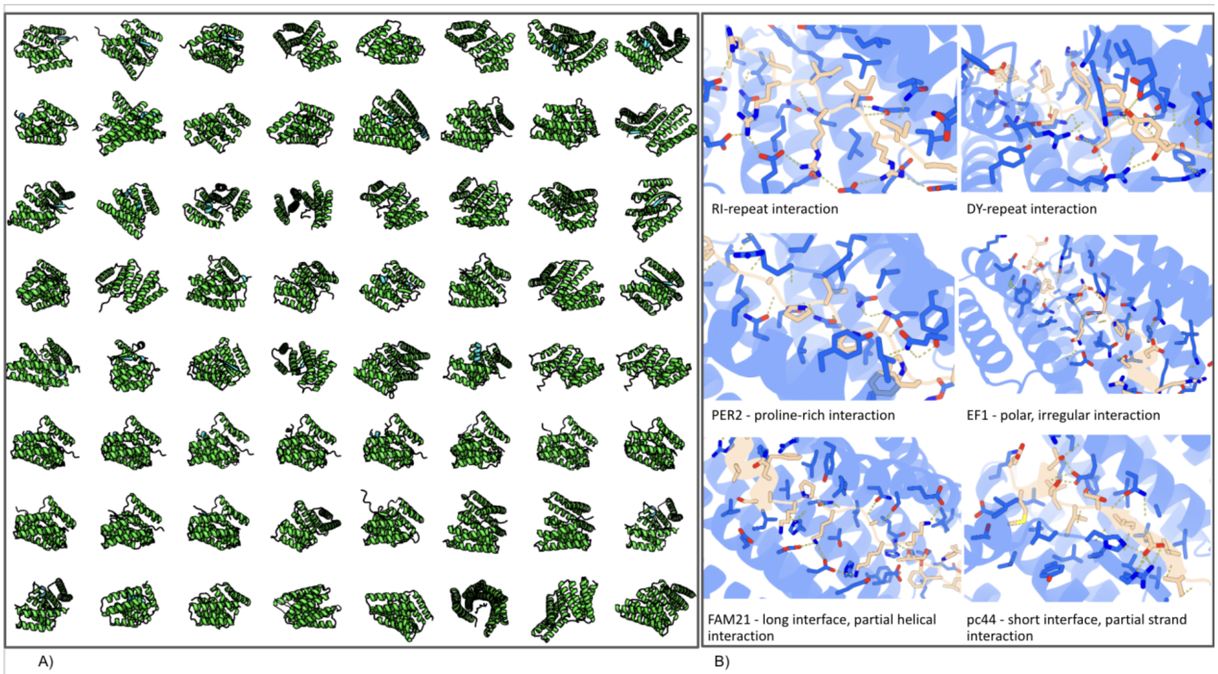

Fig. S5: Gallery of the designed binders out of logos pipeline with highly converged helical bundle based geometries and locally diverse assembled pockets into individual designs. (A) A collection of experimentally tested binding complexes against 39 targets summarized in this work. As shown in zoom-out models, the majority of the designs are largely helical bundles. **(B)** For different types of disordered target sequence properties, the local geometry and pocket arrangement vary to assemble with the most complementary interface interactions.

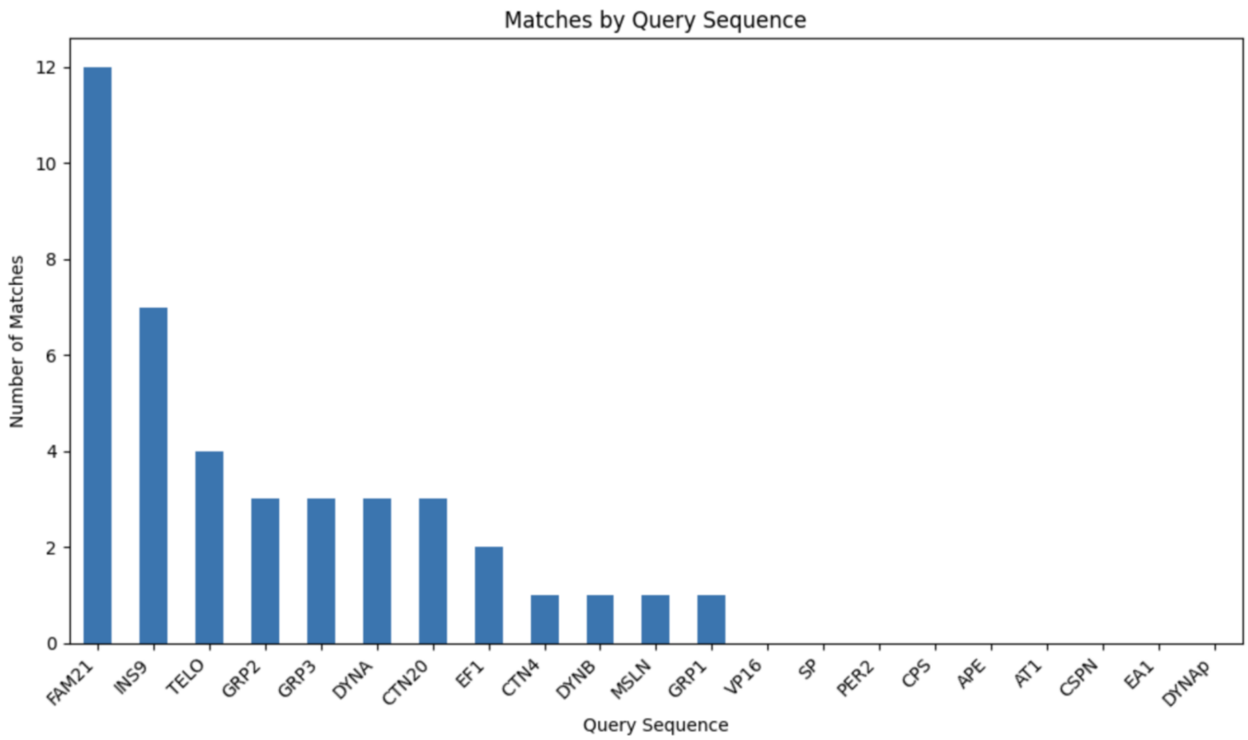

Fig. S6: 21 native targets uniqueness analysis in human proteome. BLAST search was done of the 21 native targets in this work against the human proteome database from Uniprot. Percent identity more than 70% with e-value no greater than 0.01 were identified as matches, meaning 70% of the query sequence was aligned with confidence.

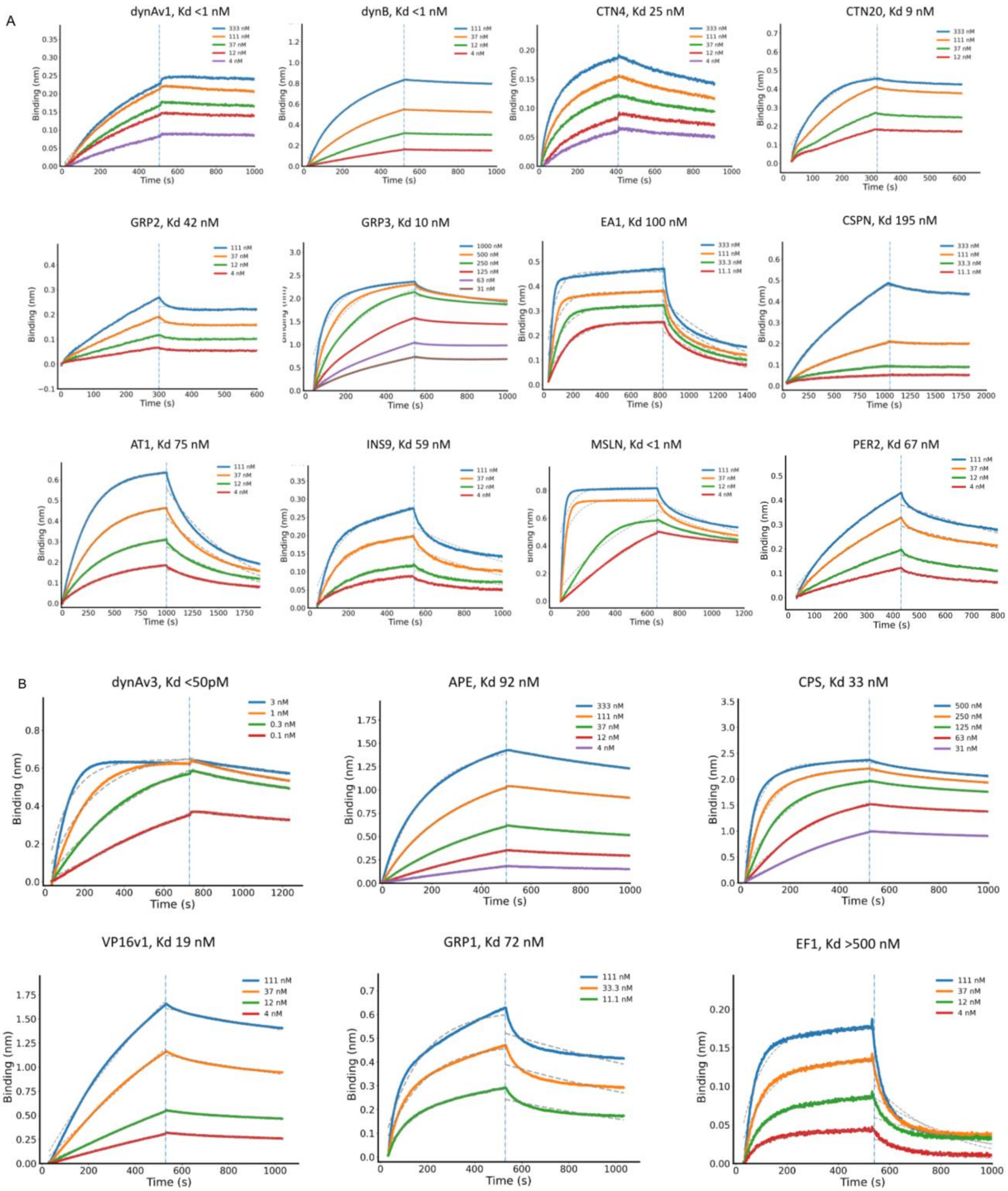

A
dynAv1, Kd <1 nM
333 nM
111 nM
37 nM
12 nM
4 nM
dynB, Kd <1 nM
111 nM
37 nM
12 nM
4 nM
CTN4, Kd 25 nM
333 nM
111 nM
37 nM
12 nM
4 nM
CTN20, Kd 9 nM
333 nM
111 nM
37 nM
12 nM
GRP2, Kd 42 nM
111 nM
37 nM
12 nM
4 nM
GRP3, Kd 10 nM
1000 nM
500 nM
250 nM
125 nM
63 nM
31 nM
EA1, Kd 100 nM
333 nM
111 nM
33.3 nM
11.1 nM
CSPN, Kd 195 nM
333 nM
111 nM
33.3 nM
11.1 nM
AT1, Kd 75 nM
111 nM
37 nM
12 nM
4 nM
INS9, Kd 59 nM
111 nM
37 nM
12 nM
4 nM
MSLN, Kd <1 nM
111 nM
37 nM
12 nM
4 nM
PER2, Kd 67 nM
111 nM
37 nM
12 nM
4 nM
Binding (nm)
Time (s)
B
dynAv3, Kd <50pM
3 nM
1 nM
0.3 nM
0.1 nM
APE, Kd 92 nM
333 nM
111 nM
37 nM
12 nM
4 nM
CPS, Kd 33 nM
500 nM
250 nM
125 nM
63 nM
31 nM
VP16v1, Kd 19 nM
111 nM
37 nM
12 nM
4 nM
GRP1, Kd 72 nM
111 nM
33.3 nM
11.1 nM
EF1, Kd >500 nM
111 nM
37 nM
12 nM
4 nM
Binding (nm)
Time (s)

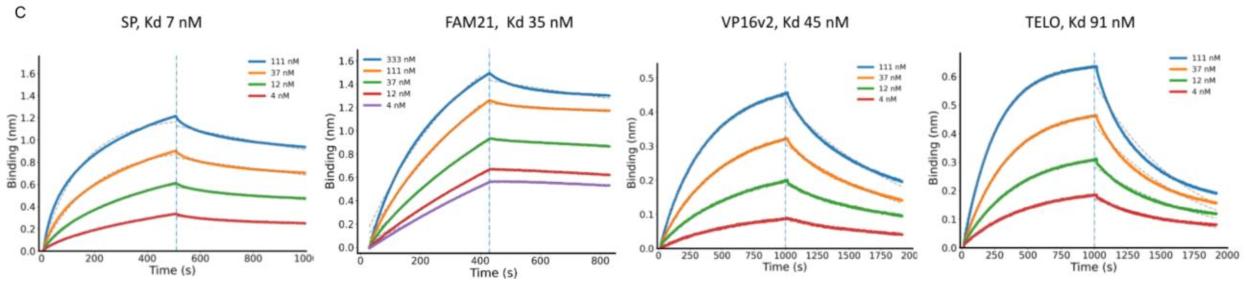

Fig. S7: Octet, BLI measurements toward all 21 native disordered targets in this work. Three-fold or two-fold serial dilutions were tested for each binder, and the full tested concentrations are labeled in each plot. Curve fits were overlaid on the traces with grey dotted lines. Panel (**A)** collects binding to native targets in diverse extended random coil conformations, panel **(B)** binding to native targets in partial strand conformations, while panel **c,** binding to native targets in partial helical conformations. In most cases but EA1, AT1, INS9, GRP1, the biotinylated targets were loaded onto the streptavidin (SA) biosensors, and incubated with designed binders in solution to measure association and dissociation. In the cases of EA1, AT1, INS9, GRP1, the biotinylated designed binders were loaded onto the streptavidin (SA) biosensors, and incubated with sfGFP-targets in solution to measure association and dissociation.

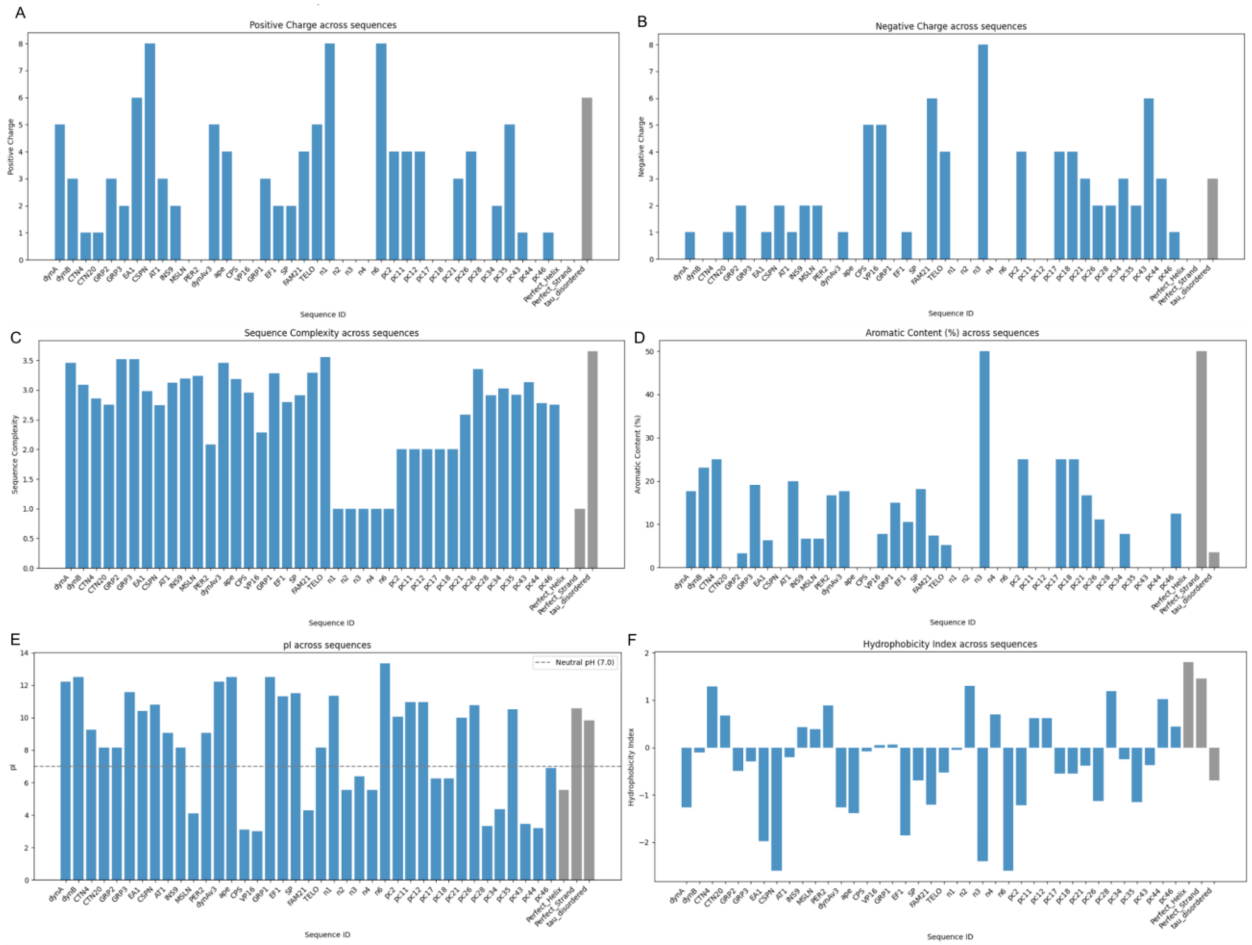

Fig. S8: Summary of sequence properties of all 39 targets in comparison to the perfect helix (poly(A)), perfect strand (poly(VK)), and tau protein as an example of well-known IDP as references. a, Positive net charge distribution of all 42 targets. **b,** Negative net charge distribution of all 42 targets. **c,** Sequence complexity of all 42 targets. **d,** Aromatic content (%) of all 42 targets. **e,** The isoelectric point (pI values) of all 42 targets. **f,** Hydrophobicity index of all 42 targets.

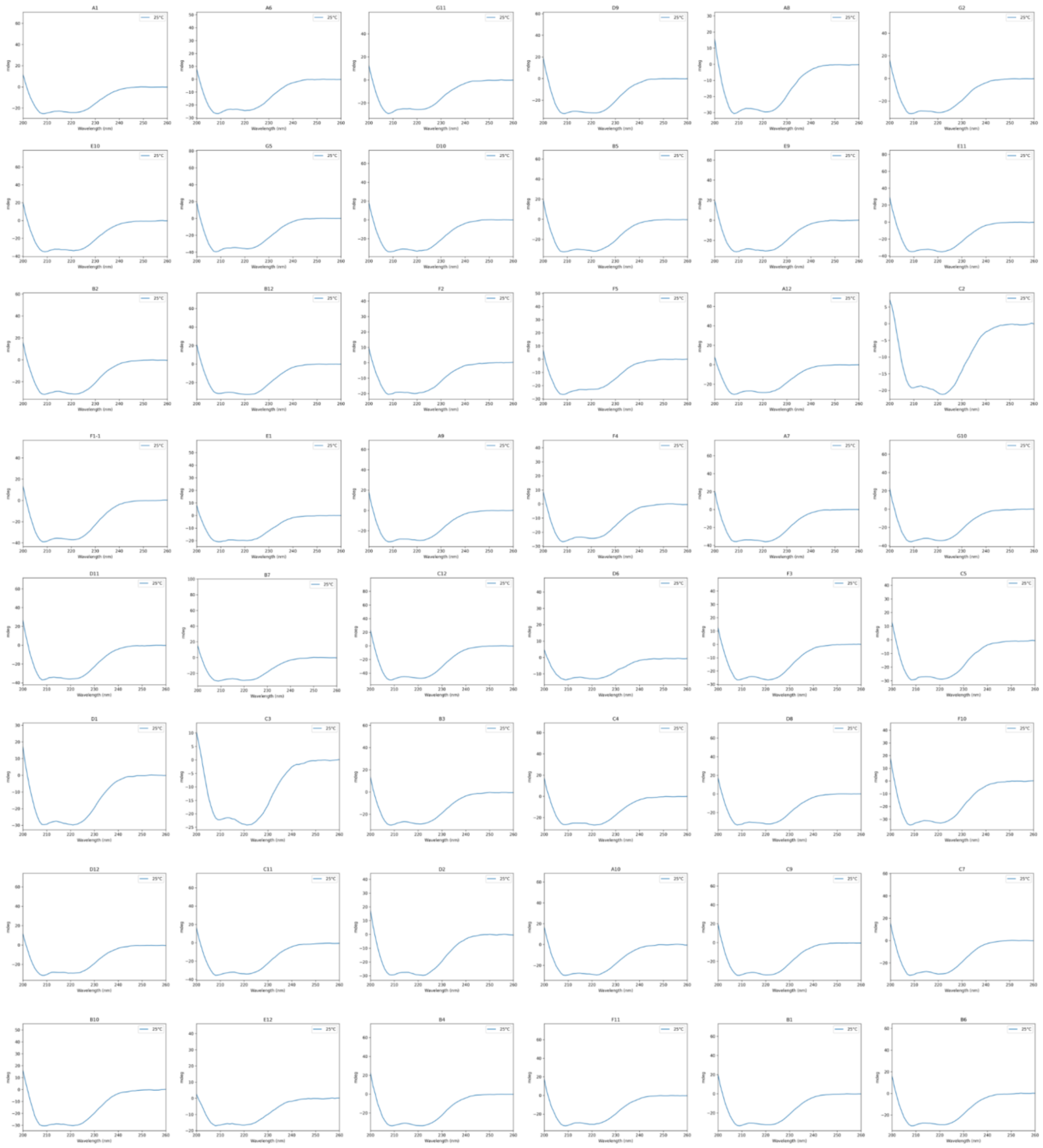

Fig. S9: CD plots for all 48 designed binders presented in this work. Circular dichroism spectra were measured with an AVIV Model 420 DC or Jasco J-1500 circular dichroism spectrometer. Samples were 0.2 mg ml−1 in TBS (25 mM Tris pH 8.0 and 150 mM NaCl), and a 1-mm path-length cuvette was used. The circular dichroism signal

was converted to mean residue ellipticity by dividing the raw spectra by $N \times C \times L \times 10$, in which N is the number of residues, C is the concentration of protein and L is the path length (0.1 cm).

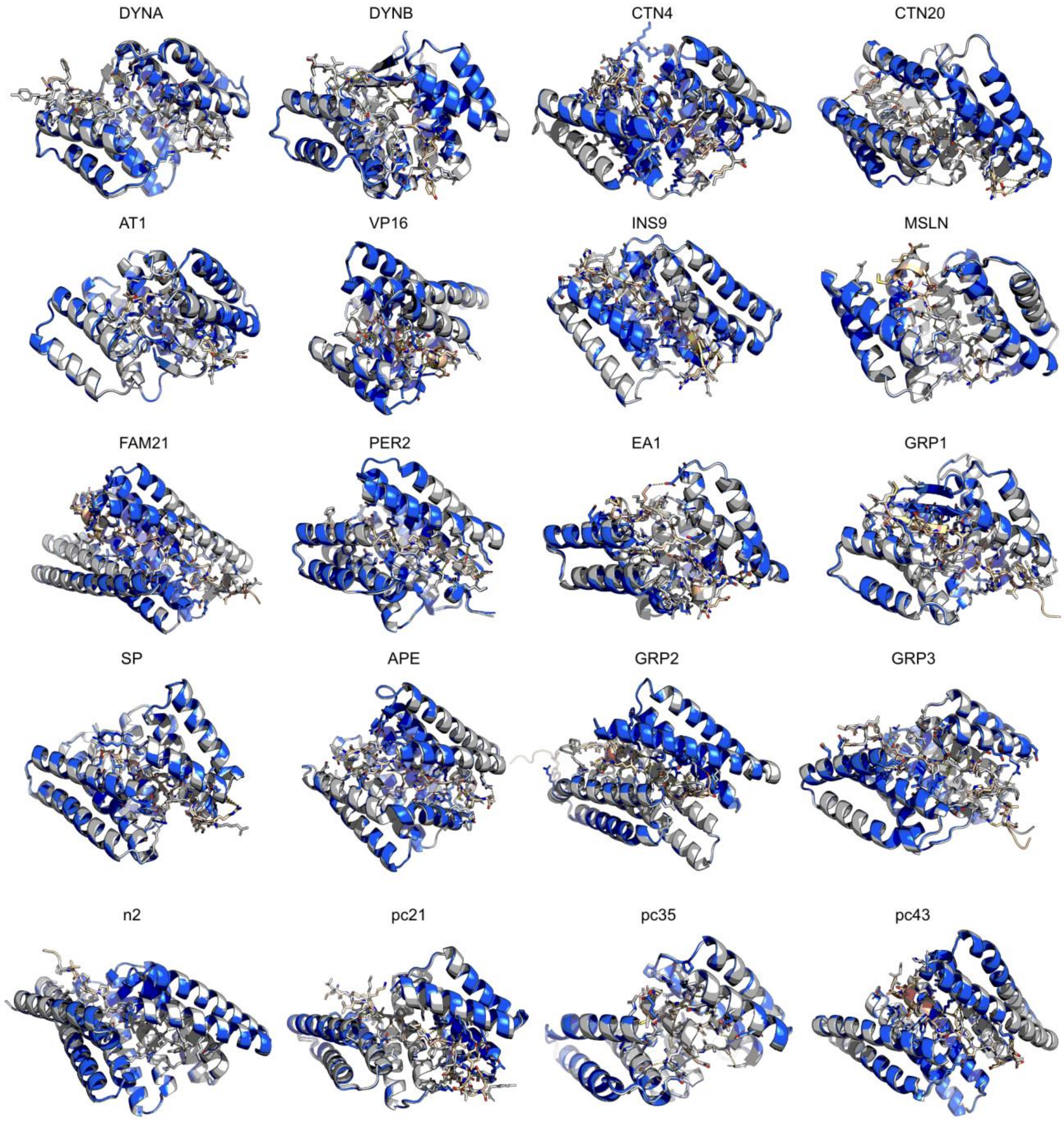

Figure S10. Side-by-side comparison of design models and AF2 predictions of 20 representative designs in Fig. 5f. Designed models (blue and wheat) are aligned with AF2 predictions (grey), with interfaces shown in sticks.

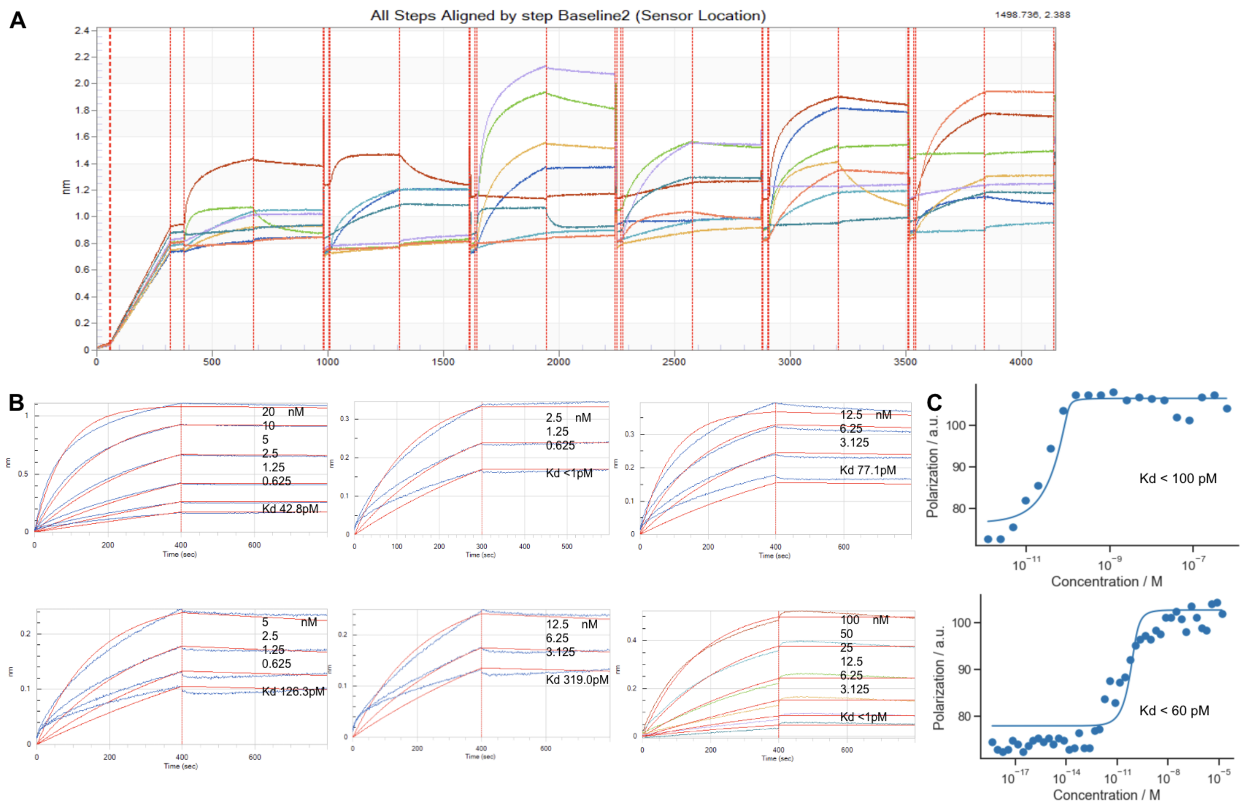

Fig. S11: Tight binding of designed dynorphin A binders. In the second design round, the optimized dynorphin A binders showed **(A)** exceptional screening success rate by Octet, BLI with biotinylated target loaded on SA biosensors to 0.7-0.9 nm, while screening against all 48 designs at 5 nM binder concentration. After equilibration, each individual trace shows an independent single-concentration association and dissociation, followed by tip regeneration in HCl pH 1.0. **(B)** ultra-tight binding for six binders 'titration by Octet, BLI (tested concentrations and kinetic Kd fittings were labeled under each plot), estimated Kd ranging from <1pM ~ 300pM, and **(C)** ultra-tight binding for two binders 'thermodynamic fitting by fluorescence polarization (FP) with 100pM TAMRA labeled peptide, estimated as Kd <100 pM, Kd <60 pM (Kd cannot be accurately measured below the concentration of peptide used in the FP assay).

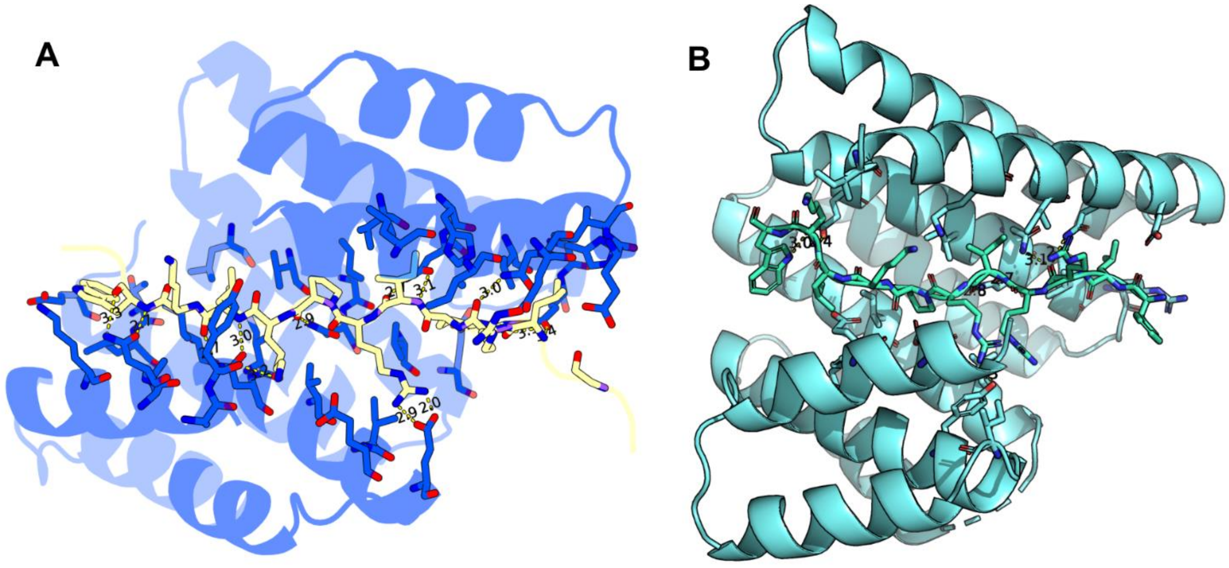

Fig. S12: Comparison of the structural binding modes of a, initial design DYNA_1b1 to b, two-sided partial diffusion refined design DYNA_1b7. During partial diffusion, the binding mode was further changed and diversified on both the binder and the peptide arrangement (in terms of how the peptide docked into the binder) due to partial diffusion without fixing motifs. This partially diffused design showed a slightly worse binding affinity (Kd = 7 nM) compared to the original design (Kd < 1 nM), which inspired us to invent “motif diffusion,” i.e., all the key interaction motifs on a binder are kept during diffusion re-connecting them into new, better proteins.

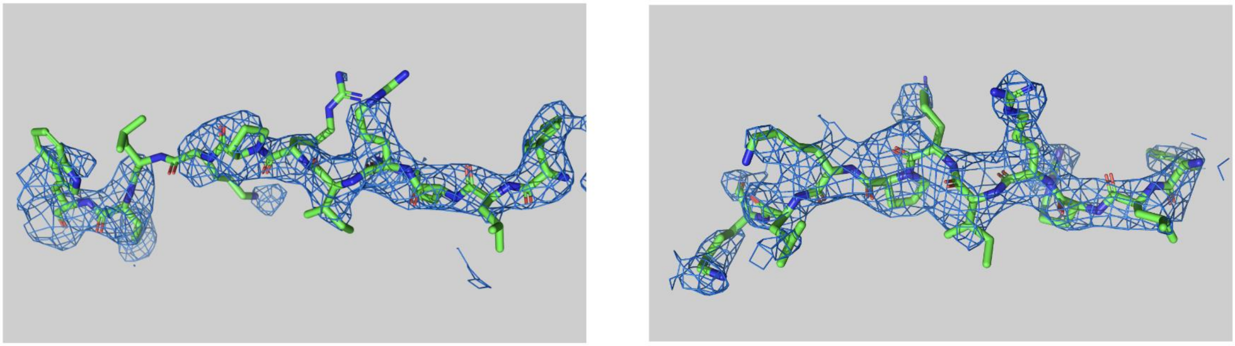

Fig. S13: Density maps of peptide DYNA in the xtal structure. Both maps were converted from MTZ map coefficients of the dimer structures respectively.

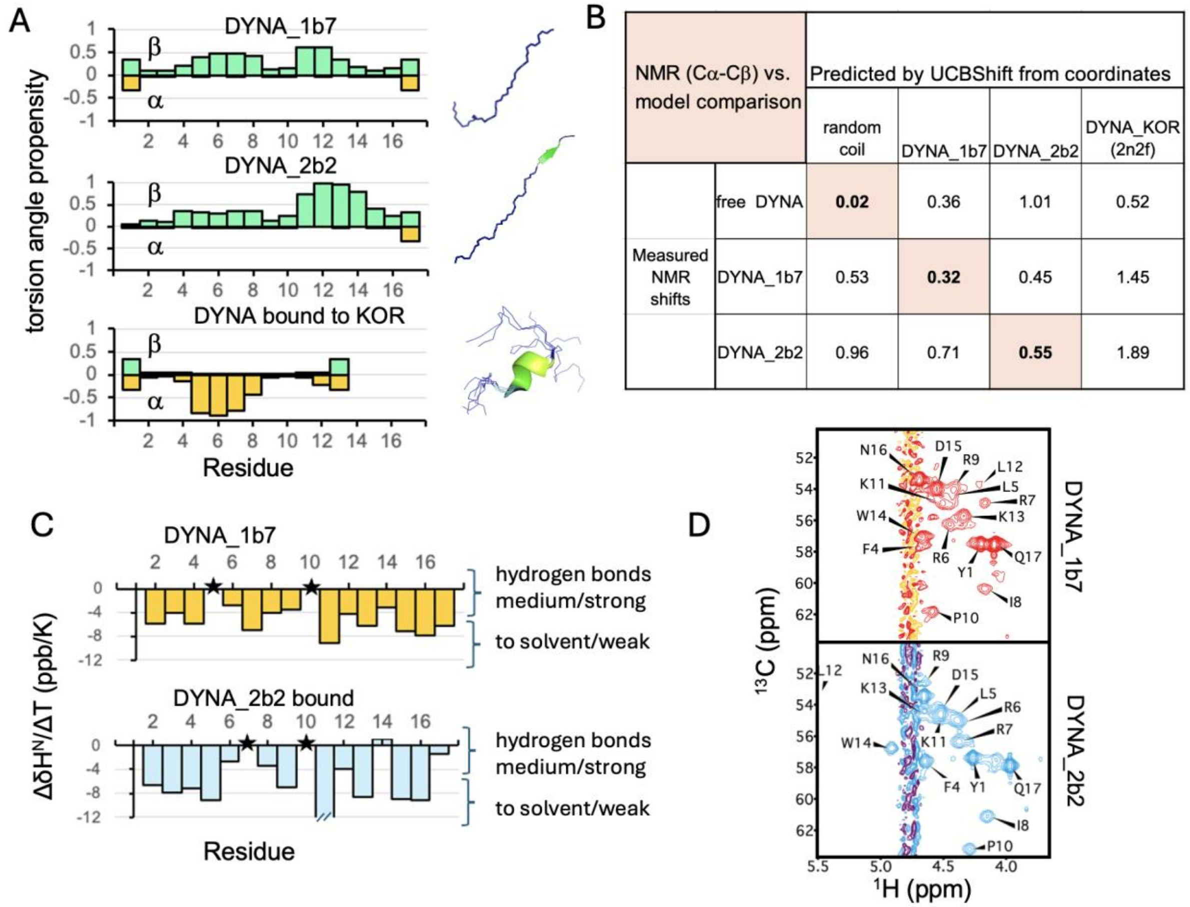

NMR (Cα-Cβ) vs. model comparison		Predicted by UCBShift from coordinates			
		random coil	DYNA_1b7	DYNA_2b2	DYNA_KOR (2n2f)
	free DYNA	**0.02**	0.36	1.01	0.52
Measured NMR shifts	DYNA_1b7	0.53	**0.32**	0.45	1.45
	DYNA_2b2	0.96	0.71	**0.55**	1.89

Fig. S14: NMR analysis on dynorphin A complexes. Dynorphin A binds as extended and partial beta strands as designed. **(A)** NMR chemical shift-based analysis using Talos-N of isotope-enriched dynorphins bound to DYNA_1b7 and DYNA_2b2 distinguish helical (α) from extended (β) conformations for each residue along its sequence. Its binding mode is different from the short helix formed when bound to the native human KOR (PDB: 2n2f with chemical shifts predicted by UCBShift). **(B)** Validation of design DYNA_2b2 by comparison of experimental ^{13}Cα and ^{13}Cβ chemical shifts with back-calculated chemical shifts (units in ppm; scaled RMSE analysis) shows that each set of measured NMR chemical shift values agrees best with those predicted from the corresponding atomic coordinates (DYNA_1b7 (PDB: 9cce), DYNA_2b2 (PDB: 2n2f)) by UCBShift(*1*), rather than with chemical shifts predicted from the other models. **(C)** Amide temperature coefficients indicating hydrogen-bond donor amide protons; the backbone amide of W14 of DYNA_2b2 has the strongest hydrogen bond, corresponding to the bidentate hydrogen bonds between W14 backbone atoms and the polar Asn180 side chain of the binder. On either side of W14, there are predicted dynA to binder backbone-backbone hydrogen bonds for K13 and D15 to receptor S5 and K3, in two short antiparallel beta strands; two between K13 NH/CO and receptor S5 CO/NH, and two between D15 NH/CO and receptor K3 CO/NH forming the hydrogen bonded network. The star symbol indicates amide sites not accessible to measurement. **(D)** ^{1}H-^{13}C HSQC spectra of Hα – Cα region. The downfield ^{1}Hα shifts of L12, K13, and W14 in 2b2 versus 1b7 indicate enhanced binding interactions.

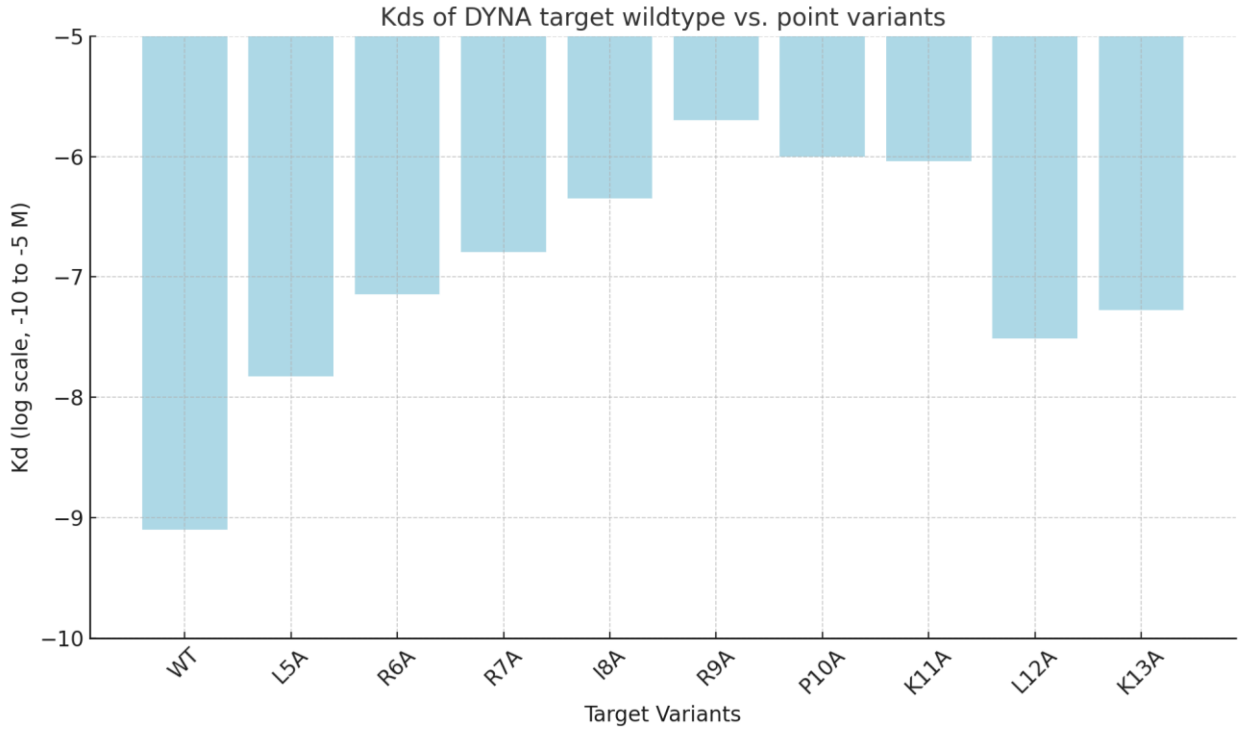

Fig. S15: Binding comparisons between the designed wildtype DYNA pair, DYNA_2b1-DYNA and DYNA_2b1-DYNA_variants. Designed binder DYNA_1b1 were fused to smBiT, while WT target DYNA and its nine Alanine point mutants on the main interacting interface were fused to lgBiT. Kds were measured with nanoBiT assay, when [lgBiT] == 1nM, while [smBiT] == [1000 nM, 333 nM, 111 nM, 33.3 nM, 11.1 nM].

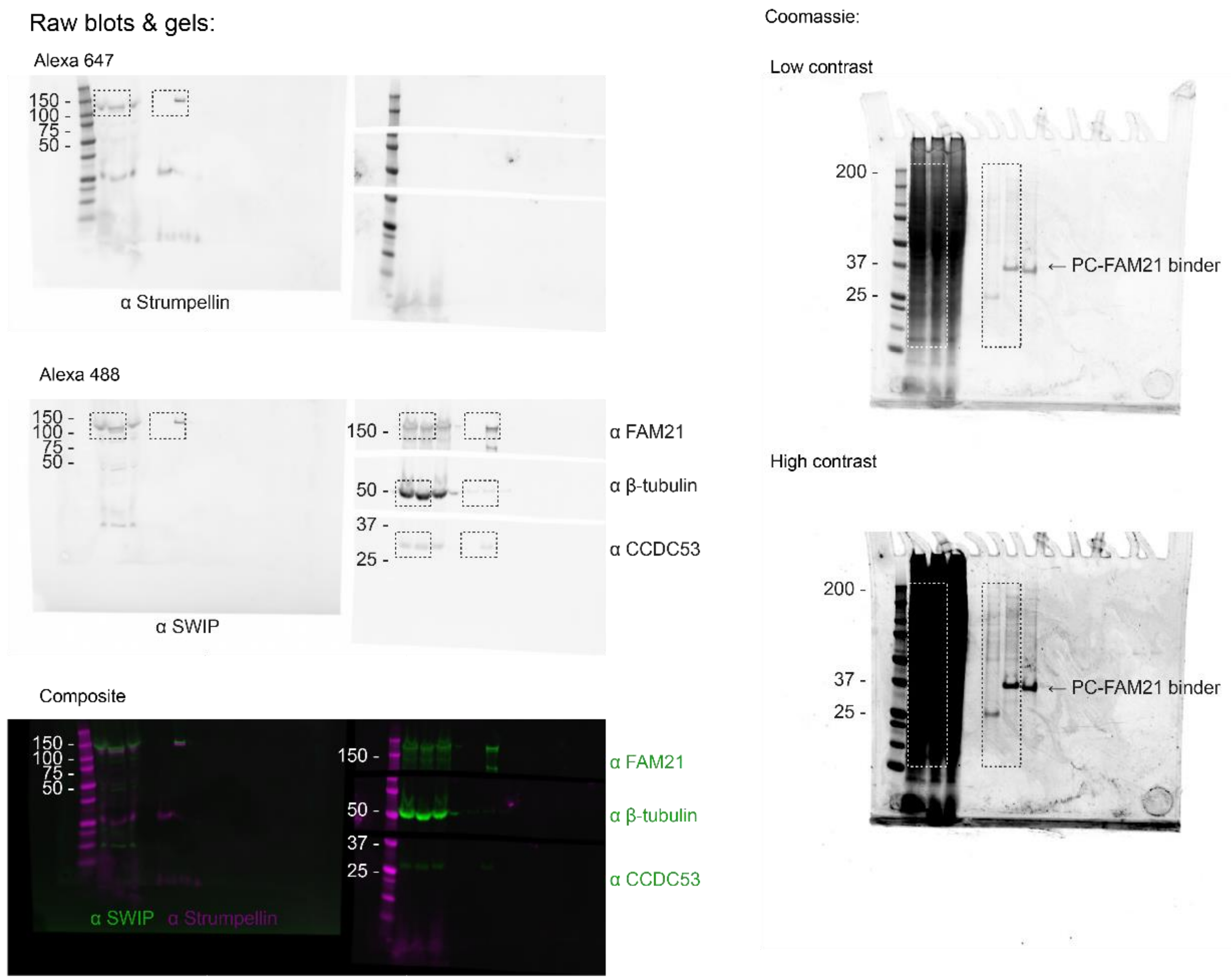

Fig. S16: Raw data for Western Blots of FAM21 complexes in Figure 5A. β-tubulin was blotted as loading control and is not immunoprecipitated by PC-FAM21. To perform immunoprecipitation with the designed binders, 20 µg of designed PC tagged FAM21 binders were added to Protein C (PC) resin and incubated with HEK293 lysate. Immunoprecipitated proteins were assessed by Coomassie stain and Western blotting against indicated antibodies. PC-FAM21_1b1 was sufficient to immunoprecipitate FAM21 and other WASH complex subunits (SWIP, CCDC53 and Strumpellin), from the cell lysate. β-tubulin was blotted for as a loading control and is not immunoprecipitated by PC-FAM21. Both lower contrast and higher contrast images of the Coomassie gel and uncropped blots shown here on the right, in supplement to Fig. 5a, with representative blot from 3 independent experiments.

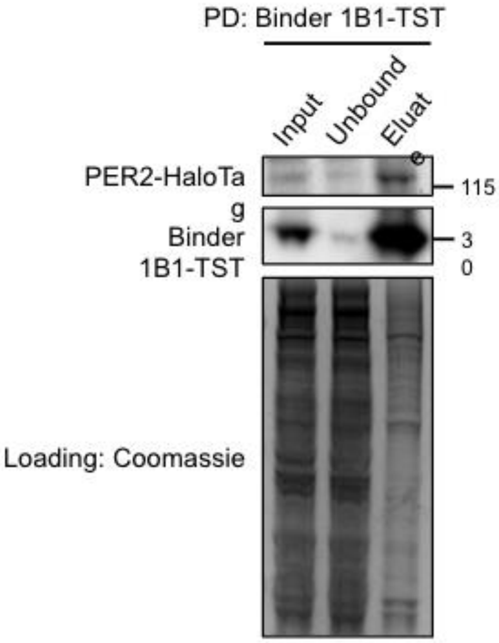

Fig. S17: Western blots of PER2 enrichment. Binder PER2_1b1 interacts with endogenous PER2. Binder 1b1 was constitutively expressed in U2OS cells and pulled down via a C-terminal Twin-Strep tag (TST) under native conditions. Endogenously tagged PER2 (with a HaloTag) was successfully enriched using binder 1b1.

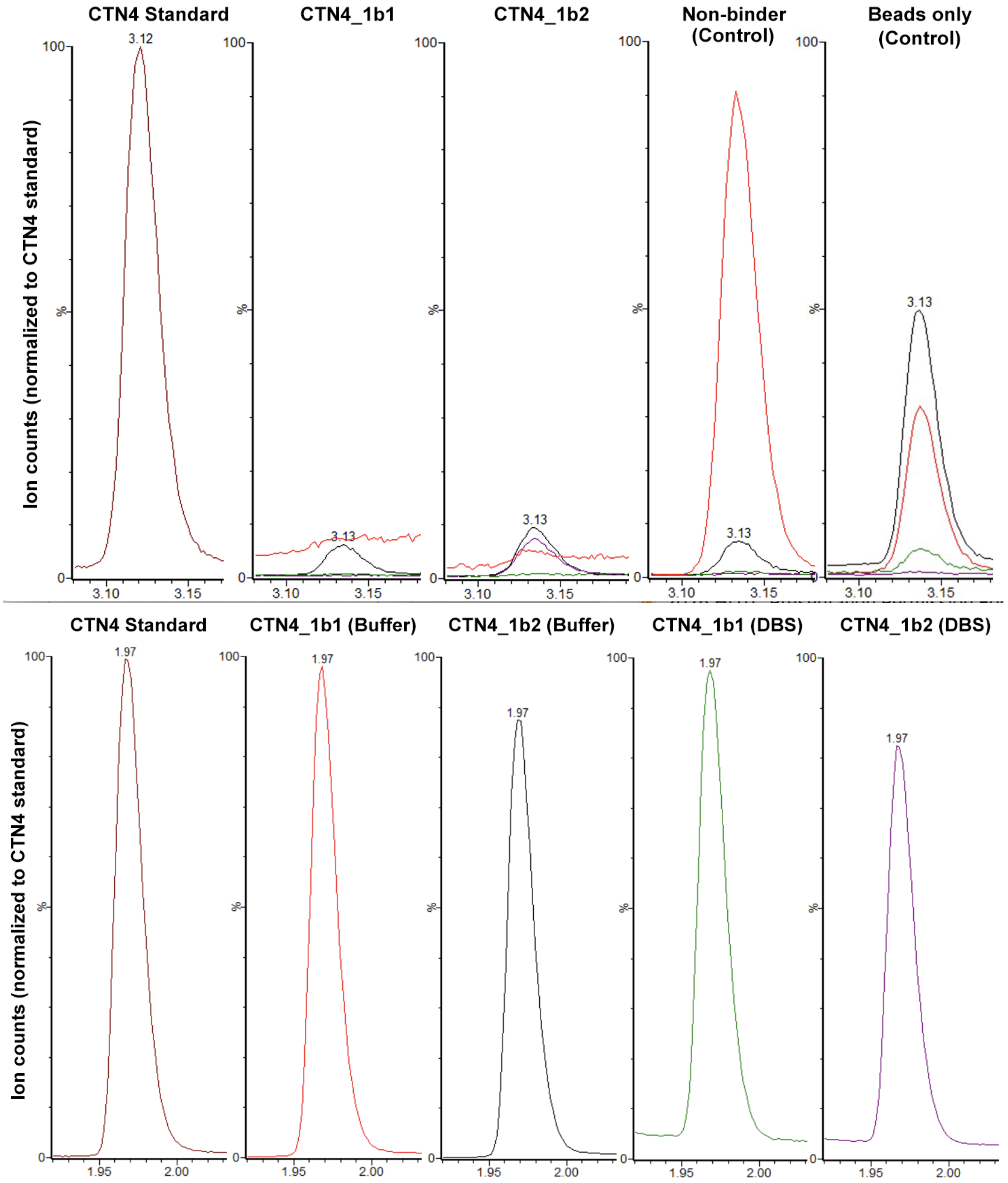

Fig. S18: LC-MS chromatograms for CTN4 binders. Upper, LC-MS chromatograms for wash-and-loss experiment in spiked buffer. From left to right: CTN4 standard (representing 100% loss), CTN4_1b1, CTN4_1b2, random off-target binder (negative control), non-functionalized Dynabeads (negative control). Color of different traces

represent peptide standard (brown), supernatant (red), wash #1 (black), wash #2 (green), and wash #3 (purple). Lower, LC-MS chromatograms for recovery experiment in spiked buffer and spiked DBS extract. From left to right: CTN4 standard (representing 100% recovery), CTN4_1b1 in buffer, CTN4_1b2 in buffer, CTN4_1b1 in DBS extract, CTN4_1b2 in DBS extract.

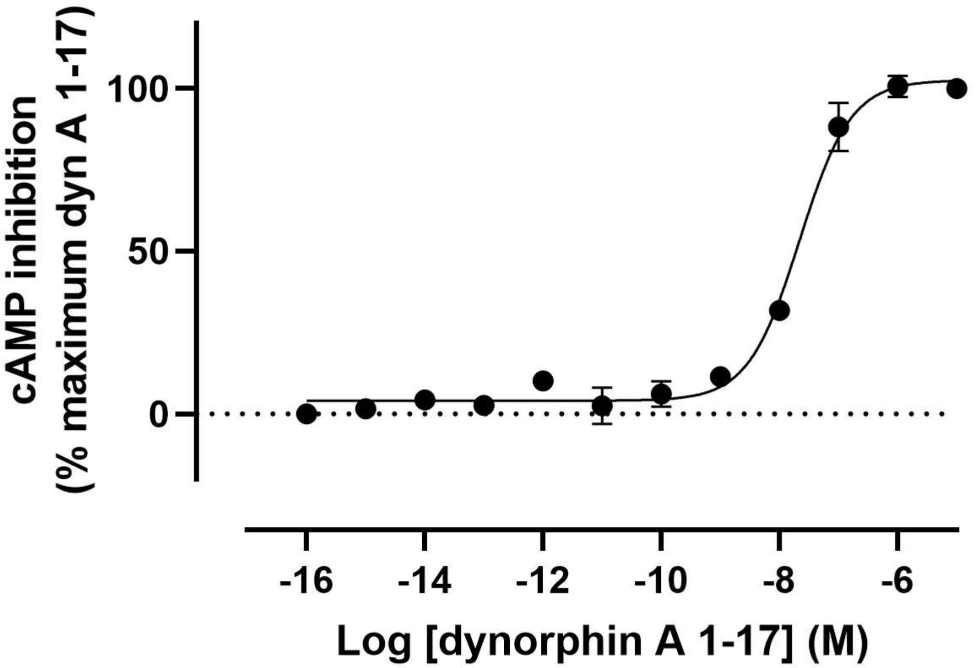

Fig. S19: cAMP assay of dynorphin A (1-17). Gi-protein mediated cAMP inhibition was measured following KOR activation by dynorphin A (1-17). Dynorphin A (1-17) activates KOR in the cAMP assay with an EC_{50} of 23.1 ±1.04 nM (mean ±SEM, n=2).

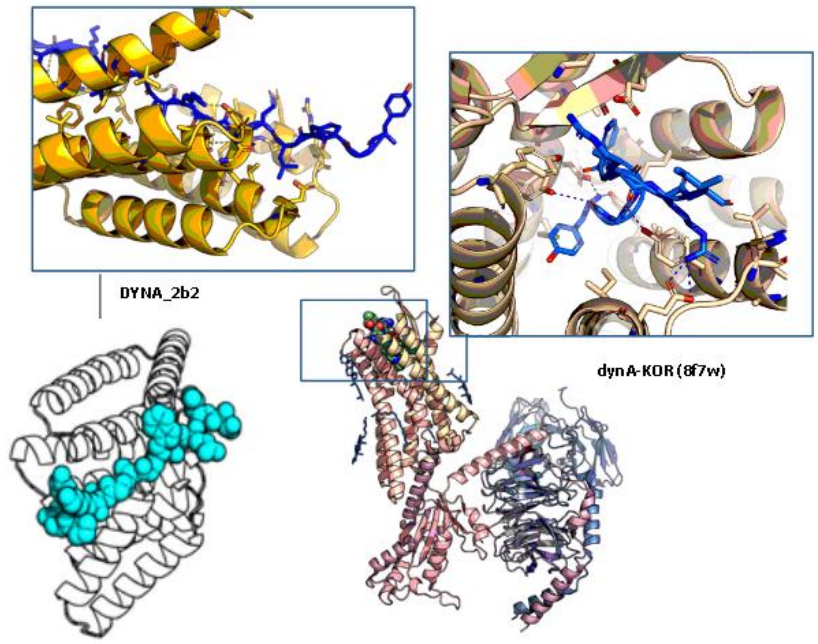

Fig. S20: Comparison of the binding modes between designed binder DYNA_2b2-DYNA and native receptor KOR-DYNA. By structurally comparison, **antagonism of** dynorphin A-stimulated KOR signaling by DYNA_2b2 binder happens through designed binder (left) binding and occupying almost full length of dynorphin A (shown in cyan spheres), out-competing with the native KOR-DYNA interaction (right) when KOR deeply associated with DYNA through the N-terminal YGGF- motif (left out in design for homologous specificity concerns).

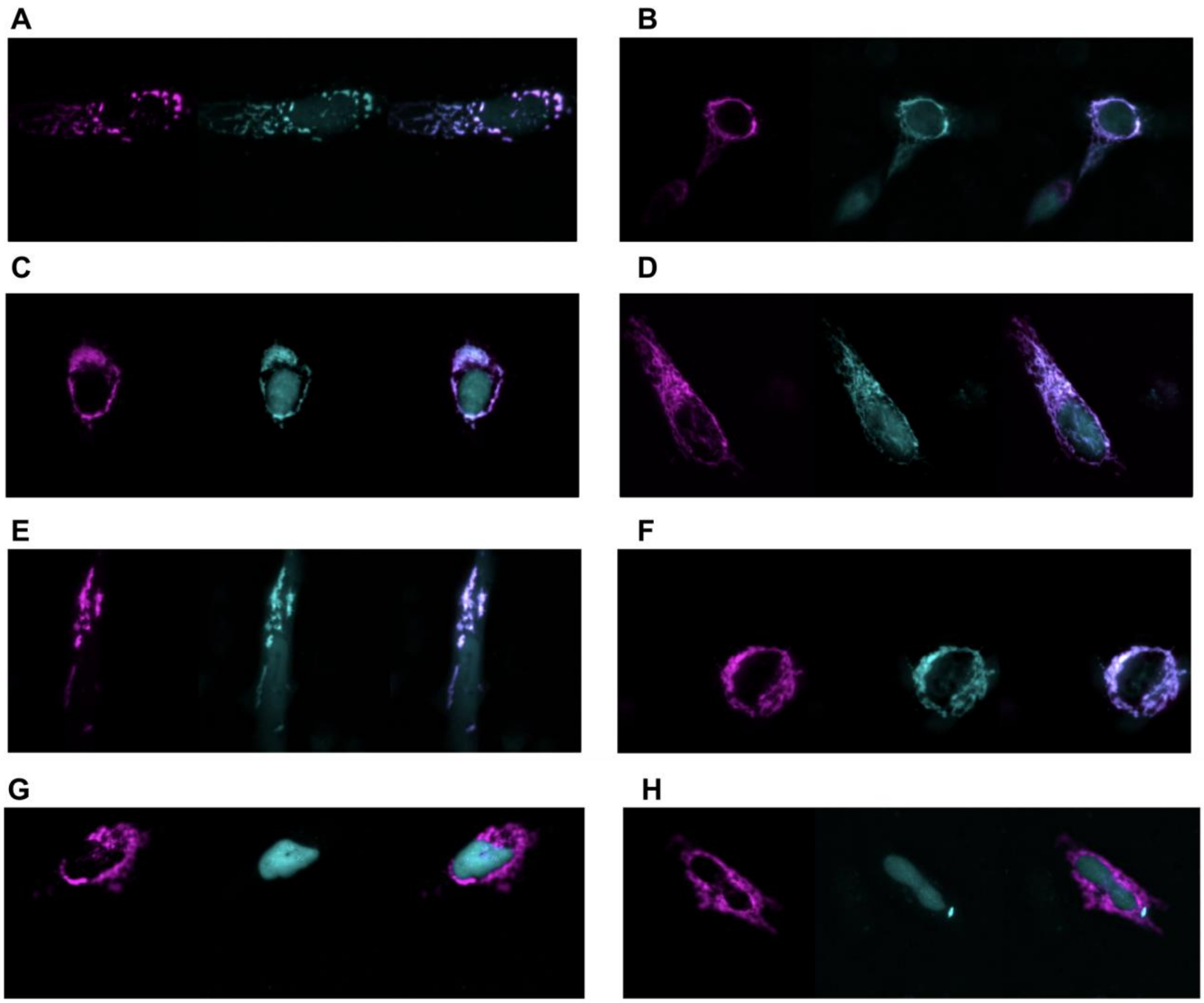

Fig. S21: mitochondria colocalization for six designed pairs of the native IDR binders in HeLa cells. 1:1 mix of plasmid encoding targeting disordered region-mCherry and a mitochondrial outer membrane targeting sequence (left image in each panel, red) and plasmid encoding designed binder-GFP and an N-terminal EF1a promoter (middle, green) were co-transfected and shown as colocalized to mitochondria under the merged channel (right, purple) within the corresponding intended design complexes as A-F, which are, **A),** dynB, **B)** dynA, **C)** SP, **D)** MSLN, **E),** AT1; **F)** C peptide. By contrast, design to dynA co-transfected with point mutations of off-target in **G)** and **H)** showed no colocalization when merge. Panels B, G, H are those shown in Fig 5E.

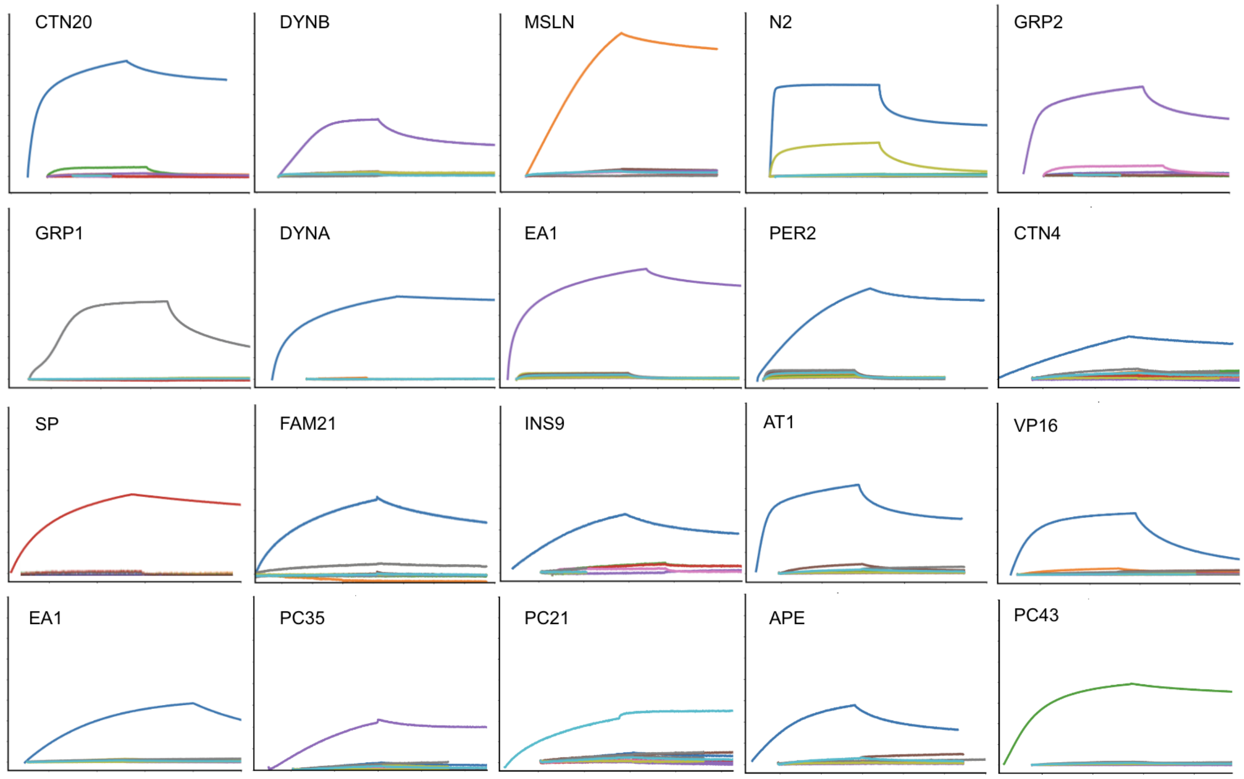

Fig. S22: BLI specificity test of 20 binder-target pairs with Kd under 100 nM. In each panel, one biotinylated disordered target (as labeled in upper left corner) was loaded onto the streptavidin (SA) biosensors, and incubated with its designed cognate binder and other 19 non-cognate binders all at 1000 nM in solution to measure association and dissociation. Traces were pulled together from multiple individual runs, which have varied association and dissociation times on x-axis. All binding signals were plotted using the same y-axis range (0.0, 2.0 ABU). Among all 400 individual runs, only the cognate pairs were showing obvious binding signals; with the exception of target N2 also binds to designed binder CTN4b1 to some degree (light green curve), though the non-cognate binding signal showed much smaller with a faster dissociation.

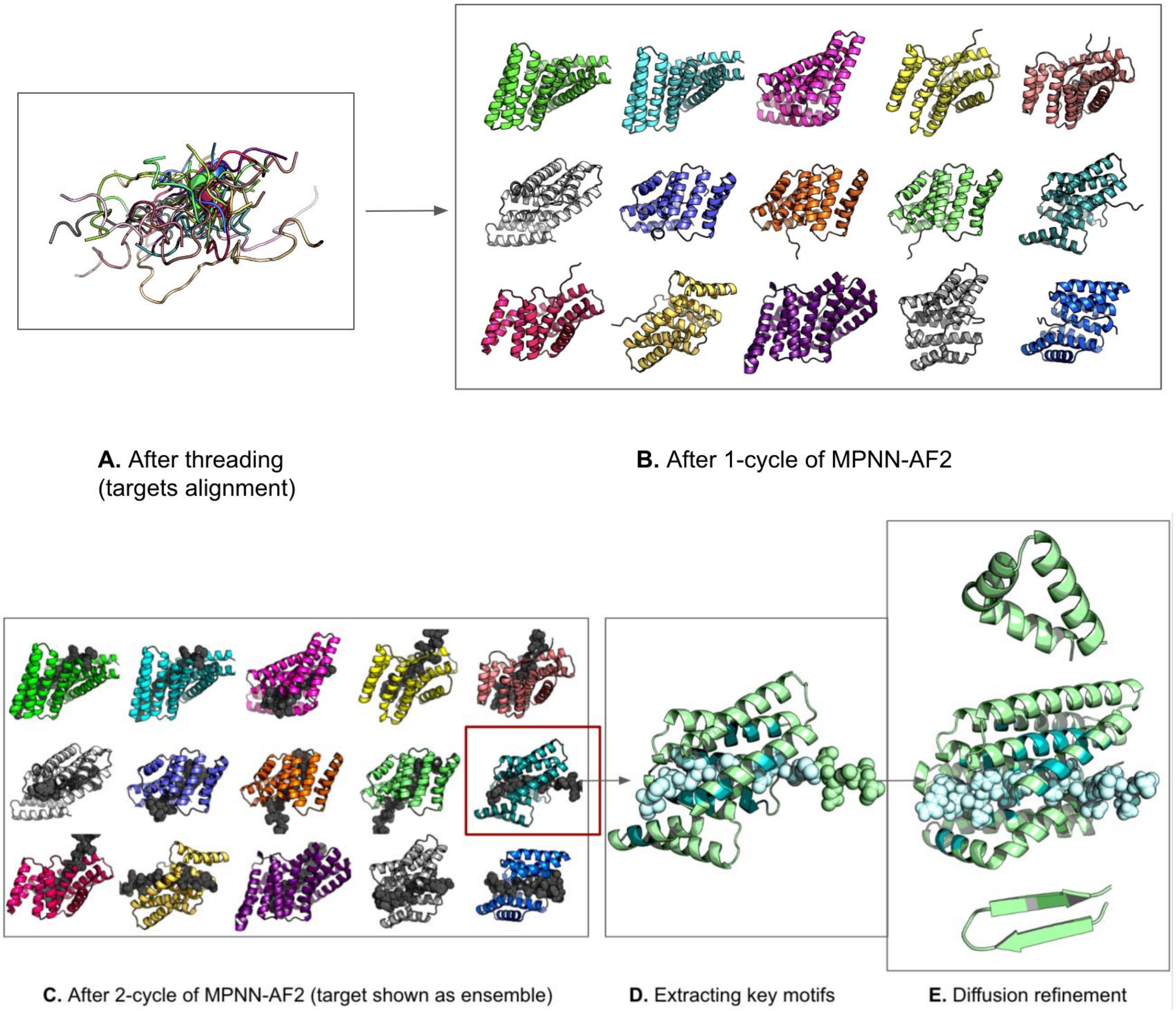

Fig. S23: An illustration of how design structural components could evolve during a complete design campaign. Here, we highlighted the steps structural evolving might be seen clearly either on the target or the binder side. **(A)** after threading a window of the target into the logos template library, a diverse ensemble of the target conformations was shown when aligned. Each color represents a different design; binders were omitted for simplicity. **(B)** after one design cycle of MPNN-AF2, a random collection of passing designs. Each color represents a different design; the target in each design adopted a fixed conformation (shown in cartoon) close to the target template from the library. **(C)** after two design cycles of MPNN-AF2, a collection of the same passing designs as in B. Each color represents a different design; the target in each design adopted a small ensemble of conformations (shown in spheres) close to, but differ from the target template from the library due to local resampling. **D-E,** major changes of structural elements normally happened during “diffusion refinement”. **(D)** from a finished passing design, key interacting motifs (cyan) were selected and kept on

both chains; all the rest of the binder (green) would be reconnected through RFdiffusion. **(E)** an example of refined design (middle) with a significant percentage of helical bundles rearranged. In cases, more dramatic changes on the binder side like connecting small, scattered motifs through overall repacked helical geometry (top) or strand pairing (bottom) could happen. Target conformational changes were often local; if “two-sided partial diffusion” was applied, dramatic structural changes on the target side could be observed.

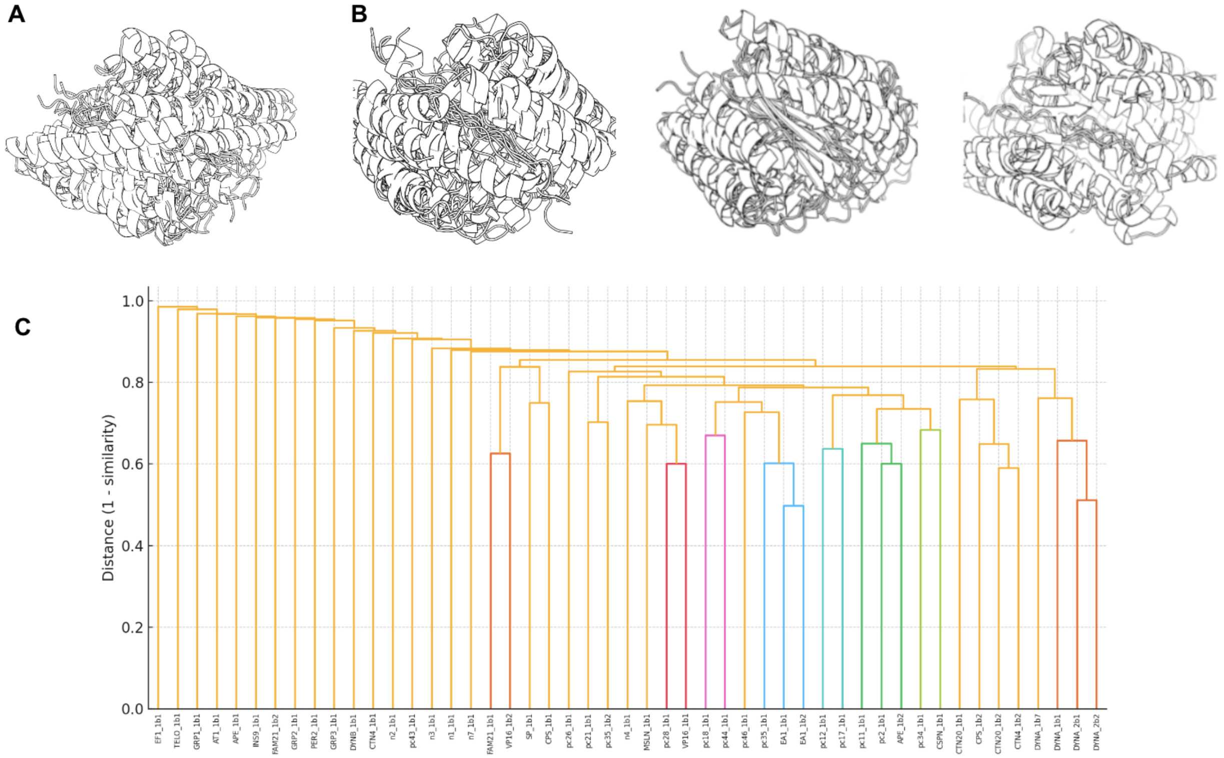

Fig. S24: Structural and sequence alignment of the final designed models in this work. A) CA alignment of all the 18 complexes in **Fig. 2B**; **B)** CA alignment of all the 23 complexes in **Fig. 3A,** grouped into targets bound in random coil conformations (left), partial strand conformations (middle), partial helical conformations (right). General structural commonalities are shown inside each group however with sufficient local diversity. In **C)**, Sequence Distance Dendrogram. The dendrogram shows the hierarchical clustering of all protein sequences based on pairwise sequence similarity. Sequences are grouped by similarity using average linkage clustering and a distance metric defined as (1 − sequence similarity), where similarity is calculated via global alignment. Shorter branch lengths indicate higher sequence similarity. Clusters may reflect shared structural motifs or functional domains among the sequences.

Supplemental Tables S1-S5

	n1	n2	n3	n4	n7	pc2	pc11	pc12	pc18	pc17	pc21	pc26	pc28	pc34	pc35	pc43	pc44	pc46
**n1**	2.2E-09	1.02E-06	NA	NA	NA	1.5E-07	NA	NA	NA	NA	NA	NA	NA	NA	NA	NA	NA	NA
**n2**	NA	4.64E-09	NA	NA	NA	NA	NA	NA	2.6E-07	2.5E-07	NA	NA	NA	NA	NA	NA	NA	NA
**n3**	NA	NA	9.42E-08	NA	NA	NA	NA	NA	NA	NA	NA	NA	3.84E-05	NA	NA	NA	NA	NA
**n4**	NA	NA	NA	1.9E-07	NA	NA	NA	NA	NA	NA	NA	NA	NA	NA	NA	NA	NA	NA
**n7**	NA	NA	NA	NA	2.6E-08	NA	NA	NA	NA	NA	NA	NA	NA	NA	1.5E-06	NA	NA	NA
**pc2**	NA	NA	NA	NA	NA	1.2E-07	NA	NA	NA	NA	NA	NA	NA	NA	NA	NA	NA	NA
**pc11**	NA	NA	NA	NA	2.7E-07	NA	5.6E-08	NA	NA	NA	NA	NA	NA	NA	NA	NA	NA	NA
**pc12**	NA	NA	NA	NA	NA	NA	2.1E-07	5E-08	NA	NA	NA	NA	NA	NA	NA	NA	NA	NA
**pc18**	NA	NA	NA	NA	NA	NA	NA	NA	2.2E-08	NA	NA	NA	7.6E-07	NA	NA	NA	NA	NA
**pc17**	NA	NA	NA	NA	NA	NA	NA	NA	NA	5E-09	NA	NA	NA	NA	NA	NA	NA	NA
**pc21**	NA	NA	NA	NA	NA	NA	NA	NA	NA	NA	6.3E-08	NA	NA	NA	NA	NA	NA	NA
**pc26**	NA	NA	NA	NA	NA	NA	NA	NA	NA	NA	NA	2.1E-07	NA	NA	NA	NA	NA	NA
**pc28**	NA	NA	NA	NA	NA	NA	NA	NA	7.8E-06	NA	NA	NA	2.7E-08	NA	NA	NA	NA	NA
**pc34**	NA	NA	NA	NA	NA	NA	NA	NA	NA	NA	NA	NA	NA	2.3E-07	NA	NA	NA	NA
**pc35**	NA	NA	NA	NA	NA	NA	NA	NA	NA	NA	NA	NA	NA	NA	1E-07	NA	NA	NA
**pc43**	NA	NA	NA	NA	NA	NA	NA	NA	NA	NA	NA	NA	NA	NA	NA	6.2E-08	NA	NA
**pc44**	7E-06	7.5E-06	NA	NA	NA	NA	NA	NA	NA	NA	NA	NA	NA	NA	NA	NA	2.7E-08	NA
**pc46**	NA	NA	NA	NA	NA	NA	NA	NA	NA	NA	NA	NA	NA	NA	NA	NA	NA	3.1E-08

Table. S1: Kd values of nanoBiT assay from Fig. 2B. During the all-by-all (18x18) orthogonality titrations, all the target peptides were held constant at [lgBiT]=1nM, while the designed binders were titrated at threefold dilutions of [smBiT]=333nM, 111nM, 33.3nM, 11.1nM. The plates were incubated overnight at room temperature before adding the substrate and immediately measuring luminescence. Each experiment was repeated twice, the averaged luminescence signals were calculated for plotting.

Number of Targets	Optimization	Initial computation		Computation-experiment cycle	
		Base	Diffusion refinement	one-time	two-time
25	×	✓		✓	
2	×	✓			✓
11	×		✓	✓	
1	✓	✓	✓		✓

Table. S2: Summary of computational and experimental protocols. Binders to 25 out of 39 targets have not been experimentally or computationally optimized, and produced straight out of the computer through the base design pipeline. Binders to 2 out of 39 targets have not been experimentally or computationally optimized, and produced straight out of the computer through the base design pipeline, but were experimentally tested in two batches to slightly increase the tested number. Binders to 11 out of 39 targets have not been experimentally or computationally optimized, and produced straight out of the computer through the base design pipeline followed by the diffusion refinement. Binders to 1 out of 39 targets (DYNA) went through experiment-guided computationally optimized. In the first batch, they were produced straight out of the computer through either the base design pipeline or diffusion-refined pipeline; in the second batch, optimized designs were produced by diffusion refinement on the best experimental hits from batch one.

Table S3A

Target name	Target sequence	Binder name	Binder sequence	pI_ Binder	pI_ Target	Target PD[illegible] available
n1	LKLKLKLKLKLKLK	n1_1b1	MSGDKKIEEAKETAEIAKENAEKAKKEGNEKAVKYAEEGAEEAKKAIAAYKKGDQKLAEAYETLAIAKTNAAAAYIEGNEKAVKYAEEGAKTAEKAIAAYKKGDQKLAEAYETLAIAKTNAAAAYIEGNEKAVKYAEEGAKTAEKAIAAYKKGDQKLAEAYETLAIAKTNAAAAYIEGNEKAVKYAEEGAKTAEKAIAAYKKGDQELGEAYERLAEALTNAAVAYIEGNEEKVEKYEKEAEEEKERIKEIEKKGSHHHHHH	5.18	10.78	NA
n2	PVPVPVPVPVPVPV	n2_1b1	MSGDEAEEAKKKAEEARKKAEELREKGDKELAALLEGLAASYENLAAGLEAGNEEAVKAAKKAAELFEEALKAYEAGDKELARLKYGLGASYNNLAAGKVAGNEEAVKAAKKAAELFEEALKAYEAGDKELARLKYGLGASYNNLAAGKVAGNEEAVKAAKKAAELFEEALKAYEAGDKELARLKYGLGASYNNLAAGKVAGNEEAVKAAEEGKELFEKALKAYEAGDKEEAEKLYEEGKEYNNKAAKAVLGLGSHHHHHH	5.04	5.95	

n3	YDYDYDYD YDYDYD	n3_1b1	MSGNEEAKKLKEKGEEL YKKAKEEGNDEVLLRIG GAYNNLGEAIEKGDKEA AKYAKEGLELYEKAKKE GGDRVLLRIGGAKNNLG VAILKGDKEAAKYAKEG LELYEKAKKEGGDRVLL RIGGAKNNLGVAILKGD KEAAKYAKEGLELYEKA KKEGGDRVLLRIGGAKN NLGVAILKGDKEAAEKF KKGLELYEKAKKEGGDE ELLKEGAKLNNEGIELLK GGSHHHHHH	8.93	4.05	
n4	GAGAGAGA GAGAGA	n4_1b1	MSGHEKEEKEAKKVLEA AKKNKEEAKKKGKKEAK KAAEEAEKLAKKVIEELP HVKTKEEKEKLELDLLIA KALVNYANAIIKGKKEAA EAAKKAIELLEEAKKLVG SKDPKKQEEREDLIQKA KAYINYANAIIKGTEAAE VAKEAIKYTERHKKEKD PKRHELYDQLAKAYINK ARAIIKGNKEAVEKLEKA IKILEELIKNPDHPDYEKL LKEAKELHEEALKLIEGS HHHHHH	9.12	5.53	
n7	RTRTRTRT RTRTRT	n7_1b1	MSGEYEEQLKQNEENY EIQKEEGSEGYEDAEKG VEYVKLAIKTGSEYYLQL GQNLQNLGIQKEEGSE GYEDAEEGVKYVELAIK TGSEYYLQLGQNLNNLG IQKEEGSEGYEDAEEGV EYVELAIKTGSEYYLQLG QNLNNLGIQKEEGSEGY KDAEEGVEYVELAIKTG SEYYLELGQALNNLGIY KEEGDEEYKEYEEQVE EIEEKIKEGSHHHHHH	4.19	12.0	

pc2	LKLKLKLKYDYDYDYD	pc2_1b1	MSGDLEDAEENLEILKEEGAPEEAIKAAEESVKILKKKERTEEEDIKARALNNRAIAIAKGSEAAEIAEEALKVAKTGDLTKARAYVNLAAAKLKGVEEAIEYAEKVINEDLPETLKRAYVNAAAALLYKSEGYEEAEKALEEAEKAIELIKEGDPEYKKHINEAAELLDKIEGSHHHHHH	4.66	6.2	
pc11	LKLKLKLKPVPVPVPV	pc11_1b1	MSGNEEEREELREKVRENLELMEEEGSDNEEAREAYERALELLERGELTRARALHNLGVALDEGNEEAIEYARKATEEEDELEATALINIAVALIEKERGYDVEEAIEYAEKALELAREGKELEAAAYINLAVAKIEYETEGNEEALKAAEEALELIEKGEPEEEVKKAINEAVKLLGSHHHHHH	4.56	10.48	
pc12	LKLKLKLKVPVPVPVP	pc12_1b1	MSGEELEKEIEKEAEETEEAIKTAVEKGKISEEEAEKIKKELKELKEKKSELAVNLISTKYNYIVFQLQGNEEEAEELKKAIEEAKKLAEEGKTDEANLLLTLQNNVKIAKHLGLDEEEVKEIEKKFKKALEEGKYLKATLLIYELVLKILEKAGNKENLEEAKKILEEAKKNYKETGEVDYELLVKLHEVLKEAVEKFEGSHHHHHH	4.93	10.48	

pc17	DYDYDYDYVPVPVPVP	pc18_1b1	MSGDEELRKKLLEKAKEYLEEAKKKGNEEAVKKLEEAIKILESGGNLELAVALYNQALAIMEGTSTEYFEKAIEAAKEGNEYAAAAWINAGIAEAREELGEEVNPKAKEYAEEAAKLAEEGGDPIRVAALINASVGLLLGVPEEEVERVLEEIERVSEEEAREIVNRYYERLKKKLLERLGSHHHHHH	4.66	4.05	
pc18	DYDYDYDYPVPVPVPV	pc17_1b1	MSGRIENADDPEEVIERMRREAERVDPELAERLRELAEELERDLREGRLDDSKLLRYIARRNEILAEPLRETDPEAYEEIREDLERAKEEAENLENDTDLLIAVAYINAAAALATRAQGSPLPAEVAERLRELAERLEEEEPSEEEKLEAVAEIAAAAALGLAYQRDLPEAKELAERLREAAERLRRGEISREEAVREIREVVEELKKLGSHHHHHH	4.52	4.05	
pc21	LKLKLKPVPVPVDYDYDY	pc21_1b1	MSGEEELEKELETLRKAVEEKKKKDPENKEKYDEILELLDEAKKSDNRVLKELTLNYAIALFNEEEEEVKELLKEAVESIKKNPNKKSKFLAKLLNNLAVTRVEAKEDPSYKPLLELLEEALELAKNIKTKEEYLEVLLKNLLAIAKILGLPEEELEEIEELIKKIKELPIEEVEEKIKKFYKLIKEKQGSHHHHHH	4.96	6.04	

pc26	LKLKGAPVTQYDYDRTRT	pc26_1b1	MSGLKKERERLIKILKENKEILEEEGNDIPELDEIIEKLEEANPETLPLLLTRAALLNNLAILKQEGGDEEDIEEAEELLEKLEEAIEKKKEGTISEEEVEKLLLEASFFNNLLLLKSAGNEEEAEELKEKFEEALETNEKHAKAINAILLLEYLKHEVENSNNISEEEKEEYLEKLEEAIEKLEEALEEGEDPTPYIKEAQKVISEILEKLEGSHHHHHH	4.33	9.52	
pc28	DANIELSILVA	pc28_1b1	MSGFEEKVKKNLEELKKSGDEEIKKLVKEAEKEIKEGGSKLLVSLLINRALALKDGDEELAEELEEQIELIKKKKKLTQKEKLLIVLKNNRAAAVIEGDEETAKELEELIKVVEEEELTDRDIVLLTLKNNALIAKMNGLEIEKEAEEIAEKLESGEISLEEAKEKAEEIVEKIDEYGSHHHHHH	4.65	4.05	
pc34	HIALIENFRIEND	pc34_1b1	MSGREERRKEEIEEMEKVLKQLKEEGKEEEAKELEEGIELYKTGEEDDRALGRIIYNRAVAILEGKEEAVEILKEAIELYKEGLEKGDRRKRIRALILANRAVAVIEGKEEAVEEAERALEIFETENPSFLDQLLLLNRVAAVIKGDEETAERLRKAYEDPSTTEEEKREVIEEAYERLSGSHHHHHH	4.65	4.83	

pc35	DERRICKHICKS	pc35_1b1	MSGSSEEEDREYIEEREARNPNPEARRYLEELRELTERGPRTKREHLRAALLTARAEDAERGGENRPILDEAEEALEEGDLERARLLLAIVLARRLGFEELAERLRRLLEEDAPERRRRIELARLLALFLEKEGHPRARELLERVEEAERLYEEGDPRAEPLLEETEREIRELILGSHHHHHH	4.35	8.9	
		pc35_1b2	MSGENIEIVNEVLEELSEILNKELGLPKLPKIEKDNEESWKKALELLEEYVEKVKGHPIEPYVKNAYLSVAALYAEKIEKDNVKIFEYELESLVVLAEILGYPEEKIKELKEIAEKTKKLVEPLVKKQKLKLEFELIDLSNPEEVEKLKEFLEKRFELLKKLVNGEITPEEFIEKLNELAEEGSHHHHHH	4.75	8.9	
pc43	DAVIDLIKESPEPTIDE	pc43_1b1	MSGDKEEELKKLKERAKKVIEKLKKKLEEEKDPERKERLKKIIERIEKALKEAEDGSEENLIRLYIEIAIAVLYVEGKEEEAEELEEILEKFEKEYEKLKKEGKPKEELEYLAYKALAEALIILAKAAGFEELAEEAEERLKKAEKLAEKGLIEAALESTKAIIVILKTRVLLEKGLVPEERKPELKEKIEKVEKLLEELYKAVEEGDKEKIEKLIKELKEAIKELIKLRKEALKEKEEKGSHHHHHH	5.25	4.05	

pc44	DAVIDITISTIME	pc44_1b1	MSGVKLRLRADEELVKKTEKSLPEDDPLKKKYEEAQKKGNKNLALAYLLKYALRKKTESAPEELRKRLEELLDKFIEAIEKGDVAEAVAYLLFVYAEELKQQGEDEELIEDLEYIAEKVQKRGSELDIRLAALAIEVVEKYLSNREEFDEEEAKELLRLLREALDKPDEEKEKVIKEVEEKLKKGSHHHHHH	4.92	4.05	
pc46	WILLCHEN	pc46_1b1	MSGVKTTRLPGVVIIEAETKEEAREEAKRQGLEVVYEMSFSREEIEELLKEFRKDPRTRPVADRVEKLEKEIETADLETAVKLFREIDRLLEEAKRGSHHHHHH	5.01	5.24	

Table S3B

Target name	Target sequence	Binder name	Binder sequence	pI_ Binder	pI_ Target	
DYNA	YGGFLRRIRPKLKWDNQ	DYNA_1b1	MSGEEEKEEKYLKEVIETIEENLKEAKEEGDEESAELLEEALKIAKEAKETKDREKSKMLLVIAILLANLAVAIHEGKREFEEDLLKYAEELKKEVEEGKFNEKLLIESTALNNLVVRALEFNLPYEEVEKELEKVKELLEENPLLAQAYNVIIELEYVIESGEANEEEKKELEKYIKKLEELIEKYKKGEVSEEELKKAINEAYEKIEEIEEKSGSHHHHHH	4.53	11.0	

		DYNA_1 b7	MSGKEEEIEKEFEEKKKIIEENLKE AEEEGEEEAAEKLKEALKKLEEAIK LHREGANPVEVELEEVTAIILNNLA VLLREGEEELAKELEKAIKLLEEKK DAPEEERLKAIAIAIIRSVLVLIKWE GGKDEETIEEIEEILENRENLSLEEL REAYVRAEIAYLIESGIDPEAAKKV REKYERGAPLEELLKDIEKIEKEGS HHHHHH	4.56	11.0
		DYNA_2 b1	MSGEREKLIEIIEENLKIIGEDNEKL KKALEIFKSGANKVEQLKAIALNNL AIAEHEGSEAVELYKKAIELLEKAE KTGDKNYQLAAVAYINAANAIEEG LPEEDVETLKKAAELAQKNPNERE KLQIIALINYVVAKIEAKEGKKTEKY VPLLEKTAKDLKEGKISIEEAKKIIN EAYEEGSHHHHHH	5.08	11.0
		DYNA_2 b2	MSGKVVLAVDPEDVIEIIEENREKI PKYEEELDELIEEAEELIESGTSTD EKLALIIAKYNLLVTEAELEKDPENE LLKEAAELLKKAIEILEEGGDYLAA VAYINAANVLVEIARGGPLTEEDVE ELEEIAEKIEEGDLDEESEREAVKK INRVVEKIEHGSHHHHHH	4.13	11.0
DYNB	YGGFLRRQ FKVVT	DYNB_1 b1	MSGKERVVDVVAGNLVLEIDREEL REKIEENSKKDPRFKKILEEFEKNE EDPIKLLLAEAEYNLVIAEELNSEA VPYFEEAVRILRKAIEEGKKSLSEE ELLRINALMTAGIAEEEGLREDAEE LARLARDIEENRDSLSEEELLRYVI KINLTQARLQALEGRAKEERERLE KALEDESIPVEEKKKIINEAAEKLG SHHHHHH	4.63	11.0
MSLN	NGYLVLDL SMQEALS	MSLN_1 b1	MSGKEELKKRLEELKKQGPSPEA KKYLEEAEKILEKENLTTKEKLLAA LYINLALALIKGDPVTPLLKRGIEAA ENGDLATAAALNNIAILHVNGHPEA PELEKRLEEVKDNPEAVLLLNYEMI AKIYKQQGEKEAEEILKLVEKAKEA RERGSPEFEKYMEEAEKLIKKIKN GSHHHHHH	5.53	4.05

SP	RPKPQQFFGLM	SP_1b1	MSGIRIDEELLHKGLKEAVEYDPELFQPILDRFEELKKSGKLDDKSKVIILIEYNRRVALVDGDEETAKRFDELIKRAEKAKTREELVEVLVENNLVIALSEGIITEEEAEEIKELLEKALKTGDRRYKLKLRATLYSKVIIAYELKRAKEEGDEETAKKLEELRKKAEEILEKLEKGADLEELEKELEKIEKEYLELQKEKGSHHHHHH	4.94	11.0
CTN20	ESSVSLTVPPVVK	CTN20_1b1	MSGSEEIEQLLKDQEEYLKSQDKEYLKKVAERNLKVAEEKGVEEAIKAAKEAIELLNKENLTAYDVLKAIALINKAVAKIKGVEEAAKYAEEAVKVLEEAEKEGSLELLLKAIALINAAVAKIKGVEEAKEYAEEAIELLDEVEEEDLSEEERILKILLAIALILAAVAKILGVEEAKEYAEKAIEILKKMIKEGTTEKREEAIELIRKAQELVEKKIKEKRRGSHHHHHH	4.88	6.1
		CTN20_1b2	MSGEEELKRLLEEQKEALKNQDREYLKKVAERNLEVAKRRGNEEAQKYAQKAIELLEKEDLTAYDVLKAIAYINAAVAKINGNEEAEKYAEEAVKVAEEAEKEGSLVKLLKAIALINAAVAKINGNEEAAKAAEEAIKILEEVEKEKLSPEEKLLKILLAIAYILAAVAKILGNEEAAKAAEEAIKILEEMEKEGTTEREEEARKKLKEAYKLIEEKEKKEKEGSHHHHHH	4.99	6.1
GRP3	VLWNSRWPTLQAWGAGLKPGY	GRP3_1b1	MSGEEEEEEAIEEVKEELKKTGTEEAKKAIEYYEKVEKELEKLSPEAQRLARIALHNYLIAVLQGNEEAKKLLTEVIETLLKNPNPSHEELLLLAAKLNAAAARLNGNEEAAKLAKELVEEIEKALKRGDTSPLLRLLAKNNLVAAIMEGNEETAERLRRAIESGDPEELRRVTEEAVEEIEGSHHHHHH	4.74	9.99

CTN4	NWLTIFILF PLK	CTN4_1b 1	MSGLDSWAESLTERVDELIEAITENLEKLKESTSPNAPEAVELAEEALELLQEVKTDDTLSPKDKALKILRAVALNNLAVAVNLGDKPEAVETAKKALESLKEAEEVDDTDPELAELLVLRAIAYNNRAVAILDGDEEAAEYAEEAAEKLEEAIELHEESKTSDDKEKLELLHELYTLRAIALIHKAQAILDGDEEKAEKAEEAIELAEEAIEEIKENPSEENLQKAQESQPEIQELINESVEGSHHHHHH	4.2	8.75	
		CTN4_1b 2	MSGDKLREKIEKNLEIAKEKGNEKAAELLEKAREALDKNRDLAVAYNNAAVAILLEEPEEVVEIAEKAIELLEKAKTEEEKLIAVALINLAVARIKARKEKRPLAEEAAKLAEEAIELAKKGKILEAHALILAAVALLLGEEELAKELIEIAEKLKKGTISEEEAKKIINEARERLEKKVGSHHHHHH	5.27	8.75	
CPS	GSSLQPLA LEGSLQ	CPS_1b1	MSGGTPQTLTGEVVIITEGKTTRVERQPATPVKVEPDDTPEEVAEKAREVLDELLQLAEERLLELVRENGGKEPVTYRVILLFDGPDDPEARRKLLEHGRRGLQEALEKAAKVRDELIGKPGSAKVTPPREVDPEEARKRIKEAGSHHHHHH	5.08	4.05	
		CPS_1b2	MSGEKLDERIRRLLEVAKEKGKEEAAEALEEALKPGVSKLKKQALYNYALAKLKGKEEAAEYAKKAIEILESGGNKAKAIALINAATAKLSGKEELAKELEELSENVDKKSPLEVALIINKAVATMLGLDTSKLDKAEELIEEGKEEEARELILEAYEELKKAVEERGSHHHHHH	5.07	4.05	

FAM21	SSDDDLFQSAKPKPAKKTNPFPLLEDE	FAM21_1b1	MSGPMDEAKKLKEKAKEYREKAEEYRKKAKELKEKGNKEEAQYYEDIADAYDLAALAAEQLARAIEKKDKEAAHLYREALEFAEKAVKLFEEAKKLYEEGNKEEAQYVDTIATALYNLAAALLFIPRAEKKKDKTSEELYSSAAHSAHEAIELAEKAYRLYKEGKKEEAQYFDTEAQALINEATALLFLARARKNKDEETEKKATEALELAKEAIKLVKEAHKLYREGKKKEAKKLLKEALEKINKATDILEEINKELDEKRRAGSHHHHHH	6.08	4.69
		FAM21_1b2	MSGDEAKKLKEEAEEALKKAEEYREKAKKLKEEGKKEEAEFYEDIADALELYAIALYELAKALEKGNKEAAKLYREAAESAKKAAELYEKAYELYKKGKKEEAEYLHDIATALHNLSVALLFIPRAREKNDPKQLELYERAAESAEEAIRLNEEAYELYKKGKKEEAEYLHTLAQAFINLSTALLYRARALKKNDEELIKKSEEAIELAKKAIELVKEAYKLYKEGKKKEANKLLKKALELNEKASEILEKLIEEEEKKGSHHHHHH	5.59	4.69
GRP2	HENGPWPGPCNARVAPMLLPRLPTPGVPSD	GRP2_1b1	MSGDEEERERVRKFVEEIKENLKPKIERSPDEERKKEIEELLKKAEEAAKKLESGDPYYVVAAYGYAAIAALLAEGLEEEAERLRKVLEELEKEVKKLKKEGAPQVKIDYAIENALLRMFIVLLKAEGFTELAEEAEKDLEKIEKVAKKGRYEEALAATRLLYYHYRLRYLVESGIATEEEKPELERLSEELEKLLEKLIKAAESGKEEEVKKLREEAKKLAKRARELIRRVLERHERRGSHHHHHH	5.52	6.75
VP16	DALDDFDLDMLPADALDDFDLDMLPA	VP16_1b1	MSGEVSEREVVTSAIKKAAEKTNEPEVKEIAEKVNDPSLSDIEKLELAVELFKYLAEKTGDEEYKLLSEFFEYLKRYKETGDEEYAFKAAEVYAEIKKRTIPEAAELLKAKLKVLKALKEKDPSVKFRLVFRPVNLRELAKDPEARRTREELTKEAEEAYEKLESGEISVEEAKKLIEELNKRGSHHHHHH	5.13	4.05

		VP16_1b 2	MSGSKEYFRKASERIKKEGETVEAYAKAVLDAAEKLLPEAEKNNDKEAAEYFKKAAEEAKEAVKLAKEGKEEEAKAKVVAAIANLLIPHARKNNDPELAKLFEEAAEKAKKGEVLEATALIVIAIAKHRNDEELLEKAKKALELAKEGKEEEARKLLEEAKKEQRGSHHHHHH	5.96	4.05	
INS9	SHLVEALYLVCGERG	INS9_1b 1	MSGKIYLRNDPEKVIEKMREQSEKVDEETSKELKELADELEELKKNGELDDSLLLQYIAKYNRLVSKPLKETDPEAYEQIQKDLEKAEEKAKNLKDDTDLLIAVAYINAAAARHTRAQGSDLPESVAEELIKLAEELEKNEPTEEEKLEAVAKINAAAALIIAHQKNKPEARELAEELEEAIEKLRRGEITLEEAKERVNEVAERLTELGSHHHHHH	4.48	5.38	
PER2	AVPFPAPVPAAY	PER2_1b 1	MSGEPDPREKVRVRLPPRELLLRQIRHAAEATGDPRFEEIAKELERLEGDDERELLRLVARYNRVAAEVEGFPEAAELAERALEVLDKGGPLLAAAAYNNAAIALVYAQRGHTELQPYAERLLEYAERLQTEEVDEREVLLFVAENNYVVAQILAIELERSGQDPTPARELAERLKEAIKLLEEGGDLEEAKKIINESVEELLRLYETLLSRGSHHHHHH	4.68	5.57	
		PER2_1b 2	MSGDPEMEEAFEIVIEKVLEVLKDEEKKEEIKKLIEEGASKKEILEKAIEILEEEYKETKDKEYEMTLLLVKAFYYNEIEKNQEKALEYLEKYAELLEELDPVLARYLKAQVELSKELLEKDPSVKLRILPKRINLEEFKKDEELRKKLEEQTEKLEELLEKLKNGEITVEEALKKKKEILEEGSHHHHHH	4.94	5.57	

GRP1	RRPWVPHLLPFSSPGYLGVM	GRP1_1b1	MSGDPRVVELKSGYLVFSIDYDALLKALREAANHDPRYRPFATRLERVRDDPVKVFAVWIGYNAEAARQLGLPSAPLLEELLEEVEKRIKNGEKTLPESLLLKAALAIAIAEAERAGLEEEAERLRELREEIEENPESINKEKTVDIVLEITLAEAKVLAKEGRAPELAKKLEEAAKDKSLSLEEKKELVDEISKKLGSHHHHHH	4.96	10.84
AT1	DRVYIHPFHL	AT1_1b1	MSGHKEITVGRIVLHVPDVEKLREHLKKKMKEAGVPEEEIEEFEEEIKNASPEKQFLLFMKKLAELAKKEGADEEVTERLIELLDELIENEENVDPWDLLEALALFLAIQAQLEGFEELAKQARELAEEIEEARERGAPEEELLRLAARLAALQAQLGVDLGKLPPEAKELAEEILKLVDSGAPLEEILKANRRLHELVKKSRGSHHHHHH	4.72	6.92
TELO	SWAHPGRTRGPSDRGFCVVSPARPAEEATSLEGALSGT	TELO_1b1	MSGREELLEGARKLLEEARKDPSTPEEALEALERAIRILEEGGDVLEAAAYYNLALALKQGNEEAVRLLERAEELLREGSEKARLEAAALVNLAALLLNGNPRVPEARRLLQLLPSLTPLEARLVVNYLAALVKGNERVAERLEEVIRRIKNGEEVPREEIVRAVNEAVEELGSHHHHHH	4.38	6.49
APE	QRPRLSHKGPMPA	APE_1b1	MSGNPFREWLERNARREEETFEEEVEEIREEVEELLESENEEVREIAERIREVLEEAERAREEGDWVLAHLYLEKAKALLNLALALMEDAPERVIEVAEKAVEAYEKAIEAYREGDWERAAAYYVAGDAYNNATVALMRGREELAERLIEEGERLRKQAEEGKVDRRELERAIALNNAAVADILSEEYEERDPELAERFREIAERFRRAAELIDEGREEEARRLAEEAYRELEELLESLGSHHHHHH	4.45	12.0

		APE_1b2	MSGKIVLKNDPEEVIEKMRETAEKVDEELSERLRELAEELEELVERGELNESKLLLYIAEYNRIVSEPLKETDPEAYREIQEALDEAERHARNLRNDTDLLIAAAYINAAAALITRAQGSDLPEEVARRLRELARELEETEPSREELLEAVALLNAAAARILAAQENKPEALELAERLEEAVRELREGRISLEEYRERVNEATEELVKLGSHHHHHH	4.45	12.0
EA1	PTPGKGPKVYRRKHQE	EA1_1b1	MSGRLEIVIDEEELEKTIKENAKENPEVYQPILDEYEKLKKEGKITEKNLLLLLAKLNLKVAEEKGNKEAVELMKEAIELIKNAKSEYDLAEAALLINAALLLLEGREDRETFERLLKILKEAEETGDERLELLFAALYNAEFVAKPLAEEAKEKGKEELAKEFEKAAEKAKKAAEIVEKGGDYDEAQKLHDEALKLIEELEKKLGSHHHHHH	4.46	10.45
		EA1_1b2	MSGMLEVHVDEEELRKEIEENAKENPEQFKPILDELEELKKEGKISSKSLLLLSTKHNLRVAESKGNKEAVEALKKALELIENAKDSYELAEAAVLHNYAILLLKGKRDEETFEELQRLLREARETGDPRLELRFASLYNRHFVAEPLAEEAKKKGKEELAKKFEEAAEKFEEAARIVEEGGDVEEAEKLHEEGYELIEEVEKKLGSHHHHHH	4.29	10.45
CSPN	DNEKLRKPKHKKLKQ	CSPN_1b1	MSGDKEREELAEVIEENKKFLSEEDQKEAEKLIEKLKDPNTTEQEVLHIEAKSLLLIAKEEGNKEAKELSEELIKEIENGEKNEEKLIAKINAIVALLNGEKEYAEKFKKLSEEWDKAKTEKEKVLLIAKHNRLVYEIEAKTTGDEKYKEKAKKLEEIIEKLEKGEISIEEAKKEIEKLLEEGSHHHHHH	4.91	10.3

EF1	SQQSSSYGQQNPSYDSVRR	EF1_1b1	MSGREKKIKKAKEYLEEAKKEGNEEAVKYLEESIKILESGGNKVEAVALYNAGLAKMEGVSTENFEKAIEAAKKGNELAAAAWVNAGIAEARRELGEEVDPRAEEYAREAAELAEKGGDPIEVAALVNASAGLLLGVPRERVEAVLEEIKRVSDEEARRLVNEFYEELLQRLEERGSHHHHHH	4.54	8.31

Table S3C

Target name	Target sequence	Binder name	Binder sequence	pI_ binder	pI_ target
n6	RDRDRDRDRDRDRD	NA		NA	6.29
S15a	ELYTSTSPSSSSSSPLSSSSSSSSSSSS				4.05
GLN12	QQQQQQQQQQQQ				5.53
CD52	GQNDTSQTSSPS				4.05

Table S3. Summary of all target sequences, binder sequences, all target PDB available structures, and similarity of targeting conformation(s) and available conformation(s). (A) toward 18 synthetic peptide targets. **(B)** toward 21 native disordered targets of successful attempt in this work. **(C)** toward 4 disordered targets without successful attempt in this work. As for similarity score in the last column, 0: no backbone similarity for more than half of the target residues; 1: observed backbone similarity for more than half of the target residues. Calculation was carried based on torsion angles.

Target	Kd (nM)	Kon ($M^{-1}s^{-1}$)	Koff (s^{-1})
n1	<0.5	3.6e5	1.6e-4
n2	18	2.3e4	4.4e-4
n3	72	1.0e5	6.9e-3
n4	>350	2.2e6	7.6e-1
n7	5	2.2e4	9.7e-5
pc2	84	6.8e3	5.7e-4
pc11	50	1.2e5	6.1e-3
pc12	36	5.2e4	1.9e-3
pc18	10	8.5e3	8.9e-5
pc17	45	6.0e3	2.5e-4
pc21	27	3.4e3	7.7e-5
pc26	>200	3.1e6	6.1e-1
pc28	9	1.1e4	9.3e-5
pc34	180	6.1e4	9.8e-3
pc35	90	7.0e4	5.6e-3
pc43	9	6.9e4	5.6e-4
pc44	35	2.4e4	8.9e-4
pc46	37	1.4e4	5.8e-4

Target	Kd (nM)	Kon ($M^{-1}s^{-1}$)	Koff (s^{-1})
dynAv3	<0.06	1.4e6	8.3e-5
dynAv1	<1	2.7e4	2.1e-5
dynB	<1	4.3e4	2.9e-5
MSLN	<1	3.8e5	3.4e-4
SP	7	2.9e4	2.0e-4
CTN20	9	3.3e4	3.0e-4
GRP3	10	7.4e4	7.8e-4
CTN4	25	3.1e4	7.1e-4
CPS	33	6.0e3	2.0e-4
FAM21	35	5.1e3	1.8e-4
GRP2	42	2.1e3	8.9e-5
VP16v2	45	4.3e4	1.9e-3
VP16v1	19	2.2e4	4.3e-4
INS9	59	1.5e5	9.0e-3
PER2	67	5.6e3	3.8e-4
GRP1	72	1.2e4	8.7e-4
AT1	75	8.9e4	6.7e-3
TELO	91	6.9e4	6.4e-3
ape	92	3.5e3	3.2e-4
EA1	100	3.8e5	3.3e-3
CSPN	195	1.4e3	2.7e-4
EF1	>500	1.5e6	7.4e-1

Table. S4: Summary of Kon, Koff values of designed binders to all the 39 targets in this work. Octet, BLI measurements of twofold or threefold titrations were carried out as described above in Fig. S3, S7. Steady-state and global kinetic fits were carried out using the manufacturer's software, Data Analysis 9.1, based on the assumption of a 1:1 binding model.

Sample	Area	RT	Loss (%)
Peptide Standard (loss)	90889	3.12	
CTN4_1b1 Sup	338	3.07	0.4%
CTN4_1b2 Sup	122	3.09	0.1%
Non-Binder Sup	85493	3.13	94.1%
Beads only Sup	27697	3.14	30.5%
CTN4_1b1 Wash #1	5684	3.13	6.3%
CTN4_1b2 Wash #1	8280	3.13	9.1%
Non-Binder Wash #1	5733	3.13	6.3%
Beads only Wash #1	45717	3.13	50.3%
CTN4_1b1 Wash #2	86	3.13	0.1%
CTN4_1b2 Wash #2	32	3.08	0.0%
Non-Binder Wash #2	22	3.08	0.0%
Beads only Wash #2	2347	3.14	2.6%
CTN4_1b1 Wash #3	0		0.0%
CTN4_1b2 Wash #3	5871	3.13	6.5%
Non-Binder Wash #3	14	3.06	0.0%
Beads only Wash #3	527	3.13	0.6%

Table. S5: LC-MS chromatograms for CTN4 binders. Statistical analysis summarized in supplement to Fig. S15.

Materials and Methods

Outline

➢ **Computational Methods**

I. Scaffold generation
1) Base scaffold generation
2) Hashing provides initial high-quality complex docks for ProteinMPNN

II. Pocket specialization
1) Base binders for di-peptide repeat recognition
2) Backbone library through truncation and extension
3) Backbone library through parametric perturbation
4) Backbone library through motif diffusion

III. Pocket assembly
1) Backbone pockets connecting through RFdiffusion
2) Assemble to target arbitrary sequences beyond pockets
3) Two-sided interface design

IV. IDR threading
1) Threading
2) Cycle of sequence design
3) Predictions
4) Illustration of the customized packages (MPNN, AF2)
5) Instructions and step-by-step guide

V. Refinement - Scaffold-refined Diffusion
1) Partial diffusion (one-sided)
2) Partial diffusion (two-sided for library construction)
3) Motif diffusion
4) RFdiffusion

➢ **Experimental Methods**

1) Gene Construction of Designed Binders

2) Protein production and purification (small scale and medium scale)
3) BLI assays for initial screening and affinity measurement
4) BLI all by all orthogonality matrix
5) NanoBiT assays for initial binding screen, affinity measurement and all by all orthogonality matrix
6) Protein Purification for Crystallography
7) TAMRA Peptide Synthesis
8) Biotinylated Peptide Synthesis
9) Fluorescence Polarization
10) Cell colocalization
11) Cell surface staining

➢ **Individual Design Characterization**

1) Purification of KFAM binders
2) Immunoprecipitation with KFAM binders
3) Dynorphin A inhibition cAMP assay
4) Affinity enrichment of CTN4 analyzed by LC-MS
5) NMR sample preparation and data collection of dynorphin-binder complex data
6) X-ray crystallography

Computational Methods

Due to the flexible nature of disordered regions, we considered them as polymer chains of arbitrary combinations of amino acids. We broke the problem of binding to disordered regions into two main components: 1) providing binding pockets for all 20 amino acids (pocket specialization), and 2) assembling pockets flexibly to induce any unstructured backbones to fit (pocket assembly).

Here we first describe the pipeline construction in sections III and IV. We then present the final, simplified pipeline for general users first, i.e., *logos* and scaffold-refinement diffusion in sections III and IV (detailed instructions and github repo follows).

I. Library construction

Ideally, a *de novo* designed binding protein would recognize each individual AA along an extended polymer chain with specific AA "pockets." However, even assembling pockets for a short 10 AA peptide leads to an exponential number of $20^{10} = 1.024^{13}$ designs. Based on the previous work(*7*), specificity to the unstructured peptides could be achieved by computationally redesigning pockets to recognize, say, 2-3 AA out of an 18 AA extended peptide target. Additionally, RFdiffusion showed extraordinary performance in adjusting the binding interface to achieve better affinity on helical peptide targets[8] and specificity on TNF protein targets [unpublished]. By combining both we established a possible solution: *de novo* design of a set of recognition pockets covering the alphabet of a representative 10 out of 20 AAs with the option to fine-tune the pocket's sensitivity later to increase the specificity. Twenty amino acids (AA) were grouped by their side-chain properties, such as charge (R/K/H, E/D), polarity (Q/N, T/S), torsion angle space (P, G), aromatics (W/Y/F), and hydrophobicity and size (M, I/L/V, C/A).

Scaffold generation

To construct a library of binding pockets specific for particular amino acids and dipeptides compatible with downstream fusion into single coherent structures (with extended peptide binding pockets), we adopted our previous superhelical matching approach(*11*) to design repeat proteins to bind repeating peptides as well as a broader range of peptide conformations. In this approach, repeating conformations of the peptide target sequence were generated, the superhelical parameters were determined,

repeat proteins with matching superhelical parameters were generated, and the two were docked together such that each repeat in the peptide makes identical interactions with a matching repeat in the designed protein. To achieve more general peptide recognition, we began by focusing on extended conformations, which are likely more highly populated for most protein sequences than the polyproline II conformation. As every other residue points in roughly the same direction in extended conformations, dipeptide repeats are more natural to target than tripeptide repeats (adjacent protein repeats do not need to have a large twist around the superhelical axis). We sampled the torsion angles of each of the two distinct residues in the dipeptide repeat from the extended region of the Ramachandran map (phi ranges [-150, -70], psi [-30, 150]), generated six repeat (twelve-residue) peptide conformations, computed their superhelical parameters, and generated helical repeat proteins with matching parameters. We then threaded the peptides through the matching repeat proteins, selecting for peptide-protein docks with bidentate hydrogen bonding interactions between protein sidechains and the peptide backbone. The remainder of the repeat protein sequence was then optimized using ProteinMPNN(*40*), and designed complexes with favorable Rosetta binding energies (DDG)(*41*, *42*) and AlphaFold2(*43*) (AF2) structure predictions matching the models were selected for experimental characterization (see Methods IV-5).

Base scaffold generation

To make the pocket templates programmable and experimental characterization modular, designed helical repeat (DHR) was chosen as base scaffolds to generate recognition pockets.

Each scaffold was constructed using a helix-loop-helix-loop pattern repeated at least four times. The helices typically consisted of 18 to 30 AAs, while the loops contained 3 to 5 AAs. The scaffold design process involved backbone design, sequence design, and structural prediction. The designs exhibited a wide range of twist (omega) between 0.5 and 1.2 radians, a radius ranging from 3 to 15 Å, and a rise between 0 and 20 Å to align with the peptides. The geometry of a repeat protein can be described by the super-helix radius, axial displacement, and twist.

The backbone design was achieved using Rosetta fragment assembly, guided by parameters and motifs. This involved 9,600 Monte Carlo fragment assembly steps, utilizing fragments from a non-redundant set of Protein Data Bank (PDB) structures. After inserting each fragment, the rigid-body transformation was extended to

subsequent repeats. The scoring for fragment assembly included Van der Waals interactions, packing, backbone dihedral angles, and residue-pair-transform (RPX) motifs. RPX motifs provided a rapid evaluation of full-atom hydrophobic packability of the backbone prior to side chain assignment. Post-design, backbones were screened for native-like features. Loops must have been within 0.4 Å of a naturally occurring loop or be rebuilt. Structures with helices deviating more than 0.14 Å were considered bent or kinked and were discarded. Structures with fewer than eight helices in contact were also filtered out. To select base proteins more curved or twisted than Armadillo repeat proteins (ArmRPs)(*44*, *45*) and tetratricopeptide repeats (TPRs)(*5*), the distance between the first and the last helix was required, as D_sml <= 15A, D_avg <= 25A (measured from Cα to Cα) to close up and surround the target.

Sequence design was performed on each filtered backbone using ProteinMPNN, with a customized weight on certain AAs: {"A": -0.15, "G": -0.15, "M": -0.35, "P": 0.15, "E": 0.1}. Sequence-level internal repeats were not forced in this round of backbone generation, as we reasoned that the sequence diversity would potentially help with gene synthesis and protein expression for repeat proteins. At last, structural prediction was conducted by AF2(*46*) with PLDDT > 90, Cα RMSD < 2 Å.

Hashing provides initial high-quality complex docks for ProteinMPNN

Following our previous work(*7*), a hash table was considered to maintain the highest resolution to store the privileged protein-peptide motif pairs of hydrogen bonding. To create hash tables that store pre-computed privileged side-chain–backbone bidentate interactions (to place the peptide backbone into the right 'docks'), we employed the previous hash database created for the polyproline II tri-peptide binders, but only focused on bidentate interactions.

To sample repeat peptides that align with the superhelical parameters of the designed helical repeats, we randomly generated sets of backbone torsion angles φ and ψ (e.g., φ ranges from [-150, -70], ψ ranges from [-30, 150] for mono-peptide, di-peptide, tri-peptide, tetra-peptide repeats). If any pair of φ and ψ angles yielded a Rosetta Ramachandran score above the −0.5 threshold, indicating potential steric clashes, we regenerated new pairs of angles until they met the Rosetta score criteria. Di-peptide

repeats were a focus of the initial templates, as less enumeration of sequence searching space was needed to cover amino acid alphabet while mono-peptide could introduce torsion angle limitation for future assembly. These torsion angles were then repetitively applied across the six repeats of the repeat peptide, and the superhelical parameters were calculated using the 3D coordinates of adjacent repeat units. Repeat peptides matching the superhelical parameters of any curated designed helical repeats were retained for docking.

For docking cognate repeat proteins and peptides with matching superhelical parameters, both were initially aligned to the z-axis based on their superhelical axes. A 2D grid search (involving rotation around and translation along the z-axis) was then performed to identify compatible positions of the repeat peptide within the binding groove of the repeat protein. Upon generating a reasonable dock without steric clashes, the relevant hash function iterated through potential peptide–protein interacting residue sets to compute hash keys. If a hash key was found in the hash table, the corresponding interacting side-chain identities and torsion angles were retrieved and applied to all equivalent positions in the docking conformation. Docked peptide–designed helical repeat pairs were saved for the interface design step if the peptide–designed helical repeat hydrogen-bond interactions were satisfied. Once accepted, this docked pair was stored in our initial base library logos-di-0, which produced a derivative library logos-di-1 with peptide docks perturbed through FlexPepDock (10 low-energy docks per template). We reasoned that the hash table approved initial peptide-designed helical repeat docks would enable the highest percentage of side-chain–backbone hydrogen bonding recovery followed by ProteinMPNN.

Pocket specialization

Base binders for di-peptide repeat recognition

For the first round of di-peptide repeat binder design, target candidates were generated through the enumeration of 19 x 18 AAs, excluding Cys (to avoid disulfide bond formation) and itself. Only combos containing at least one polar AA (i.e., Asp, Glu, His, Lys, Asn, Gln, Arg, Ser, Thr, Tyr, or Pro for its special Ramachadran constraint) were selected. Target (GA)n was intentionally added as an extreme case of a highly flexible target with the smallest side-chain combinations—which would then be forced to make

the smallest pockets and maximal backbone interactions. ProteinMPNN was applied with the same AA weights as above. All the designed complexes were validated by AF2 with a cutoff of PAE_interaction ≤10, PLDDT > 92 for the complex and PLDDT > 92, Cα RMSD < 2 Å for the binder monomer before experimental characterization. For the fully polar targets, AF2 was only used to judge the monomers, combined with a Rosetta scoring matrix as DDG < -50, contact_molecular_surface (CMS) > 500, BUNS (buried_unsatisfied_penalty) < 1.

In this work, the polar AAs were prioritized for a few reasons. 1) Polar targets can be particularly challenging due to the increased need for precise side-chain placements and hydrogen bonding networks; meanwhile, since it is significantly underrepresented in the PDB database used for deep learning training, it becomes an excellent option for rational computation design. 2) Polar interactions are presumably the driving force of target specificity due to the accurate side-chain placement from reason 1. 3) In order to reduce the vast search space of flexible backbone assemblies, we reasoned that designing a set of assemblies capable of precise polar targeting could be engineered to effectively target non-polar targets using deep learning resampling capabilities (due to reasons 1 and 2).

Therefore, we chose to first model a number of di-peptides (each one of the two AAs in an extended backbone conformation, occupying an individual pocket) in a repeat binding manner. This modeling provided experimental convenience (as we could easily enhance or diminish the binding energy contribution of any single pocket by changing its repeat number) and ensured the compatibility of later pocket assembly (all pockets being inserted on coherent helical bundles).

Backbone library through truncation and extension

After inspecting the first hits from round one of di-peptide repeat binders, helical fragments outside of the binding interface (with distance between Cα > 10 Å while Cβ > 8 Å) on the same helix were considered unnecessary. For each repeat, truncations were made symmetrically for each interface helix on the same spot, together with the matching sides on the buttressing helices. Loops were reconstituted with the exact same loop residues plus or minus (G) or (GS). After modification, ProteinMPNN was applied to produce 10 sequences for each base designed helical repeat. All 11 sequences per base designed helical repeat were sent to AF2. Designs with PLDDT > 92, Cα RMSD < 2 Å were selected to parametrically extend to five and six

repeats. Another round of MPNN-AF2 was applied to create the new di-peptide binding library.

Backbone library through parametric perturbation

To diversify the shapes of the binding pockets, we adapted the previously developed parametric repeat protein generation method for both symmetric and asymmetric perturbation with specified geometric parameters(*47*). For each helix in a base designed helical repeat, we performed a grid search of its six rigid-body degrees of freedom. The translation along any axis was limited to 2 Å, and the rotation around any axis was limited to 10 degrees. For asymmetric perturbation, only the designated helices were subjected to this protocol, and for symmetric perturbation, each of the sampled geometric transforms was propagated to the rest of the repeats in the designed helical repeat to maintain symmetry. We filtered the diversified designed helical repeats by removing the backbones that have high steric clashes (fa_rep>100), high intra-repeat inter-helix distance (distance between the centers of mass of the two helices in a repeat unit>12 Å), or less than 28% of the residues in a buried core.

Backbone library through motif diffusion

We manually picked the interacting residues surrounding the bidentate hydrogen bond donors to the target peptide, often four to nine residues per repeat. This is chosen with the hypothesis that the backbone hydrogen bonding is the prerequisite to lock the disordered target in an extended, random coil conformation. We input these binder motifs to diffusion, allowing variable lengths (from 0.75x to 1.25x compared to the parent backbone, normally with bigger variety on the N/C-terminal) of the protein backbone (fully noised atoms) to connect the motif residues, such that diffusion could reform and create new binding proteins scaffolded around the input binding motifs with variety. This improved and customized the shape complementarity between each binder and the individual target (while maintaining the key input motif residues, especially the ones needed for hydrogen bonded), improved and diversified the core packing of the binder (if needed), and sometimes increased the total number of binder residues contacting the peptide target.

I. Pocket assembly

Once we collected five di-peptides and ten amino acid binding pockets, we considered the next two milestones toward general sequence recognition to be, 1) efficiently assemble and shuffle the existing pockets; and 2) expand this assembly manner to arbitrary sequences beyond the pockets.

To do 1, we explored parametric assembly taking advantage of the modular nature of repeat proteins together with RFdiffusion to insert varied ‘spacers ’between the regular modules to create backbone diversity on both binders and targets. To do 2, we tested ‘derivative pockets ’(meaning, this pocket targets slightly newer amino acids) on sites of the existing pockets with deep learning-based protein design (i.e., ProteinMPNN, AF2, RFdiffusion).

Backbone pockets connecting through RFdiffusion

Based on the five di-peptide binders of representative amino acid motifs (LK), (RT), (YD), (PV), and (GA), we reasoned arbitrarily assembling them into 12-18 AA sequences would allow us to expand and cover a huge space of random target residue identities. This is judged in terms of side chain group size, overall charge distribution, hydrophobicity extent, aromatic properties, and backbone torsion angles.

To do this, we first enumerated the origami combos of target pattern generation, i.e., AAAAAA, AAABBB, AABBCC, AABCDD, ABCDEF, where each letter represented a unique existing di-peptide motif. Note that, to insert a single di-peptide motif into a continuous disordered fragment caused the recognition pocket before and after the pocket to fit into different backbone hydrogen bonding patterns. In other words, the assembled final interface distinguished (XX)n(PV)n and (XX)n(VP)n with a minor one-amino-acid pocket out of phase. Pockets with mutations on target (ABCDEF) were generated on the fly while assembling the target backbone with varied spacers as well to satisfy the backbone phi-psi angles better, such as (TR) to (TQ) in pc26.

We generated 36 synthetic assembled target sequences following the above pattern. Twenty target candidates with more diverse side-chain properties were chosen for binder design. For each target, we first generated the peptide conformation by copying and pasting their phi-psi angles from the individual amino acid motif pockets, with a

tolerance of 30° to connect and smooth the backbone transition between motifs. With exceptions, the ‘spacer’(s) between motif A and B were intentionally constructed with 0.5, 1.5, and 2.0 repeat units by Monte Carlo sampling a di-peptide backbone to fit in, instead of using 1 unit spacer to create peptide backbone diversity. Rosetta FastRelax was performed to maintain their lower energy states.

With the 20 target sequences in place with the calculated backbone ensembles, the interacting motifs from the di-peptide pockets were also placed accordingly using two strategies. 1) A small continuous helical chunk on the protein (with loopy regions occasionally included); or 2) individual interacting amino acids distributed on the protein. RFdiffusion was applied to connect these motifs to be a single chain A protein, while in the context of chain B target being present. As for the gap regions between the motifs (for RFdiffusion to connect and fill in), AA length ranged from 0.75x to 1.25x of the original AA length from the parent repeat-motif binder.

A thousand trajectories were carried out for each strategy of each design task. Two sequences per backbone were generated by ProteinMPNN. All sequences were then validated through complex predictions (AF2 with initial guess(*42*), PAE_interaction ≤10, PLDDT > 92 followed by AF multimer(*45*)) and monomer predictions (AF2 on chain A only).

Assemble to target arbitrary sequences beyond pockets

To go beyond existing pockets and motifs, we tested a set of eight arbitrary English words and human names ranging from eight to eighteen amino acids, i.e., DANIELSILVA, HIALIENFRIEND, DERRICKHICKS, DAVIDLIKESPEPTIDES, DAVIDITISTIME, WILLCHEN. To target arbitrary targets, we dissected them as individual amino acids instead of di-peptide motifs, and evaluated the assembled binders as a string of individual pockets. To do this, we first analyzed and compared the sequence identity of ‘the random words ’and ‘motif combos ’by BLOSUM62*(48)*, mapping each new target to the closest set of existing combos. This way, for two ‘unfamiliar targets,' HIALIENFRIEND and WILLCHEN (BLOSUM scores < -2.0), indicating there were no good matches in the current small set of libraries (~70 template pairs). We then assembled a set of 20 new templates following the above pocket assembly, with the purpose of targeting 70% of the target sequence (as we didn’t have full amino acid pockets by then).

For all the identified and newly assembled templates, we threaded the target into the template chain B backbone, performed chain B local backbone resampling through FlexPepDock(*49*), filtered out the backbone clashed docks and the docks losing more than 30% of the backbone hydrogen bonding. Survivors were then sent to customized ProteinMPNN for sequence design and customized AF2, as described below in detail in the ‘IDR threading ’section. Since this set of targets were new, we used several strategies of diffusion refinement on most of the designs as described below in detail in the ‘Refinement ’section.

Two-sided interface design

To increase the diversity of target sequences in fusion protein and multi-motif scaffolding binder generation, two-sided interface design was employed at the last stage. AF2 passing complexes were taken back, with both chain A and chain B redesigned through ProteinMPNN using the exact same protocol above of two iterations. Designs with DDG < -50 were sent to the same AF2 prediction. Out of this round at chain B sequence diversification (without intentionally modifying the backbones), 267 AF2 passing new targets were generated in silico, which could suggest the potential of this platform in general sequence recognition.

Logos - IDR threading

Supplementing the representative experimentally validated design complexes, we assembled an initial library of 340 templates. We reasoned that the set was broad enough to take most arbitrary disordered sequences, assuming that 1) at least one of the templates could map to at least one 8 AA window with a BLOSUM score > -1.0; and 2) a minimum of 70% of the 8 AA window was mapped (especially the polar amino acids), then the rest of the pocket and backbone could be easily optimized with deep learning. The whole pipeline is as mostly automated as three steps, 1) threading, 2) sequence design, and 3) prediction.

We tested this idea by arbitrarily targeting 21 broadly diverse native bioactive targets (small peptides and intrinsically disordered protein regions), where one can not choose or change any sequence identities on the target side. During this benchmark, we discovered our hypothesis held mostly true, with two new observations. First, the later tested targets performed better in general, both in silico and in vitro—If the target had a low BLOSUM score (<1.0) or the complex went through dramatic diffusion refinement (RMSD > 3.5) we recycled and supplemented the new "representative" validated binders back into the library. Second, adding the iteration of MPNN-AF2-MPNN-AF2 cycle with a customized AF2 package (see below) further improved the in silico metrics, possibly due to the local RMSD diversification guided by AF2 during design.

To further extend the capability of this pipeline, we implemented these findings into the base logos pipeline, intending to keep accepting new, diverse, challenging targets with help from AF2 and RFdiffusion. The final library presented in this paper contained 1,000 templates, picked from the experimentally tested designs with diverse binding modes, diverse unstructured targets, and diverse binder RMSD and sequences.

Threading

To input the target sequence information of any unstructured target as a string of amino acids a target fasta file was required. The sequence of interest was then threaded through our 'template library '(made of 1,000 diverse docks, each with a peptide backbone). The library contained four sub-libraries: "walle" (binds targets in mostly extended conformations with the massive amount of bidentate hydrogen bonds; extensively tested against dozens of targets throughput this work), "walle2" (supplemented experimentally validated designs throughput this work; similar to walle despite being less heavily tested), "strand" (binds targets in strand pairing

conformations), and “mini” (shorter binders designed to bind short targets; this is in a beta testing stage). For general users, we recommend prioritizing with the order of walle, walle2, strand, mini when limited numbers of designs can be experimentally tested.

This threading process followed the register of the target backbone and through each individual template to search for the potential binding windows (for the target fragment) and feasible binding modes (for the target-binder complex; later judged by AF2). A flag called --keep_bidentates could have been enabled to keep the bidentate hydrogen bonding donors fixed. After threading, the new complex dock was repacked and minimized[1]. FlexPepDock Rosetta package[2,3] was then used to enable local resampling of the binder and target backbone and sidechains, outputting 10 different complex docks per run. With the flag of --max_farep, only complexes with acceptably low energy (i.e., farep(protein + peptide_seq) - farep(protein + peptide_backbone) < 300.0 EU) were saved.

Cycle of Sequence Design

All accepted docks were sent to ProteinMPNN with a customized script, which contains empirical weights on certain amino acids. Two iterations of ProteinMPNN and FastRelax (to delicately adjust chain B backbones further), and binder SAP score(*41*) optimization were performed. By default, the top sequence with the lowest ProteinMPNN score from iteration one, and five sequences with the lowest ProteinMPNN score from iteration two were collected and sent to our customized AF2 package (see below). This filtering, in general, varies depending on the target difficulty level. Designs with PAE <= 5, RMSD <= 3, iPTM >= 0.83 (for easy targets, when > 7,000 designs would pass this criteria) or designs with PAE <= 10, RMSD <= 4, iPTM >= 0.79 (for hard targets, when differing from above) were selected to undergo one more round of the customized pipeline. Criteria can be further adjusted to individual targets and design campaigns as needed.

Predictions

After two cycles of sequence design and AF2 (which we call “prediction guided sequence design”), passing designs are collected to go through AlphaFold-multimer(*45*). Empirically, we found in the cases of highly polar targets (polarity >= 80%) and proline-rich targets (proline >= 30%, and/or more than one proline appears in the central of the sequence window), AlphaFold-multimer was more predictive than

AlphaFold initial. These filtering criteria were used for the final complex predictions: PAE <= 4-7, PLDDT >= 90 for AlphaFold-initial, and iPTM >= 0.8-0.9, PLDDT >= 90 for AlphaFold-multimer. Note that we found AF2 favored binding complexes with the disordered targets in secondary structural conformations (i.e., alpha-helical or beta-strand) and tended to give better scores. Therefore, we scored each sub-library separately with a more stringent criteria toward *Strand* (the sublibrary containing strand pairing binding modes; PAE <= 5; iPTM >= 0.88-0.9) than *Walle* (the sublibrary with diverse local extended binding modes, but less regular secondary structures; PAE <=5-9; iPTM >= 0.8-0.9). At last, AlphaFold-monomer was applied on the remaining designs on chain A only (binder) to select the final designs to order, with PLDDT >= 90. The designs with the best scores were selected to be experimentally tested in this work.

Illustration of the customized packages (MPNN, AF2)

MPNN

For sequence design, ProteinMPNN[4] was set up with a customized weight on certain amino acids: {"A": -0.15, "G": -0.15, "P": 0.15, "E": 0.1}. For polar targets (polarity > 80%), this set of weights has been experimented: {"A": -0.15, "G": -0.15, "M": -0.35, "P": 0.15, "E": 0.1, "Q": 0.3, "H": 0.3, "W": 0.1, "Y": 0.25}. Each round of MPNN was followed by Rosetta FastRelax[1] where one sequence with the lowest score from round one and five to ten (differ by target difficulty level) lowest score from round two were generated for each design.

Templated AF2-initial-multimer

For templated prediction, we developed a package called ‘AF2-initial-multimer. ’The AF2 initial guess (and target templating) method of Bennett et al.(*42*) was implemented into a local install of colabfold such that the initial guess could easily be utilized with the AF2-multimer models. We also added pyrosetta to our local install of colabfold to allow input and output of silent files which helps with file management when working with large numbers of designs. For each design from ProteinMPNN, we predicted whether the binder would bind its target peptide with the initial guess for each of the five multimer models using single sequence prediction. We averaged the metrics iPTM, binder_plddt, and interface_rmsd, from all five predictions, and utilized the averaged metrics for filtering purposes (see below).

MPNN-AF2 cycle

ProteinMPNN and AF2-multimer's initial guess filtering were iterated two times such that ProteinMPNN could assign a new sequence to the initial AF2 predictions when the predictions were close to the designs. AF2 predictions are able to improve the accuracy of the binder structure and local binding interface (including peptide structure) as has been demonstrated for previous de novo designs. By allowing ProteinMPNN to assign a new sequence to the more accurate backbone and docking configuration, we were able to improve the sequence structure agreement. After this, AF2 filtering was applied again, but with more stringent thresholds. The AF2 filtering metrics (averages of all five multimer models) for the first round were iPTM > 0.8, binder_plddt > 90 and interface_rmsd < 2.7. The AF2 filtering metrics (averages of all five multimer models) for the second round were iPTM > 0.88, binder_plddt > 92, and interface_rmsd < 1.0. For peptide targets that were particularly easy or difficult, the stringency of these filtering metrics were increased or decreased to control the number of designs moving to subsequent design or filtering steps. We grouped the passing designs with a clustering method based on binders 'amino acid sequence identities, with a dynamic AF2 metrics threshold, aiming to select the most structurally diverse set, with the top 10 scored designs from each cluster for AF2-multimer prediction.

Final AF2 prediction

Designs passing the previous AF2 initial guess filtering were now subjected to AF2 multimer single sequence predictions without initial guess using a local install of colabfold. The top <100 designs were selected from these predictions based on iPTM and subjected to visual inspection to select 3-70 designs for gene synthesis and experimental characterization.

Instructions and step-by-step guide:

The final pipeline of arbitrary IDR targeting consists of these steps (the code is attached below and through https://github.com/drhicks/Kejia_peptide_binders).

A computational pipeline to target arbitrary unstructured sequence fragments (4-30 amino acids) of intrinsically disordered proteins and peptides, with de novo designed binding proteins.

Prerequisites

- A Python environment with PyRosetta.
- Make sure silent_tools is in your PATH
- Download DL weights for AlphaFold, ProteinMPNN, and RF_Diffusion.
- Correct paths in MPNN scripts for your local installs.
- Update paths in path_to/threading/make_jobs.py to use your Python environment.
- Ensure path_to/threading/make_jobs.py has the correct path for path_to/threading/thread_peptide_sequence_new.py

Step-by-step guide from your working directory for this binder project.

0. Target selection

1. Cut your target region of interest into windows of 8-15 amino acids, save in fasta format targets.fasta
2. BLAST to search for the most unique windows in human proteome:

```
blastp -query targets.fasta -db human_proteome.fasta -out results.txt -outfmt "6 qseqid sseqid pident length evalue bitscore" -qcov_hsp_perc 70
```

3. Pick the ones with the least matches and save them in the file peptide.fasta

1. Threading

1. Make a directory for threading:

```
mkdir 1_threading
```

2. cd 1_threading
3. Make all jobs: make your target fasta file make your template list file
4. python path_to/threading/make_jobs.py path_to/peptide.fasta path_to/templates.list | sort -R > all_jobs

5. Split jobs into smaller sets:
6. split -l 3 all_jobs
7. Add all job sets to SLURM array list:
8. for i in x* ; do echo "bash $i" ; done > jobs
9. Submit array file jobs to SLURM:
10. path_to/threading/submit_jobs.sh
11. After jobs finish, collect all PDB outputs into a silent file:
12. silentfrompdbs path_to_pdbs/*pdb > threading.silent

2. MPNN

1. Back in your original working directory:

mkdir 2_mpnn

2. cd 2_mpnn
3. Run the initial MPNN (without relax; preferred):

path_to/job_creation/dev_mpnn_design_job_create -prefix mpnn -script path_to/mpnn_git_repo/design_scripts/killer_mpnn_interface_design.py -p cpu -t 12:00:00 -mem 5 -cpus 1 -conda path_to/env/mpnn_pyro -structs_per_job 100 -silent path_to/threading.silent -args "--num_seq_per_target 5 --max_out 5 --sampling_temp 0.1"

4. ./run_submit.sh
5. or (with apptainer)

path_to/job_creation/mpnn_design_job_create -prefix mpnn -script path_to/mpnn_git_repo/design_scripts/killer_mpnn_interface_design.py -p cpu -t 12:00:00 -mem 5 -cpus 1 -apptainer path_to/your_apptainer -structs_per_job 100 -silent path_to/threading.silent -args "--num_seq_per_target 5 --max_out 5 --sampling_temp 0.1"

6. ./run_submit.sh
7. In the paper, we have been routinely doing two rounds of sequence design (i.e., MPNN-relax-MPNN-relax) for many targets in an earlier time. To do this, one can simply run the above script twice with the flag --relax on. Set --num_seq_per_target 1 in the first run and --num_seq_per_target 5 in the second.
8. Concatenate all the silent files together:
9. cat mpnn_runs/*/*silent > mpnn_out.silent

3. AlphaFold Filtering and Refinement (AF2-initial-multimer)

1. Back in your original working directory:

mkdir 3_af2_im

2. cd 3_af2_im
3. Make array jobs:

path_to/job_creation/interfaceaf2create -prefix af2 -script path_to/colabfold_initial_guess/AlphaFold2_initial_guess_multimer.py -silent ../2_mpnn/mpnn_out.silent -gres "gpu:1" -apptainer

/home/drhicks1/scripts/Kejia_peptide_binders/colabfold_initial_guess/make_apptainer/colab_fold_ig.sif -structs_per_job 300 -p gpu-bf -t 06:00:00

4. ./run_submit.sh
5. Concatenate all the silent files together:
6. cat af2_runs/*/*silent > af2_out.silent
7. Create a scorefile:
8. silent_tools/silentscorefile af2_out.silent
9. Filter with sequence clustering and picking the top AlphaFold output/s per cluster after averaging 5 models:

python path_to/af2_filtering/average_af2_model_scores.py af2_out.sc > af2_out_averaged.sc
python path_to/af2_filtering/dynamic_filtering_by_group.py af2_out_averaged.sc af2_out.silent > cluster.log

10. column_number=$(head -1 cluster.log | tr '\t' '\n' | grep -n 'description' | cut -d: -f1); awk -v col=$column_number 'NR > 1 {print $col}' cluster.log | grep -oE '[a-zA-Z0-9_]+_af2mv3_[0-9]+' > tags

4. MPNN/AF2 cycle

1. Repeat MPNN step on filtered silent file.
2. Repeat AlphaFold IM and filtering.

5. Sequence Only AlphaFold Filtering and Refinement (potentially optional)

1. Make fasta file.
2. silentsequence path_to/af2_out_filtered.silent | awk '{print ">"$3"\n"$1":"$2}' > colabfold_input.fasta
3. Run colabfold.
4. /home/drhicks1/scripts/Kejia_peptide_binders/colabfold_initial_guess/make_apptainer/colab_fold_ig.sif AlphaFold2_jupyter-batch_hack_new_v2.py --fasta colabfold_input.fasta --num_recycles 10
5. Concatenate all the silent files together:
6. cat af2_runs/*/*silent > af2_out.silent
7. Create a scorefile:
8. silent_tools/silentscorefile af2_out.silent
9. Filter with sequence clustering and picking the top AlphaFold output/s per cluster after averaging 5 models:

python path_to/af2_filtering/average_af2_model_scores.py af2_out.sc > af2_out_averaged.sc

10. python path_to/af2_filtering/dynamic_filtering_by_group.py af2_out_averaged.sc af2_out.silent --not_initial_guess

Additional Steps

- Other filtering steps as desired such as af2_filtering/rosetta_min_ddg.py and visual inspection to order.
- Incorporate motif diffusion or partial diffusion as needed

Notes

- You may choose to run MPNN with Rosetta relax, but this is slow and questionably useful. If you do, add the flag --relax.
- If you run relax or minimization methods that can perturb the rigid body and/or binder/target backbones, you could run a second MPNN on the output of the first to potentially design a better sequence. However, the current preference is to go straight to AlphaFold filtering/refinement.
- Diffusion can be run at various steps such as:
    1. After 1 or 2 rounds of mpnn/af2: if not enough designs (< 70) are passing the final filtering criteria, in which case you will want to repeat the two cycles of mpnn/af2 on the output from diffusion.
    2. On the final designs before ordering: if enough designs (>= 70) are passing the final filtering criteria, but one may want to order on chips and/or include arbitrary refined designs in initial test, in which case you can repeat either the one cycle or two cycles of mpnn/af2 on the output from diffusion. Depending on available computation resources and chip quota.
    3. On the initial hits after experimental screening and characterization, in which case you will want to repeat the two cycles of mpnn/af2 on the output from diffusion.
- In general the pipeline works most times without the use of diffusion, however, intelligent use of diffusion can increase in silico success rates for difficult targets and potentially improve the affinity and specificity of characterized binders.
- There are many knobs that can be tuned and variations of the pipeline that can be run depending on the ease or difficulty of individual targets.
- Diverse plus good in silico designs generated by the pipeline (regardless of experimental characterization) can be added into the templates to continue building new diverse binding modes for the future.

Motif Diffusion

bash make_motifd_jobs
bash motif_d_jobs
Submit jobs

Partial DIffusion

If partial diffusion, refer to the normal published partial diffusion with small partial_T.

Refinement

Pocket recognition and pocket assembly enabled refinement of precise interactions with individual amino acids in a sequence-specific manner. This was critical for challenging polar or charged targets, or targets that would not adopt regular secondary structures when bound.

To avoid running into a theoretical modular assembly limit introduced from a finite number of assemblies and an infinite number of possible targets, we developed a refinement package based on RFdiffusion(*11*) called 'scaffold-refined diffusion. 'This aimed to provide adequate high-resolution refinement and perturbations to the 'mismatches 'between pockets, spacers, and protein backbones even when the overall match was a coarse-grained fit, i.e., when accepting a new enough target in terms of sequence identities. This way, we maintained the high-efficiency engineering for targeting disordered regions with low computation resources (1-3 computation days, 1,500 CPU hours, 500 GPU hours per target) and minimum experimental cost for most arbitrary disordered targets (3-70, on average 36 tested designs per target). Meanwhile, we provided an additional option to surpass the theoretical limitation (potentially with a slightly higher computational/experimental cost).

Scaffold-refined Diffusion

If the design did not pass the above complex prediction criteria, or if a larger number of tested designs were preferred, scaffold-refined diffusion was developed to optimize the customized fits between the less ideal interacting pockets and the less ideal binder backbone. To do this, a fair amount of the original binding interaction and/or binding motifs were kept to maintain the advantage of our general platform. Therefore, partial diffusion[8] with small steps (unless for the purposes of new library construction) and multi-motif-constrained diffusion (motif diffusion) were developed for this purpose. For the work in this paper, a customized build of RFdiffusion was implemented.

Partial diffusion (one-sided)

As described in other cases, RFdiffusion was modified to allow the input structure to be noised only to a user-specified time step rather than completing the full noising schedule. Consequently, the starting point of the denoising trajectory retained information about the input distribution, leading to denoised structures that are structurally similar to the original input. In our cases, the time step was set to be lower to maintain as much structural similarity as possible as the high design resolution of

hydrogen bonding interactions (especially to the polar amino acids) were reasoned critical. Therefore, 10-18 noising timesteps (specifically, 10, 12, 15, 18) out of a total of 50 in the noising/denoising scheme were chosen for the work in this paper. Two hundred to three thousand partially diffused designs were generated for each target parameter.

The new backbones went through ProteinMPNN sequence design and AF2 predictions as described above. The designs were then filtered using the same criteria. It was noticed that partial diffusion tended to increase the number of overall passing designs, while sometimes the original hydrogen bonding networks (especially the bidentate hydrogen bonds to the target backbone) were broken without being caught by AF2. Therefore, we implemented another filter 'buns '(buried unsatisfied hydrogen bonds)(*50*) <=1 to explicitly count the unsatisfied heavy atoms. In some cases, overall correlated with target polarity, this step would filter out 50-80% of designs. Partial diffusion seemed to increase the wet lab success rate while being inconclusive as to the affinity increase unless tested on a much larger number of designs (>1,000 designs, unpublished data).

Partial diffusion (two-sided for library construction)

Unlike one-sided directional partial diffusion, which only diversifies the conformation of the binder while keeping the target unchanged, two-sided partial diffusion enabled simultaneous conformational changes in both the target and the binder.

For the input designs, we applied 10-25 noising timesteps out of a total of 50 in the noising scheme followed by denoising. This process generated approximately 2,000-10,000 partially diffused designs for each target. The new backbones went through ProteinMPNN sequence design and AF2 predictions as described above. The designs were then filtered using the same criteria. It was noticed that two-sided partial diffusion tended to increase the number of complex passing designs and slightly decrease the number of monomer passing designs. We did not see a direct correlation with the following wet lab success rates as targets varied.

Motif diffusion

We utilized a custom pyrosetta script to identify binding motifs; defined as any residue making atomic contacts (heavy atoms < 4.5 Å) to atoms in the target peptide. We input these binder motifs into diffusion, allowing variable lengths (from 0.75x to 1.25x, normally with bigger variety on the N/C-terminal) of protein backbone (fully noised atoms) to connect the motif residues, such that diffusion could reform and create new binding proteins scaffolded around the input binding motifs with variety. This improved and customized the shape complementarity between each binder and the individual

target, while maintaining the key input motif residues, especially the ones that are hydrogen bonded; improved and diversified the core packing of the binder (if needed); and sometimes increased the total number of binder residues contacting the peptide target. Motif diffusion has seen consistent improvement in both in silico and in vitro binding metrics in most of the cases we tested.

RFdiffusion

In this work, we were using accelerated denoising schedules in RFDiffusion that make fewer calls to the underlying RosettaFold2 (RF2) module. We implemented twpst which is partial diffusion with only one frame. We additionally used a technique to skip even more calls to RF2: Instead of adjusting the denoising schedule (which seems to have a limit of no fewer than 40 RF2 calls while maintaining quality), we repeatedly seeded the Px0 (re-noised to the current t) as the current xt structure allowing us to achieve schedules without only 15 RF2 calls.

Experimental Methods

Gene Construction of Designed Binders

The designed protein sequences were optimized for efficient expression in *E. coli*. linear DNA fragments encoding these design sequences were obtained (eBlocks, Integrated DNA Technologies) and included overhangs compatible with Golden Gate cloning into the LM670 vector (Addgene #191552) for *E. coli* protein expression. The LM670 vector is a modified expression system featuring a Kanamycin resistance gene, a ccdB lethal gene flanked by BsaI cut sites, and a C-terminal hexahistidine (6xHis) tag. Peptide genes were purchased as fusion proteins to either the C terminus of sfGFP or the N terminus of a GB1−AviTag−His6x construct separated by (PAS) linker or (GGSGSG) linker. In the cases specified in Fig. S3 and Fig. S7, the designed protein sequences were purchased as fusion proteins to an AviTag-POI-His6x construct separated by (GGSGSG) linker, while targeting peptides in the C terminus of sfGFP.

Protein production and purification (small scale and medium scale)

Linear gene fragments encoding the binder design sequences were cloned into the LM670 vector using Golden Gate assembly. These subcloning reactions were performed in 96-well PCR plates with a 4 µL reaction volume. Following this, 1 µL of the reaction mixture was transformed into chemically competent *E. coli* BL21 (DE3) cells.

After a 1-hour recovery period in 100 µL of SOC medium, the transformed cell suspensions were transferred directly into a 96-deep well plate containing 900 µL of LB media supplemented with Kanamycin. After overnight incubation at 37°C, 100 µL of the growth culture was inoculated into 96-deep well plates containing 900 µL of auto-induction media (autoclaved TBII media with Kanamycin, 2 mM MgSO4, 1X 5052). The cultures were then expressed overnight at room temperature.

Cells were harvested by centrifugation at 4000 x g for 15 minutes. The bacterial pellets were lysed in 100 µL of lysis buffer (1X BugBuster (Millipore #70921-4), 0.01 mg/mL DNase, 1 Pierce protease inhibitor tablet per 50 mL lysis) for 30 minutes on shaker, 220 RPM. The lysates were spun down by centrifugation at 4000 x g for 10 minutes, followed by purification using Ni-charged MagBeads (GenScript #L00295). The wash

buffer contained 25 mM Tris pH 8.0, 300 mM NaCl, and 10 mM Imidazole, while the elution buffer contained 25 mM Tris pH 8.0, 300 mM NaCl, and 500 mM Imidazole. 250 µL of wash buffer was used to wash twice while 120 µL of elution buffer was used to collect eluted proteins. Filtered elutions were then submitted to HPLC, S200. For samples showing major monomeric peaks, protein concentrations were determined by measuring absorbance at 280 nm with a NanoDrop spectrophotometer (Thermo Scientific), using extinction coefficients and molecular weights calculated from their amino acid sequences.

For further validation, proteins were expressed at a 50 mL scale using autoinduction for approximately 24 hours. During the first 6 hours, cultures were grown at 37°C, followed by incubation at 22°C for the remaining time. Cultures were harvested by centrifugation at 4000 x g for 10 minutes and resuspended in approximately 20 mL of lysis buffer (25 mM Tris-HCl, 150 mM NaCl, 0.1 mg/mL lysozyme, 0.01 mg/mL DNase, 1 mM PMSF, and 1 Pierce protease inhibitor tablet per 50 mL culture). Sonication was performed with a four-prong head for a total of 5 minutes, with 10-second on/off pulses at 70% amplitude. The resulting lysate was clarified by centrifugation at 14000 x g for 30 minutes. The lysate supernatants were applied directly to a 1 mL bed of Ni-NTA agarose resin equilibrated with a binding buffer. After sample application and flow-through, the resin was thoroughly washed two times, and samples were eluted with an elution buffer containing 500 mM imidazole. Post-elution, protein samples were filtered and injected into an Akta Pure system equipped with an autosampler, using a Superdex S200 Increase 10/300 GL column at room temperature. The SEC running buffer consisted of either 25 mM Tris-HCl, 150 mM NaCl, pH 8.0 or 1x PBS. Protein concentrations were determined by measuring absorbance at 280 nm with a NanoDrop spectrophotometer (Thermo Scientific), using extinction coefficients and molecular weights calculated from their amino acid sequences.

BLI assays for initial screening and affinity measurement

As for quantitatively screening all designs, Bio-Layer Interferometry (BLI) was employed (detailed method described below). These samples were screened by BLI with concentrations ranging from 200 nM to 2 µM. Designs exhibiting obvious binding signals (>= 0.2 AU) were further analyzed by BLI through titration (with double controls).

BLI experiments were conducted using an Octet Red96 (ForteBio) instrument with streptavidin-coated tips (Sartorius Item no. 18-5019). The assay buffer was 1X HBS-EP+ (Cytiva BR100669) supplemented with 0.1-0.2% w/v bovine serum albumin and 0.1% sucrose. Each design was initially tested for non-specific binding against unloaded

tips. Biotinylated target peptides (50-400 nM) were loaded onto the tips for 50-300 seconds to reach their 1/3 to 2/3 maximal loading signals (conducted by a loading test in advance), followed by a 60-second baseline measurement. After loading, all runs underwent a 60-second baseline, 100-800 seconds association, and 100-700 seconds dissociation. Baseline measurements from unloaded tips were subtracted from their corresponding loaded tip measurements. Twofold or Threefold serial dilutions were performed during the titration, with double controls in each run (i.e., one as Octet buffer to the loaded tip for signal subtraction, one as the highest tested protein concentration to the non-loaded tip to check for non-specific binding). For some runs toward the same target, streptavidin tips were regenerated five times in HCl pH 1.0 buffer for three 5-second exposures after each single run. For each individual titration run, the target and binder concentrations, association and dissociation times were adjusted as needed. Steady-state and global kinetic fits were carried out using the manufacturer's software, Data Analysis 9.1, based on the assumption of a 1:1 binding model.

BLI all by all orthogonality matrix

BLI experiments were performed as described above for individual binding measurements. Here, each of the 20 biotinylated-targets was loaded onto the streptavidin-coated tips again to reach their 1/3 to 2/3 maximal loading signals. Each target was tested against its cognate designed binder and other 19 noncognate binder individually at titration ranges from 1 uM to 4 nM. For the same target, streptavidin tips were regenerated in HCl pH 1.0 buffer for three 5-second exposures after each single run. Titration experiments were conducted at 25°C with continuous rotation at 1,000g. Association and dissociation with various binders were allowed for 200-1,000 seconds for each step. All binding signals at 1 uM were normalized by their cognate pairs and converted for heatmap plotting.

NanoBiT assays for initial binding screen, binding measurements and all by all orthogonality matrix

To qualitatively screen designs, the split-luciferase assay (nanoBiT) was carried out using the Nano-Glo Luciferase Assay System (Promega). The coding sequence for the small-BiT was fused to the gene encoding the peptide binders, while the large-BiT coding sequence was fused to the gene encoding the target peptide. The BiT-fused proteins and peptides were expressed and purified following the same protocol as above.

All assays were conducted in a buffer containing 20 mM sodium phosphate, 100 mM NaCl, pH 7.4, and 0.05% v/v Tween 20. Reactions were set up in 96-well plates

(Corning, cat. no. 3686) with Nano-Glo substrate (Promega, cat. no. N1130), diluted 500× for endpoint measurements. Luminescence signals were recorded using a Synergy Neo2 plate reader (BioTek).

Initial binding assays were performed with one component (i.e., the target peptide) held constant at 1 nM or 0.5 nM while the other protein (i.e., the designed binder) normally around 500 nM to 2 uM. The plates were incubated overnight at room temperature before adding the substrate and immediately measuring luminescence. Constructs showing obvious binding (high luminescence signals) at relatively lower concentrations were identified as initial hits for Octet characterization to further determine the binding affinity.

During the all-by-all (18x18) orthogonality titrations, all the target peptides were held constant at [lgBiT]=1nM, while the designed binders were titrated at threefold dilutions of [smBiT]=333nM, 111nM, 33.3nM, 11.1nM. The plates were incubated overnight at room temperature before adding the substrate and immediately measuring luminescence. Each experiment was repeated twice, the averaged luminescence signals were used for plotting.

Protein Purification for Crystallography

Constructs were transformed into LEMO21 or NEB BL21(DE3) *E. coli* and expressed as 0.5 L cultures in 2 L flasks. Proteins were produced using Studier's M2 autoinduction media containing 50 µg/mL kanamycin. Pre-cultures were grown at 37°C for 4 hours, then shifted to 22°C for 14 hours before being used to inoculate the main cultures with 10 mL of pre-culture. After growth, cells were harvested by centrifugation at 4000 x g for 10 minutes, and the supernatant was discarded. The cell pellets were resuspended in 40 mL of lysis buffer (100 mM Tris-HCl pH 8, 100 mM NaCl, 400 mM imidazole, 1 mM PMSF, 1 mM DNase). Cells were lysed using a Microfluidics M-100P microfluidizer at 18,000 psi, and the lysate was clarified by centrifugation at 14,000 x g for 30 minutes.

His-tagged proteins were bound to 8 mL of Ni-NTA resin (Qiagen) using gravity flow. The bound proteins were washed with 10 mL of lysis buffer followed by 30 mL of high salt wash buffer (25 mM Tris-HCl pH 8, 1 M NaCl, 20 mM imidazole), and then with 10 mL of SNAC cleavage buffer (100 mM CHES, 100 mM acetone oxime, 150 mM NaCl, 500 mM GnCl, pH 8.6). To initiate cleavage, 40 mL of SNAC cleavage buffer and 80 µL of 1 M $NiCl_2$ were added, and the columns were sealed and shaken on a nutator for 12 hours. After cleavage, the flowthrough was collected and concentrated prior to further purification using SEC/FPLC on a HiLoad 20/600 Superdex 75 pg column in TBS (20 mM Tris pH 8.0, 150 mM NaCl), with 14 mL fractions collected between 100 and 290 mL.

TAMRA Peptide Synthesis

Peptides were synthesized in-house using a CEM Liberty Blue microwave synthesizer. All amino acids were sourced from P3 Biosystems. Oxyma Pure was obtained from CEM, DIC from Oakwood Chemical, and diisopropyl ethylamine (DIEA) and piperidine from Sigma-Aldrich. Dimethylformamide (DMF) was procured from Fisher Scientific and pre-treated with an Aldraamine trapping pack. The 5(6)-carboxytetramethylrhodamine carboxylic acid (5(6)-TAMRA) was purchased from Novabiochem.

The synthesis was conducted on a 0.1 mmol scale using CEM Cl-MPA resin. Each amino acid, at a concentration of five equivalents, was activated with 0.1 M Oxyma and 2% (v/v) DIEA in DMF, combined with 15.4% (v/v) DIC. Coupling was performed twice on the resin for 2 minutes per coupling with microwave irradiation. For TAMRA-labeled peptides, the peptides were washed with DMF post-synthesis, then incubated for 3 hours with 5(6)-TAMRA carboxylic acid (3 equivalents), HATU (3 equivalents), and DIEA (5 equivalents) in DMF. After incubation, the peptides were washed with DMF (three times) and DCM (three times) in preparation for global deprotection.

Global deprotection was achieved using a mixture of TFA/water/TIPS/2,2'-(ethylenedioxy) diethanethiol (92.5:2.5:2.5:2.5) for 3 hours. This deprotection mixture was concentrated in vacuo to 2-3 mL, then precipitated in 30 mL of ice-cold ethyl ether, centrifuged, and decanted. The peptide was washed twice more with fresh ether and dried under nitrogen to yield the crude peptide, which was then purified by high-pressure liquid chromatography (HPLC). The crude peptide was dissolved in a minimal amount of ACN and water to ensure complete solubility. Purification was performed on a Zorbax Stablebond C18 (9.4 x 250 mm, 5 µm) column using an Agilent 1260 Infinity HPLC with a linear gradient of water (0.1% TFA) and increasing ACN (0.1% TFA). UV signals were monitored at 214 nm and all peaks were collected.

Peak masses were verified using an Agilent G6230B LC-MS, and purity was assessed with a C18 column (Higgins Analytical PROTO 300 C18, 10 µm, 10 x 250 mm) on an analytical Agilent 1260 Infinity II HPLC.

Biotinylated Peptide Synthesis

All Fmoc-protected amino acids were sourced from P3 Bio. The synthesized biotinylated peptides were modified at the N terminus with biotin-Ahx-GGGS, using biotin-Ahx building blocks also obtained from P3 Bio. Oxyma was procured from CEM, and DIC was acquired from Oakwood Chemicals. Dimethylformamide (DMF) was purchased from Fisher Scientific and treated with an AldraAmine trapping pack from Sigma-Aldrich

prior to use. Piperidine was also purchased from Sigma-Aldrich. Cl-TCP(Cl) resins were obtained from CEM.

Peptides were synthesized on a 0.1 mmol scale using microwave-assisted solid-phase peptide synthesis on a CEM Liberty Blue system. After synthesis, the peptides were cleaved using a cocktail of trifluoroacetic acid (TFA), TIPS, water, and DODT in a 92.5:2.5:2.5:2.5 ratio. The cleavage solution was concentrated under vacuum, precipitated into cold ether, and centrifuged. The resulting pellet was washed and centrifuged again with ether (twice), dried under nitrogen, resuspended in water and acetonitrile (ACN), and purified by reverse-phase high-performance liquid chromatography (RP-HPLC) using an Agilent 1260 Infinity semi-preparative system with a gradient from 20% to 70% over 15 minutes (A: H2O with 0.1% TFA; B: ACN with 0.1% TFA). The purified peptide fractions were combined, lyophilized, and weighed in a tared scintillation vial.

Depending on their isoelectric points, lyophilized peptides were solubilized in buffers containing either 100 mM Tris pH 8.0 or 100 mM MES pH 6.5 and stored at −20°C.

Fluorescence Polarization

All fluorescence polarization (FP) measurements were conducted at 25°C in 96-well plates (Corning 3686) using a Synergy Neo2 plate reader equipped with a 530/590 nm filter cube. Peptide targets were synthesized with N-terminal tetramethylrhodamine labels. The buffer for all FP measurements consisted of 20 mM Tris-HCl, 100 mM NaCl, and 0.05% v/v TWEEN20, adjusted to pH 8. Titrations were performed in a 96-well format with four replicates per plate, comprising either 24 or 48 data points per titration (including 23 or 47 steps of two-fold serial dilution of designed binders in the presence of a TAMRA-labeled peptide at a constant concentration between 0.1 nM and 1 nM), with a final sample volume of 80 µL per well. To ensure complete equilibration, the titration plates were incubated from 3 hours to overnight at room temperature before measurements were taken.

The polarization signal SSS (as calculated by the Neo2 software) was fitted to the equation: $S = S_0 + S_1 \cdot f_{AB}$

where:

$$f_{AB} = \frac{1}{2B_{\text{tot}}}\left(A_{\text{tot}} + B_{\text{tot}} + K_D - \sqrt{(A_{\text{tot}} + B_{\text{tot}} + K_D)^2 - 4 \cdot A_{\text{tot}} \cdot B_{\text{tot}}}\right)$$

Here, f_{AB} represents the fraction of the peptide that is bound, A_{tot} is the absolute concentration of the hinge, B_{tot} is the absolute concentration of the peptide, S_0 is the baseline polarization of the free peptide, and S_1 is the change in polarization upon complex formation.

The fitting process was performed using the scipy.optimize.curve_fit function in Python. The uncertainties for K_D values were calculated as standard deviation errors derived from the covariance matrix of the fits. In instances where the fitted K_D was lower than the concentration of the labeled peptide B_{tot}, the K_D was reported as $K_D < B_{tot}$.

Circular dichroism (CD)

Circular dichroism spectra were measured with an AVIV Model 420 DC or Jasco J-1500 circular dichroism spectrometer. Samples were 0.25 mg ml^{-1} in TBS (25 mM Tris pH 8.0 and 150 mM NaCl), and a 1 mm path-length cuvette was used. The circular dichroism signal was converted to mean residue ellipticity by dividing the raw spectra by N × C × L × 10, in which N is the number of residues, C is the concentration of protein and L is the path length (0.1 cm).

Cell culture and transfection for colocalization assay

HeLa (CCL-2) cells were purchased from ATCC and were cultured in DMEM, high glucose (GIBCO) supplemented with 10% Fetal Bovine Serum (Hyclone) and 1% penicillin-streptomycin (GIBCO). Cells were grown with 5% CO_2 at 37°C.

Transfection of HeLa cells was performed using Lipofectamine 3000 (ThermoFisher, L3000015) following the manufacturer's protocol. A day before transfection, 1 x 10^4 cells were seeded on a glass bottom 96-well plate. 200 ng plasmid (1:1 mix of plasmid encoding binder-GFP and plasmid encoding peptide-mCherry) was transfected. 24 hours after transfection, cells were imaged using IN Cell Analyzer 2200 (GE). Images were processed using ImageJ.

Two off-targets to DYNA (wildtype sequence YGGFLRRIRPKLKWDNQ) were selected based on two Alanine point mutations to the wildtype (YGGFLRRIR**AA**LKWDNQ), as well as homologous sequence from human protein database (IGGFMVRQRKSHTRTK).

Cell surface staining assay

To visualize binder association with MSLN, HPAC cell lines (High MSLN expression) and MCF7 cell lines (No MSLN expression) were plated to confluence in a 96-well glass bottom plate (CellVis). Immediately before incubation with the binder, the growth media is aspirated completely. Purified MSLN binders tagged with GFP, produced following the same protein expression and purification method above, were normalized to the same concentration in 1X PBS before transfer into each well containing the MSLN/HPAC cells. The binders are allowed to incubate with the cell for 30 minutes at room temperature. Following incubation, the protein solution is aspirated and the cells are washed with 200 uL 1X DPBS three times before visualization on the INCell Analyzer 2500 HS (Cytiva) at 60x magnification through the green fluorescence channel. Binders to MSLN are expected to show a distinct green outline along the cell membrane.

Individual design characterizations

Purification of FAM21 binders

Plasmids encoding FAM21 binders tagged with an N-terminal PC tag and C-terminal 10x his tag were transformed into BL21(DE3) *E. coli*. Bacteria were cultured at 37°C in 2xTY medium to an optical density at 600 nm of 0.6 and then induced using 0.5 mM isopropylthiogalactoside (IPTG) at 20°C for at least 16 h. Cells were pelleted using centrifugation. Unless stated otherwise, all purification steps were performed at 4°C. Cell pellets were resuspended in lysis buffer (25 mM HEPES pH 7.2, 500 mM NaCl, 10% glycerol, 1 mM $CaCl_2$, 100 µg/ml lysozyme, 1 mM DTT, and cOmplete protease inhibitor cocktail tablets EDTA free (Roche)) and lysed by sonication. Lysates were spun for 1 hour at 18,000 rpm and the supernatant containing soluble proteins was incubated with pre-equilibrated Protein C affinity resin for 1.5 hours. The resin was then packed into an empty column (Bio-Rad), washed with high salt wash buffer (25 mM HEPES pH 7.2, 500 mM NaCl, 10% glycerol) and then with low salt wash buffer (25 mM HEPES pH 7.2, 150 mM NaCl, 10% glycerol). Binders were eluted with an elution buffer (25 mM HEPES pH 7.2, 150 mM NaCl, 10% glycerol, 5 mM EGTA). Binders were then concentrated (Amicon® Ultra Centrifugal Filter, 10 kDa MWCO) and dialysed overnight using Slide-A-Lyzer Mini dialysis device, 3.5K MWCO (ThermoFischer) into 25 mM HEPES pH 7.2, 150 mM NaCl, 10% glycerol. Proteins were then further purified by their His-tag using TALON resin. Proteins were incubated with TALON resin (Cytiva) for 30 mins with rolling and then beads were poured into an empty Bio-Rad column and extensively washed with 25 mM HEPES pH 7.2, 150 mM NaCl, 10% glycerol. Proteins

were eluted with 25 mM HEPES pH 7.2, 150 mM NaCl, 10% glycerol, 300 mM imidazole. Proteins were concentrated (Amicon® Ultra Centrifugal Filter, 10 kDa MWCO) and dialysed overnight using Slide-A-Lyzer Mini dialysis device, 3.5K MWCO (ThermoFischer) into 25 mM HEPES pH 7.2, 150 mM NaCl, 10% glycerol. Purified proteins were stored at 4°C.

Immunoprecipitation with FAM21 binders

Immunoprecipitation steps were performed at 4°C unless otherwise stated. 20 µg of PC tagged FAM21 binder (KFAM binder) was diluted into 25 mM HEPES pH 7.2, 150 mM NaCl, 10% glycerol, 1 mM $CaCl_2$ and incubated with 20 µl pre-equilibrated Protein C affinity resin for 2 hours. For a negative control, Protein C affinity resin was incubated with buffer only. Unbound binder was removed by washing the resin three times with 25 mM HEPES pH 7.2, 150 mM NaCl, 10% glycerol, 1mM $CaCl_2$. HEK293 cells were lysed in 25 mM HEPES pH 7.2, 150 mM NaCl, 10% glycerol, 0.5% NP40, 1 mM $CaCl_2$ and cOmplete protease inhibitor cocktail tablets EDTA free (Roche). Lysate was spun on a tabletop centrifuge at 14,000 rpm for 10 minutes at 4°C. Equal volumes of supernatant were added to the binder-Protein C resin, an input was taken for analysis and then binder:Protein C resin:HEK293 lysate was incubated for 16 hours. Resin was then gently pelleted (4,000 rpm, 1 min) and washed three times with 25 mM HEPES pH7.2, 150 mM NaCl, 10% glycerol. After removing the final wash buffer, protein was eluted with 25 mM HEPES pH7.2, 150 mM NaCl, 10% glycerol, 5mM EGTA. Beads were incubated on ice for 1 hour for the elution. Elutions were collected and analysed by Western blot and Coomassie staining.

Inputs and elution samples were run on 4–12% Bis-Tris gels (Invitrogen NP0323BOX) in NuPAGE MES SDS running buffer. All Blue Precision Plus Protein Prestained Standard (Bio-Rad) was loaded as a molecular weight ladder. Proteins were transferred to a PVDF membrane (Immobilion-FL, pore size 0.45 µm, Millipore, catalogue number IPFL00010). Membrane was activated in methanol (Sigma) for 5 min. Transfer occurred in transfer buffer (25 mM Tris, 192 mM glycine, 10% methanol) at 100 V for 80 min. Membranes were blocked in 5% (w/v) milk in TBS-TWEEN (10 mM Tris-HCl, 120 mM NaCl and 1% (w/v) TWEEN20, pH 7.4) for 30 min at room temperature with gentle shaking. Mouse anti-Strumpellin (SantaCruz, sc-377146), Rabbit anti-FAM21 (ABT79, Sigma-Aldrich), Rabbit anti-SWIP (51101-1-AP, Proteintech), Rabbit anti-CCDC53 (Merck, ABT69) were diluted in 1% (w/v) BSA in TBS-TWEEN and incubated with the membrane overnight at 4°C with gentle shaking. The membrane was washed three times in TBS-TWEEN then incubated with goat anti-mouse Alexa 647 (Invitrogen, A-21237) or donkey anti-Rabbit Alexa 488 (Invitrogen, A-21206) for 1 hour at room temperature with gentle shaking. Mouse anti-β-tubulin (DSHB, E7) was directly labelled

with AlexaFluor 488 (Derivery, 2015) and diluted in 1% (w/v) BSA in TBS-TWEEN and incubated with the membrane overnight at 4°C with gentle shaking. Membranes were washed three times with TBS-TWEEN. Membranes were imaged using a Typhoon scanner. Alternatively, the same samples were analyzed using 4–12% Bis-Tris gels (Invitrogen NP0323BOX) and stained with InstantBlue Coomassie stain (Sigma ISB1L) for total protein staining. Coomassie gels were imaged using a ChemiDoc system (BioRad).

Generation of stable U2OS cell lines expressing binders

Human U-2 OS (also known as U-2 OS) cells were purchased from ATCC. Homozygous PER2-HaloTag knock-in human U2OS cells were generated using CRISPRCas9 mediated homology-directed repair as described in Beale et al., 2023*(51)*. Stable, polyclonal cell lines expressing the binder of interest were generated in the U2OS PER2-HaloTag knock-in background. To this end, the target plasmid was first linearised via restriction enzyme digestion and cleaned up using a PCR clean-up kit (T1030L, NEB). The cells were transfected with the target plasmid using Lipofectamine 3000 (L3000001, Thermo Fisher Scientific), according to the manufacturer's protocol. 48 hours later, selection for stable expression commenced by maintaining the cells in the standard cell culture medium: DMEM (25mM glucose, 1mM sodium pyruvate, 44mM sodium bicarbonate; 41966, Gibco) supplemented with 10% HyClone FetalCloneII serum (SH30066.03IH25-40, GE Healthcare) and penicillin/streptomycin (100 IU/ 100 µg/ml respectively), supplemented with 4 µg/ml puromycin (Gibco). Selection was applied for 2 full weeks, after which cells were maintained in cell culture medium supplemented with 2 µg/ml puromycin.

Binder-mediated pulldowns

PER2-HaloTag knock-in U2OS cells stably expressing the binder of interest were grown to confluency in a 15 cm dish, then washed twice with ice-cold PBS, scraped, and collected in Falcon tubes. Following 400 g centrifugation for 5 minutes 4°C, the pelleted cells were lysed for 20 minutes in lysis buffer 50 mM HEPES, 125 mM KOAc, 5 mM Mg(OAc)2, 1% Triton X-100, 10 μM Zn(OAc)2 supplemented with 1x protease inhibitor cocktail (EDTA-free cOmplete, Roche) and 1x phosphatase inhibitor (PhosSTOP, Roche).

Lysates were then cleared at 10 000 g for 10 minutes. 25μL Strep-Tactin XT Sepharose beads (29401324, Cytiva) were equilibrated in the lysis buffer three times. After equilibration, lysates were loaded onto the beads and incubated for 2h at 4 °C with rotation. Following incubation the beads were washed 3 times in lysis buffer and 3 subsequent times in wash buffer (40 mM HEPES, 150 mM KOAc, 5 mM Mg(OAc)2. The beads were moved into fresh tubes for the final wash. The content of the beads was eluted using a solution of 2x NuPage LDS Sample Buffer (NP0008, Thermo Fisher Scientific) diluted in lysis buffer, supplemented with 5 mM DTT and heated at 95°C for 5 min.

SDS-PAGE and Immunoblotting

The resulting samples were run on NuPage Novex 4-12% Bis-Tris gradient gels (Thermo Fisher Scientific) using MOPS running buffer (MOPS-SDS0500, Formedium) at 100-200V for 1h. For immunoblotting, gels were briefly washed in Millipore water and protein transferred onto nitrocellulose membranes using the dry transfer iBlot2 system (IB21001, Thermo Fisher Scientific) with a standard (P0, 7 mins) protocol. Membranes were blocked in 5% w/w non-fat dried milk (Marvel) in TBS containing 0.1% Tween-20 (TBST) for 1 hour at room temperature. Membranes were then incubated in primary antibody: anti-HaloTag (1:1000, 2920S, Promega), anti-TwinStrepTag (1:1000, ab76949, abcam) diluted in blocking buffer (5% milk, TBST) at 4°C overnight. Subsequently, membranes were washed for 3x 10 mins (in TBST) and then incubated with the corresponding HRP-conjugated secondary antibody: anti-mouse HRP (1:5000, A4416, Sigma-Aldrich), anti-rabbit HRP (1:10000, A6154, Sigma-Aldrich) diluted in blocking buffer for 1h at room temperature. Excess secondary antibody was removed by washing 3x 10 mins (in TBST). Chemiluminescence detection was performed by incubating membranes with the Immobilon reagent (Millipore) and imaging using a ChemiDoc MP (Bio-Rad). Protein gels were stained for total protein using Colloidal Coomassie Blue Stain (Severn Biotech) according to the manufacturer's protocol.

Dynorphin A inhibition cAMP assay

Functional cAMP assay was performed according to previously described protocols(*53*–*55*) and using Chinese hamster ovarian (CHO) cells stably expressing human KOR. Briefly, 3,000 cells per 5 μL per well were seeded into a white 384-well plate and incubated with 5 μL of varying concentrations of binders prepared (4×) in a 1× stimulation buffer. The reaction mixture was incubated at 37°C for 45 min. Following, 5 uL of an EC80 concentration of dynorphin A 1-17 and 5 uL of forskolin (1 μM final) were added and incubated at 37°C for an additional 45 minutes. After adding 10 uL of Europium cryptate-labeled cAMP and cAMP d2-labeled antibody, respectively, and incubating the reaction mixture for 1 hour at room temperature, cAMP quantification was determined by measuring homogeneous time-resolved fluorescence resonance energy transfer on Neo2 plate reader using a ratio of 665/620 nm. The concentration response curve for dynorphin A 1-17 was generated in analogy to measuring binder antagonism with the exception of using 10 uL of cells per well.

Affinity enrichment of CTN4 analyzed by LC-MS

Affinity enrichment of CTN4

Designed 6xHis-tagged protein binders (CTN4_1b1, CTN4_1b2) were conjugated to Dynabeads™ His-Tag Isolation and Pulldown (Invitrogen, 10103D) according to the manufacturer's instructions. Functionalized magnetic beads (MBs) were subsequently blocked with 1% (w/v) BSA in PBS.

For affinity enrichment of CTN4 (NWLTIFILFPLK), 2 nmol of protein binders in 30 μL of beads slurry were used for capturing 0.2 nmol CTN4 spiked into 200 μL binding buffer (50 mM ammonium bicarbonate, 1 mM DTT, 0.1% Triton X-100, pH 8.0) or 200 μL dried blood spot (DBS) extract (a 3 mm DBS punch containing ~3.2 μL blood, extracted with same buffer above). Functionalized MBs were resuspended in spiked buffer or spiked DBS extract and incubated overnight with rotation at 4°C. After incubation, the supernatant was removed, and the beads were washed with 200 μL phosphate-buffered saline for three times, then resuspended in 30 μL elution buffer (30% acetonitrile, 1% formic acid) and incubated with shaking at 250 rpm for 10 minutes at room temperature to elute the peptides.

In the wash-and-loss experiment, CTN4 was spiked into a buffer and enriched using binder-MBs, and the unbound fraction of CTN4 from supernatant and washes were quantified. Non-functionalized MBs and random off-target MBs were used as negative controls to assess binder specificity. In the recovery experiment, CTN4 were spiked into buffer and DBS extract and enriched using binder-MBs, and recovery from both conditions were quantified. Equal volume of aliquots (10 µL) from all samples and standards were diluted in equal volume of LC solvent (200 µL 30% acetonitrile, 0.1% formic acid) prior to injection.

Sample description

Synthetic peptide CTN4 (NWLTIFILFPLK) was custom ordered from GenScript (NJ, USA), reconstituted at 1 mg/mL in 30% acetonitrile, 0.1% formic acid, and stored in aliquots at -20 °C. The dried blood spot (DBS) samples used were de-identified leftover clinical samples. The use of de-identified leftover clinical samples was reviewed by the University of Washington Human Subjects Division.

CTN4 affinity enrichment was performed using below series of samples:

Wash-and-loss experiment

Series A: Wash buffer (200 µL phosphate-buffered saline) spiked with 0.2 nmol CTN4 without the addition of beads or binders, served as the control sample representing 100% loss of CTN4.

Series B: 0.2 nmol CTN4 was spiked in 200 µL binding buffer and incubated with binder-MBs or control MBs for affinity enrichment. Supernatant was removed by magnetic separation and used to quantify the unbound CTN4.

Series C: 0.2 nmol CTN4 was spiked in 200 µL binding buffer and incubated with binder-MBs or control MBs for affinity enrichment. Followed by the supernatant removal, beads were subsequently washed in 200 µL wash buffer for 3 times. Each wash fraction was removed by magnetic separation and used to quantify the loss of CTN4.

Data treatment: Data processing was performed with TargetLynx (Waters, USA) within Masslynx. The peak area for spiked peptide was determined as the peak areas of the selected ion monitoring channel. Integrated peak areas were exported to Excel for further analysis. Unbound CTN4 during affinity enrichment were estimated using below equations.

Recovery experiment

Series D: elution buffer (30 µL 30% acetonitrile, 1% formic acid) spiked with 0.2 nmol CTN4 without the addition of beads or binders, served as the control sample representing 100% recovery of CTN4.

Series E: 0.2 nmol CTN4 was spiked in 200 µL binding buffer and incubated with binder-MBs for affinity enrichment. After supernatant removal and wash steps, the beads were resuspended in 30 µL elution buffer. The eluate was removed by magnetic separation and used to quantify the recovery of spiked CTN4 from buffer.

Series F: 0.2 nmol CTN4 was spiked in 200 µL DBS extract (a 3 mm diameter punch taken from a dried blood spot and extracted with buffer; contains approximately 3.2 µL of blood) and incubated with binder-MBs for affinity enrichment. After supernatant removal and wash steps, the beads were resuspended in 30 µL elution buffer. The eluate was removed by magnetic separation and used to quantify the recovery of spiked CTN4 recovery from DBS extract.

Data treatment: Data processing was performed with TargetLynx (Waters, USA) within Masslynx. The peak area for spiked peptide was determined as the peak areas of the selected ion monitoring channel. Integrated peak areas were exported to Excel for further analysis. CTN4 recovery from affinity enrichment were estimated using below equations.

LC-MS Analysis

Samples were transferred to clear glass screw neck total recovery vial for UPLC-MS analysis. The run was carried out on a Waters Xevo TQ-S micro mass spectrometer coupled to a Waters AQUITY UPLC I-Class system using SIM (selected ion monitoring) in ESI positive mode. The wash-and-loss experiment in buffer conditions was first conducted and ran with a 4.5 min LC gradient; after verified that CTN4 remains bound to the binder-MBs in buffer, the elution/recovery experiments was subsequently carried out with same MS conditions except the run time was shortened to 2.8 min. Mass spectrometry and liquid chromatography conditions are summarized below.

**Mass Spectrometry Conditions.**	
Source Polarity	ESI+
Capillary Voltage (V)	3500
Cone Voltage (V)	35
Source temperature (°C)	150
Desolvation temperature (°C)	500

Cone gas flow (L/hr)	20
Desolvation gas flow (L/hr)	1000
Peptide sequence	NWLTIFILFPLK
Q1 (m/z)	753.5

**Liquid Chromatography Conditions.**	
Mobile phase	Phase A: water with 0.1% formic acid<br>Phase B: acetonitrile with 0.1% formic acid
Wash solvent	Strong wash: acetonitrile with 0.1% formic acid<br>Weak wash: 90/10 (v/v) water/acetonitrile with 0.1% formic acid
Column	ACQUITY UPLC BEH C18 Column, 130Å, 1.7 µm, 2.1 mm x 50 mm
Temperature (°C)	22
Flow rate (mL/min)	0.4
Injection volume (µL)	10

Gradient	Wash-and-loss experiment:<br>The gradient started with 5% B and held until 0.5 min, increased linearly to 60% B at 3.8 min, then linearly to 100% B at 4 min, then switched back to 5% B at 4.2 min for re-equilibration and held until 4.5 min, with a flow rate of 0.4 mL/min. A divert valve was used to direct the LC eluent to the MS from 2.7 min to 3.8 min.<br>Recovery experiment:<br>The gradient started with 5% B and held until 0.5 min, increased linearly to 60% B at 2.0 min, then linearly to 100% B at 2.3 min, then switched back to 5% B at 2.5 min for re-equilibration and held until 2.8 min, with a flow rate of 0.4 mL/min. A divert valve was used to direct the LC eluent into the MS from 1.2 min to 2.3 min.

NMR sample preparation and data collection of dynorphin-binder complex data

The plasmid encoding for 6xHis-SUMO-dynorphin was purchased from Genscript (based on the pET15_SUMO2_NESG vector) and expressed in *E. coli* BL21 (DE3) grown in MJ9 media with $^{15}NH_4SO4$ and ^{13}C-glucose[17] and 50 ug/ml Carbencillin. Briefly, 1 L of MJ9 media was inoculated with 25 ml of overnight grown starter culture in an incubator shaker set at 37°C, 200 RPM. Expression was induced with 0.25 mM of IPTG at 18°C and for 18 h. The next day, cells were pelleted by centrifugation, resuspended in Tris buffer (50 mM Tris-HCl pH 7.5, 500 mM NaCl) with 5 mM imidazole, and lysed by mild sonication. The lysate was clarified by centrifugation and loaded onto an IMAC column (Cytiva 5 ml HisTrap HP column). After washing and eluting with Tris buffer containing 250 mM imidazole, the 6xHis-SUMO-dynorphin fusion protein was further purified by SEC (Cytiva HiLoad 16/600 Superdex 75 pg) using an ÄKTA pure chromatography system (Cytiva) followed by overnight cleavage with SUMO protease at room temperature. The solution containing SUMO protease, the SUMO tag, and cleaved dynorphin was lyophilized, resuspended in cold methanol, and centrifuged at high speed. The methanol supernatant was vacuum dried, similar to a previously described peptide purification protocol[18]. Final purification was performed by reverse-phase chromatography with an acetonitrile/water gradient (Resource RPC, 3 mL, Cytiva) on an ÄKTA pure system as described previously for dynorphin[19], and the molecular weight was confirmed using MALDI-TOF mass spectrometry. Eluted samples

were lyophilized,stored at -80°C, and resuspended to 1-4 mM (300 ul) in 20 mM phosphate buffer pH 6.0, 150 mM NaCl prior to complex formation.

His_6-tagged protein binders DYNA_1b7 and DYNA_2b2 were cultured in LB broth and purified using IMAC and SEC, similarly as for dynorphin A. After elution into 20 mM phosphate buffer pH 6.0, 150 mM NaCl, samples were concentrated to 20-100 uM using Vivaspin centrifugal concentrators (Sartorius, 5 kDa MWCO). Binder-dynorphin complexes were prepared by mixing $^{15}N^{13}C$-labeled dynorphin to a solution of purified binders. After incubation overnight at 4°C, the complex was further purified by SEC (Superdex 75) with the same buffer, and the monomer elution fraction was concentrated to 100-250 uM by centrifugal filter concentration. The sizes and stability of dynorphin and its binder were confirmed by MALDI-TOF MS. NMR samples were prepared with 5% D_2O, and 300 uL was loaded into 5 mm Shigemi tubes.

NMR spectra were collected at 25 or 35°C with a 5 mm TCI CryoProbe on a Bruker Avance III 600 MHz or Avance Neo 800 MHz spectrometer. Chemical shift assignments for dynorphin complexes were obtained from standard spectra including 1D ^{1}H spectra, 2D ^{1}H-^{15}N SOFAST HMQC and ^{1}H-^{13}C HSQC (aliphatic and aromatic) spectra, and 2D (free) or 3D (bound to DYNA_1b7 or DYNA_2b2) HNCO, HNCA, HNcoCA, HNCACB, ^{15}N-edited NOESY (100 ms), and ^{1}H-^{13}C hCCH TOCSY (11 ms) spectra. 3D 15N-edited NOESY data was collected at 800MHz with a non-uniform sampling rate of 50%. Chemical shift assignments for free dynorphin (4 mM) were obtained from 2D HC faces of standard 3D HNCA, HNcoCA, HNCACB, HNcoCACB, HNCO, ^{15}N-edited HccONH and CccoNH spectra and HH faces of ^{15}N-edited TOCSY (60 ms) and ^{1}H-^{13}C hCCH TOCSY (11 ms), as 3D data was not required. All spectra were referenced to internal DSS. Amide proton chemical shift temperature dependence was measured from 2D 1H-15N SOFAST HMQC spectra at temperatures ranging from 10°C to 35°C in 5°C intervals. Temperature coefficients ($\Delta\delta HN/\Delta T$) were derived through linear regression using Microsoft Excel.

Spectra were processed using TopSpin 4.0 (Bruker) or NMRPipe software(*55*) and visualized using NMRFAM-SPARKY(*56*). Non-uniform sampling data were processed using the SMILE package(*57*) package integrated with NMRPipe. Secondary structure propensities were calculated using TALOS-N from NMR backbone (and CB) chemical shifts(*58*) for unbound dynorphin, DYNA_1b7-, and DYNA_2b2-bound states. Positive (green) bars indicate β-strand, while negative (gold) indicate helical propensity, with predicted β-strand secondary structure shown as green arrows above this data. Chemical-shift based RMSF (Å) ($RMSF_{RCI}$)was calculated by scaling the random coil index by 12.7(*60*, *61*).

X-ray crystallography

Crystallization experiments were conducted using the sitting drop vapor diffusion method. Initial crystallization trials were set up in 200 nL drops using the 96-well plate format at 20˚C. Crystallization plates were set up using a Mosquito LCP from SPT Labtech, then imaged using UVEX microscopes and UVEX PS-256 from JAN Scientific. Diffraction quality crystals formed in 0.1 M Phosphate/citrate pH 4.2 and 40% v/v PEG 300 for DNYA_1b7-1. Diffraction quality crystals formed in 0.1 M Sodium acetate pH 4.6 and 25% (v/v) PEG 550 MME for DA7-2.

Diffraction data was collected at the National Synchrotron Light Source II on beamline 17-ID-1 (AMF) for DYNA_1b7-1 and at the Advanced Light Source beamline 821 for DA7-2. X-ray intensities and data reduction were evaluated and integrated using XDS(*61*) and merged/scaled using Pointless/Aimless in the CCP4 program suite(*62*). Structure determination and refinement starting phases were obtained by molecular replacement using Phaser(*62*) using the designed model for the structures. Following molecular replacement, the models were improved using phenix.autobuild(*63*); with rebuild-in-place to false, and using simulated annealing. Structures were refined in Phenix(*63*). Model building was performed using COOT(*64*). The final model was evaluated using MolProbity(*65*). Data collection and refinement statistics are recorded in the statistics table below. Data deposition, atomic coordinates, and structure factors reported in this paper have been deposited in the Protein Data Bank (PDB), http://www.rcsb.org/ with accession code 9CCE and 9CCF.

Crystallographic data collection and refinement statistics table

	DYNA_1b7 (PDB Code: 9CCE)	DA7-2 (PDB Code: 9CCF)
Resolution range	28.84 - 3.15 (3.23 - 3.15)	47.76 - 4.00 (4.47 - 4.0)
Space group	$P\ 2_1$	$P\ 2_1$
Unit cell	44.43, 67.78, 68.36; 90.00, 98.03, 90.00	43.42, 67.21, 68.36; 90.00, 96.75, 90.00
Unique reflections	6972 (517)	3361 (941)
Multiplicity	3.1 (3.1)	5.0 (5.1)
Completeness (%)	98.3 (96.8)	99.5 (99.8)
Mean I/sigma(I)	5.6 (1.3)	11.4 (6.5)
Wilson B-factor	84.85	129.92
R-merge	0.138 (0.658)	0.047 (0.118)
R-pim	0.102 (0.498)	0.047 (0.119)
$CC_{1/2}$	0.989 (0.790)	0.997 (0.979)
Reflections used in refinement	6951 (511)	3343 (900)
R-work	0.2626 (0.3187)	0.24.23 (0.2526)
R-free	0.3080 (0.3800)	0.29.63 (0.3408)
Number of non-hydrogen atoms	3355	3221
macromolecules	3355	3221
Protein residues	410	394
RMS(bonds)	0.002	0.002

RMS(angles)	0.42	0.429
Ramachandran favored (%)	96.97	96.83
Ramachandran allowed (%)	3.03	3.17
Ramachandran outliers (%)	0.00	0.00
Average B-factor	81	126
macromolecules	81	126

The highest-resolution shells are shown in parentheses.

References and Notes

1. M. L. Chiu, D. R. Goulet, A. Teplyakov, G. L. Gilliland, Antibody structure and function: The basis for engineering therapeutics. *Antibodies* **8**, 55 (2019). doi:10.3390/antib8040055 Medline

2. J. H. Lee, R. Yin, G. Ofek, B. G. Pierce, Structural features of antibody-peptide recognition. *Front. Immunol.* **13**, 910367 (2022). doi:10.3389/fimmu.2022.910367 Medline

3. A. L. Nelson, E. Dhimolea, J. M. Reichert, Development trends for human monoclonal antibody therapeutics. *Nat. Rev. Drug Discov.* **9**, 767–774 (2010). doi:10.1038/nrd3229 Medline

4. M. A. S. Perez, M. A. Cuendet, U. F. Röhrig, O. Michielin, V. Zoete, Structural prediction of peptide-MHC binding modes. *Methods Mol. Biol.* **2405**, 245–282 (2022). doi:10.1007/978-1-0716-1855-4_13 Medline

5. N. Zeytuni, R. Zarivach, Structural and functional discussion of the tetra-trico-peptide repeat, a protein interaction module. *Structure* **20**, 397–405 (2012). doi:10.1016/j.str.2012.01.006 Medline

6. P. Ernst, A. Plückthun, Advances in the design and engineering of peptide-binding repeat proteins. *Biol. Chem.* **398**, 23–29 (2017). doi:10.1515/hsz-2016-0233 Medline

7. K. Wu, H. Bai, Y.-T. Chang, R. Redler, K. E. McNally, W. Sheffler, T. J. Brunette, D. R. Hicks, T. E. Morgan, T. J. Stevens, A. Broerman, I. Goreshnik, M. DeWitt, C. M. Chow, Y. Shen, L. Stewart, E. Derivery, D. A. Silva, G. Bhabha, D. C. Ekiert, D. Baker, De novo design of modular peptide-binding proteins by superhelical matching. *Nature* **616**, 581–589 (2023). doi:10.1038/s41586-023-05909-9 Medline

8. S. Vázquez Torres, P. J. Y. Leung, P. Venkatesh, I. D. Lutz, F. Hink, H.-H. Huynh, J. Becker, A. H.-W. Yeh, D. Juergens, N. R. Bennett, A. N. Hoofnagle, E. Huang, M. J. MacCoss, M. Expòsit, G. R. Lee, A. K. Bera, A. Kang, J. De La Cruz, P. M. Levine, X. Li, M. Lamb, S. R. Gerben, A. Murray, P. Heine, E. N. Korkmaz, J. Nivala, L. Stewart, J. L. Watson, J. M. Rogers, D. Baker, De novo design of high-affinity binders of bioactive helical peptides. *Nature* **626**, 435–442 (2024). doi:10.1038/s41586-023-06953-1 Medline

9. D. D. Sahtoe, E. A. Andrzejewska, H. L. Han, E. Rennella, M. M. Schneider, G. Meisl, M. Ahlrichs, J. Decarreau, H. Nguyen, A. Kang, P. Levine, M. Lamb, X. Li, A. K. Bera, L. E. Kay, T. P. J. Knowles, D. Baker, Design of amyloidogenic peptide traps. *Nat. Chem. Biol.* **20**, 981–990 (2024). doi:10.1038/s41589-024-01578-5 Medline

10. C. Liu, K. Wu, H. Choi, H. Han, X. Zhang, J. L. Watson, S. Shijo, A. K. Bera, A. Kang, E. Brackenbrough, B. Coventry, D. R. Hick, A. N. Hoofnagle, P. Zhu, X. Li, J. Decarreau, S. R. Gerben, W. Yang, X. Wang, M. Lamp, A. Murray, M. Bauer, D. Baker, Diffusing protein binders to intrinsically disordered proteins. bioRxiv 2024.07.16.603789v1 [Preprint] (2024); doi:10.1101/2024.07.16.603789v1.

11. J. L. Watson, D. Juergens, N. R. Bennett, B. L. Trippe, J. Yim, H. E. Eisenach, W. Ahern, A. J. Borst, R. J. Ragotte, L. F. Milles, B. I. M. Wicky, N. Hanikel, S. J. Pellock, A. Courbet, W. Sheffler, J. Wang, P. Venkatesh, I. Sappington, S. V. Torres, A. Lauko, V. De Bortoli, E. Mathieu, S. Ovchinnikov, R. Barzilay, T. S. Jaakkola, F. DiMaio, M.

Baek, D. Baker, De novo design of protein structure and function with RFdiffusion. *Nature* **620**, 1089–1100 (2023). doi:10.1038/s41586-023-06415-8 Medline

12. R. Rozbeh, K. Forchhammer, Split NanoLuc technology allows quantitation of interactions between PII protein and its receptors with unprecedented sensitivity and reveals transient interactions. *Sci. Rep.* **11**, 12535 (2021). doi:10.1038/s41598-021-91856-2 Medline

13. X. Li, M. E. McGee-Lawrence, M. Decker, J. J. Westendorf, The Ewing's sarcoma fusion protein, EWS-FLI, binds Runx2 and blocks osteoblast differentiation. *J. Cell. Biochem.* **111**, 933–943 (2010). doi:10.1002/jcb.22782 Medline

14. S. P. Selvanathan, G. T. Graham, H. V. Erkizan, U. Dirksen, T. G. Natarajan, A. Dakic, S. Yu, X. Liu, M. T. Paulsen, M. E. Ljungman, C. H. Wu, E. R. Lawlor, A. Üren, J. A. Toretsky, Oncogenic fusion protein EWS-FLI1 is a network hub that regulates alternative splicing. *Proc. Natl. Acad. Sci. U.S.A.* **112**, E1307–E1316 (2015). doi:10.1073/pnas.1500536112 Medline

15. G. Flores, P. J. Grohar, One oncogene, several vulnerabilities: EWS/FLI targeted therapies for Ewing sarcoma. *J. Bone Oncol.* **31**, 100404 (2021). doi:10.1016/j.jbo.2021.100404 Medline

16. J. Tan, H. Cho, T. Pholcharee, L. S. Pereira, S. Doumbo, D. Doumtabe, B. J. Flynn, A. Schön, S. Kanatani, S. O. Aylor, D. Oyen, R. Vistein, L. Wang, M. Dillon, J. Skinner, M. Peterson, S. Li, A. H. Idris, A. Molina-Cruz, M. Zhao, L. R. Olano, P. J. Lee, A. Roth, P. Sinnis, C. Barillas-Mury, K. Kayentao, A. Ongoiba, J. R. Francica, B. Traore, I. A. Wilson, R. A. Seder, P. D. Crompton, Functional human IgA targets a conserved site on malaria sporozoites. *Sci. Transl. Med.* **13**, eabg2344 (2021). doi:10.1126/scitranslmed.abg2344 Medline

17. A. Hang, Y.-J. Wang, L. He, J.-G. Liu, The role of the dynorphin/κ opioid receptor system in anxiety. *Acta Pharmacol. Sin.* **36**, 783–790 (2015). doi:10.1038/aps.2015.32 Medline

18. S. Podvin, T. Yaksh, V. Hook, The emerging role of spinal dynorphin in chronic pain: A therapeutic perspective. *Annu. Rev. Pharmacol. Toxicol.* **56**, 511–533 (2016). doi:10.1146/annurev-pharmtox-010715-103042 Medline

19. E. B. Margolis, M. G. Moulton, P. S. Lambeth, M. J. O'Meara, The life and times of endogenous opioid peptides: Updated understanding of synthesis, spatiotemporal dynamics, and the clinical impact in alcohol use disorder. *Neuropharmacology* **225**, 109376 (2023). doi:10.1016/j.neuropharm.2022.109376 Medline

20. C. O'Connor, K. L. White, N. Doncescu, T. Didenko, B. L. Roth, G. Czaplicki, R. C. Stevens, K. Wüthrich, A. Milon, NMR structure and dynamics of the agonist dynorphin peptide bound to the human kappa opioid receptor. *Proc. Natl. Acad. Sci. U.S.A.* **112**, 11852–11857 (2015). doi:10.1073/pnas.1510117112 Medline

21. Y. Wang, Y. Zhuang, J. F. DiBerto, X. E. Zhou, G. P. Schmitz, Q. Yuan, M. K. Jain, W. Liu, K. Melcher, Y. Jiang, B. L. Roth, H. E. Xu, Structures of the entire human opioid receptor family. *Cell* **186**, 413–427.e17 (2023). doi:10.1016/j.cell.2022.12.026 Medline

22. E. Derivery, C. Sousa, J. J. Gautier, B. Lombard, D. Loew, A. Gautreau, The Arp2/3 activator WASH controls the fission of endosomes through a large multiprotein complex. *Dev. Cell* **17**, 712–723 (2009). doi:10.1016/j.devcel.2009.09.010 Medline

23. T. S. Gomez, D. D. Billadeau, A FAM21-containing WASH complex regulates retromer-dependent sorting. *Dev. Cell* **17**, 699–711 (2009). doi:10.1016/j.devcel.2009.09.009 Medline

24. M. Putker, D. C. S. Wong, E. Seinkmane, N. M. Rzechorzek, A. Zeng, N. P. Hoyle, J. E. Chesham, M. D. Edwards, K. A. Feeney, R. Fischer, N. Peschel, K.-F. Chen, M. Vanden Oever, R. S. Edgar, C. P. Selby, A. Sancar, J. S. O'Neill, CRYPTOCHROMES confer robustness, not rhythmicity, to circadian timekeeping. *EMBO J.* **40**, e106745 (2021). doi:10.15252/embj.2020106745 Medline

25. A. Mihut, J. S. O'Neill, C. L. Partch, P. Crosby, PERspectives on circadian cell biology. *Philos. Trans. R. Soc. B* **380**, 20230483 (2025). doi:10.1098/rstb.2023.0483 Medline

26. M. Al-Haggar, Cystinosis as a lysosomal storage disease with multiple mutant alleles: Phenotypic-genotypic correlations. *World J. Nephrol.* **2**, 94–102 (2013). Medline

27. M. A. Elmonem, K. R. Veys, N. A. Soliman, M. van Dyck, L. P. van den Heuvel, E. Levtchenko, Cystinosis: A review. *Orphanet J. Rare Dis.* **11**, 47 (2016). doi:10.1186/s13023-016-0426-y Medline

28. X. Liu, A. Chan, C.-H. Tai, T. Andresson, I. Pastan, Multiple proteases are involved in mesothelin shedding by cancer cells. *Commun. Biol.* **3**, 728 (2020). doi:10.1038/s42003-020-01464-5 Medline

29. P. Ernst, F. Zosel, C. Reichen, D. Nettels, B. Schuler, A. Plückthun, Structure-guided design of a peptide lock for modular peptide binders. *ACS Chem. Biol.* **15**, 457–468 (2020). doi:10.1021/acschembio.9b00928 Medline

30. X. Wang, Z. Xu, Z. Tian, X. Zhang, D. Xu, Q. Li, J. Zhang, T. Wang, The EF-1α promoter maintains high-level transgene expression from episomal vectors in transfected CHO-K1 cells. *J. Cell. Mol. Med.* **21**, 3044–3054 (2017). doi:10.1111/jcmm.13216 Medline

31. H. J. Dyson, P. E. Wright, Intrinsically unstructured proteins and their functions. *Nat. Rev. Mol. Cell Biol.* **6**, 197–208 (2005). doi:10.1038/nrm1589 Medline

32. K. Fujiwara, H. Toda, M. Ikeguchi, Dependence of α-helical and β-sheet amino acid propensities on the overall protein fold type. *BMC Struct. Biol.* **12**, 18 (2012). doi:10.1186/1472-6807-12-18 Medline

33. S. Sipeki, K. Koprivanacz, T. Takács, A. Kurilla, L. László, V. Vas, L. Buday, Novel roles of SH2 and SH3 domains in lipid binding. *Cells* **10**, 1191 (2021). doi:10.3390/cells10051191 Medline

34. J. Z. Zhang, X. Li, C. Liu, H. Jiang, K. Wu, D. Baker, De novo design of Ras isoform selective binders. bioRxiv 2024.08.29.610300v3 [Preprint] (2024); doi:10.1101/2024.08.29.610300v3.

35. J. Lv, P. Li, Mesothelin as a biomarker for targeted therapy. *Biomark. Res.* **7**, 18 (2019). doi:10.1186/s40364-019-0169-8 Medline

36. T. Sasaki, S. J. Rodig, L. R. Chirieac, P. A. Jänne, The biology and treatment of EML4-ALK non-small cell lung cancer. *Eur. J. Cancer* **46**, 1773–1780 (2010). doi:10.1016/j.ejca.2010.04.002 Medline

37. S. Aiyer, G. V. T. Swapna, L.-C. Ma, G. Liu, J. Hao, G. Chalmers, B. C. Jacobs, G. T. Montelione, M. J. Roth, A common binding motif in the ET domain of BRD3 forms polymorphic structural interfaces with host and viral proteins. *Structure* **29**, 886–898.e6 (2021). doi:10.1016/j.str.2021.01.010 Medline

38. C. Erady, K. Amin, T. O. A. E. Onilogbo, J. Tomasik, R. Jukes-Jones, Y. Umrania, S. Bahn, S. Prabakaran, Novel open reading frames in human accelerated regions and transposable elements reveal new leads to understand schizophrenia and bipolar disorder. *Mol. Psychiatry* **27**, 1455–1468 (2022). doi:10.1038/s41380-021-01405-6 Medline

39. A. Varabyou, B. Erdogdu, S. L. Salzberg, M. Pertea, Investigating open reading frames in known and novel transcripts using ORFanage. *Nat. Comput. Sci.* **3**, 700–708 (2023). doi:10.1038/s43588-023-00496-1 Medline

40. D. R. Hicks, drhicks/Kejia_peptide_binders: release for manuscript review, Zenodo (2025); https://zenodo.org/records/14829674.

41. A. L. Ptaszek, J. Li, R. Konrat, G. Platzer, T. Head-Gordon, UCBShift 2.0: Bridging the gap from backbone to side chain protein chemical shift prediction for protein structures. *J. Am. Chem. Soc.* **146**, 31733–31745 (2024). doi:10.1021/jacs.4c10474 Medline

42. J. Dauparas, I. Anishchenko, N. Bennett, H. Bai, R. J. Ragotte, L. F. Milles, B. I. M. Wicky, A. Courbet, R. J. de Haas, N. Bethel, P. J. Y. Leung, T. F. Huddy, S. Pellock, D. Tischer, F. Chan, B. Koepnick, H. Nguyen, A. Kang, B. Sankaran, A. K. Bera, N. P. King, D. Baker, Robust deep learning-based protein sequence design using ProteinMPNN. *Science* **378**, 49–56 (2022). doi:10.1126/science.add2187 Medline

43. L. Cao, B. Coventry, I. Goreshnik, B. Huang, W. Sheffler, J. S. Park, K. M. Jude, I. Marković, R. U. Kadam, K. H. G. Verschueren, K. Verstraete, S. T. R. Walsh, N. Bennett, A. Phal, A. Yang, L. Kozodoy, M. DeWitt, L. Picton, L. Miller, E.-M. Strauch, N. D. DeBouver, A. Pires, A. K. Bera, S. Halabiya, B. Hammerson, W. Yang, S. Bernard, L. Stewart, I. A. Wilson, H. Ruohola-Baker, J. Schlessinger, S. Lee, S. N. Savvides, K. C. Garcia, D. Baker, Design of protein-binding proteins from the target structure alone. *Nature* **605**, 551–560 (2022). doi:10.1038/s41586-022-04654-9 Medline

44. N. R. Bennett, B. Coventry, I. Goreshnik, B. Huang, A. Allen, D. Vafeados, Y. P. Peng, J. Dauparas, M. Baek, L. Stewart, F. DiMaio, S. De Munck, S. N. Savvides, D. Baker, Improving de novo protein binder design with deep learning. *Nat. Commun.* **14**, 2625 (2023). doi:10.1038/s41467-023-38328-5 Medline

45. J. Jumper, R. Evans, A. Pritzel, T. Green, M. Figurnov, O. Ronneberger, K. Tunyasuvunakool, R. Bates, A. Žídek, A. Potapenko, A. Bridgland, C. Meyer, S. A. A. Kohl, A. J. Ballard, A. Cowie, B. Romera-Paredes, S. Nikolov, R. Jain, J. Adler, T. Back, S. Petersen, D. Reiman, E. Clancy, M. Zielinski, M. Steinegger, M. Pacholska, T. Berghammer, S. Bodenstein, D. Silver, O. Vinyals, A. W. Senior, K. Kavukcuoglu, P. Kohli, D. Hassabis, Highly accurate protein structure prediction with AlphaFold. *Nature* **596**, 583–589 (2021). doi:10.1038/s41586-021-03819-2 Medline

46. C. Reichen, S. Hansen, C. Forzani, A. Honegger, S. J. Fleishman, T. Zhou, F. Parmeggiani, P. Ernst, C. Madhurantakam, C. Ewald, P. R. E. Mittl, O. Zerbe, D. Baker, A. Caflisch, A. Plückthun, Computationally designed armadillo repeat proteins for modular peptide

recognition. *J. Mol. Biol.* **428**, 4467–4489 (2016). doi:10.1016/j.jmb.2016.09.012 Medline

47. C. Reichen, S. Hansen, A. Plückthun, Modular peptide binding: From a comparison of natural binders to designed armadillo repeat proteins. *J. Struct. Biol.* **185**, 147–162 (2014). doi:10.1016/j.jsb.2013.07.012 Medline

48. H. Jiang, K. M. Jude, K. Wu, J. Fallas, G. Ueda, T. J. Brunette, D. R. Hicks, H. Pyles, A. Yang, L. Carter, M. Lamb, X. Li, P. M. Levine, L. Stewart, K. C. Garcia, D. Baker, De novo design of buttressed loops for sculpting protein functions. *Nat. Chem. Biol.* **20**, 974–980 (2024). doi:10.1038/s41589-024-01632-2 Medline

49. R. Evans, M. O'Neill, A. Pritzel, N. Antropova, A. Senior, T. Green, A. Žídek, R. Bates, S. Blackwell, J. Yim, O. Ronneberger, S. Bodenstein, M. Zielinski, A. Bridgland, A. Potapenko, A. Cowie, K. Tunyasuvunakool, R. Jain, E. Clancy, P. Kohli, J. Jumper, D. Hassabis, Protein complex prediction with AlphaFold-Multimer, bioRxiv 2021.10.04.463034 [Preprint] (2021). doi:10.1101/2021.10.04.463034.

50. S. R. Eddy, Where did the BLOSUM62 alignment score matrix come from? *Nat. Biotechnol.* **22**, 1035–1036 (2004). doi:10.1038/nbt0804-1035 Medline

51. N. London, B. Raveh, E. Cohen, G. Fathi, O. Schueler-Furman, Rosetta FlexPepDock web server—High resolution modeling of peptide-protein interactions. *Nucleic Acids Res.* **39**, W249-53 (2011). doi:10.1093/nar/gkr431 Medline

52. B. Coventry, D. Baker, Protein sequence optimization with a pairwise decomposable penalty for buried unsatisfied hydrogen bonds. *PLOS Comput. Biol.* **17**, e1008061 (2021). doi:10.1371/journal.pcbi.1008061 Medline

53. A. D. Beale, N. M. Rzechorzek, A. Mihut, A. Zeng, N. J. Smyllie, V. Pilorz, R. Richardson, M. F. Bertlesen, N. R. James, S. V. Fazal, Z. Voysey, J. Pelletier, P. Crosby, S. Y. Peak-Chew, M. A. Lancaster, R. A. Hut, J. S. O'Neill, Thermosensitivity of translation underlies the mammalian nocturnal-diurnal switch, bioRxiv 2023.06.22.546020 [Preprint] (2023). doi:10.1101/2023.06.22.546020.

54. E. Muratspahić, N. Tomašević, J. Koehbach, L. Duerrauer, S. Hadžić, J. Castro, G. Schober, S. Sideromenos, R. J. Clark, S. M. Brierley, D. J. Craik, C. W. Gruber, Design of a stable cyclic peptide analgesic derived from sunflower seeds that targets the κ-opioid receptor for the treatment of chronic abdominal pain. *J. Med. Chem.* **64**, 9042–9055 (2021). doi:10.1021/acs.jmedchem.1c00158 Medline

55. E. Muratspahić, A. M. White, C. I. Ciotu, N. Hochrainer, N. Tomašević, J. Koehbach, R. J. Lewis, M. Spetea, M. J. M. Fischer, D. J. Craik, C. W. Gruber, Development of a selective peptide κ-opioid receptor antagonist by late-stage functionalization with cysteine staples. *J. Med. Chem.* **66**, 11843–11854 (2023). doi:10.1021/acs.jmedchem.3c00426 Medline

56. E. Muratspahić, K. Deibler, J. Han, N. Tomašević, K. B. Jadhav, A.-L. Olivé-Marti, N. Hochrainer, R. Hellinger, J. Koehbach, J. F. Fay, M. H. Rahman, L. Hegazy, T. W. Craven, B. R. Varga, G. Bhardwaj, K. Appourchaux, S. Majumdar, M. Muttenthaler, P. Hosseinzadeh, D. J. Craik, M. Spetea, T. Che, D. Baker, C. W. Gruber, Design and

structural validation of peptide-drug conjugate ligands of the kappa-opioid receptor. *Nat. Commun.* **14**, 8064 (2023). doi:10.1038/s41467-023-43718-w Medline

57. F. Delaglio, S. Grzesiek, G. W. Vuister, G. Zhu, J. Pfeifer, A. Bax, NMRPipe: A multidimensional spectral processing system based on UNIX pipes. *J. Biomol. NMR* **6**, 277–293 (1995). doi:10.1007/BF00197809 Medline
58. W. Lee, M. Tonelli, J. L. Markley, NMRFAM-SPARKY: Enhanced software for biomolecular NMR spectroscopy. *Bioinformatics* **31**, 1325–1327 (2015). doi:10.1093/bioinformatics/btu830 Medline
59. J. Ying, F. Delaglio, D. A. Torchia, A. Bax, Sparse multidimensional iterative lineshape-enhanced (SMILE) reconstruction of both non-uniformly sampled and conventional NMR data. *J. Biomol. NMR* **68**, 101–118 (2017). doi:10.1007/s10858-016-0072-7 Medline
60. Y. Hen, A. Bax, "Protein Structural Information Derived from NMR Chemical Shift with the Neural Network Program TALOS-N" in *Methods in Molecular Biology*, H. Cartwright, Ed. (Springer, 2015), vol. 1260, pp. 17–32. vol 1260
61. M. V. Berjanskii, D. S. Wishart, A simple method to predict protein flexibility using secondary chemical shifts. *J. Am. Chem. Soc.* **127**, 14970–14971 (2005). doi:10.1021/ja054842f Medline
62. M. V. Berjanskii, D. S. Wishart, The RCI server: Rapid and accurate calculation of protein flexibility using chemical shifts. *Nucleic Acids Res.* **35**, W531-7 (2007). doi:10.1093/nar/gkm328 Medline
63. W. Kabsch, XDS. *Acta Crystallogr. D.* **66**, 125–132 (2010). doi:10.1107/S0907444909047337 Medline
64. A. J. McCoy, R. W. Grosse-Kunstleve, P. D. Adams, M. D. Winn, L. C. Storoni, R. J. Read, Phaser crystallographic software. *J. Appl. Cryst.* **40**, 658–674 (2007). doi:10.1107/S0021889807021206 Medline
65. P. D. Adams, P. V. Afonine, G. Bunkóczi, V. B. Chen, I. W. Davis, N. Echols, J. J. Headd, L.-W. Hung, G. J. Kapral, R. W. Grosse-Kunstleve, A. J. McCoy, N. W. Moriarty, R. Oeffner, R. J. Read, D. C. Richardson, J. S. Richardson, T. C. Terwilliger, P. H. Zwart, PHENIX: A comprehensive Python-based system for macromolecular structure solution. *Acta Crystallogr. D.* **66**, 213–221 (2010). doi:10.1107/S0907444909052925 Medline
66. P. Emsley, K. Cowtan, Coot: Model-building tools for molecular graphics. *Acta Crystallogr. D.* **60**, 2126–2132 (2004). doi:10.1107/S0907444904019158 Medline
67. C. J. Williams, J. J. Headd, N. W. Moriarty, M. G. Prisant, L. L. Videau, L. N. Deis, V. Verma, D. A. Keedy, B. J. Hintze, V. B. Chen, S. Jain, S. M. Lewis, W. B. Arendall 3rd, J. Snoeyink, P. D. Adams, S. C. Lovell, J. S. Richardson, D. C. Richardson, MolProbity: More and better reference data for improved all-atom structure validation. *Protein Sci.* **27**, 293–315 (2018). doi:10.1002/pro.3330 Medline
